# Supplementary material for: Integrating Gender-Affirming Care in a Medical Spanish Endocrine System Curriculum
Source: MedEdPORTAL. 2024 Oct 23;20:11456. doi: 10.15766/mep_2374-8265.11456 (PMC11496385; doi:10.15766/mep_2374-8265.11456)
Supplement: Supplementary file 1 — Facilitator Guide.docxLesson 1 Presentation.pptxLesson 2 Presentation.pptxLesson 3 Presentation.pptxLesson 1 Clinical Endocrine Checklist.docxLesson 2 Clinical Endocrine Checklist.docxLesson 3 Clinical Endocrine Checklist.docxLesson 1 SP Case.docxLesson 2 SP Case.docxLesson 3 SP Case.docxPre-Post Confidence Survey.docxPre-Post Spanish Endocrine Test.docxOSCE SP Diabetic Case.docxOSCE Door Note.docxOSCE Clinical Checklist Diabetic Encounter.docxOSCE Language Rubric for Diabetic Encounter.docx [file mep_2374-8265.11456-s001.zip › B. Lesson 1 Presentation.pptx]

## Slide 1
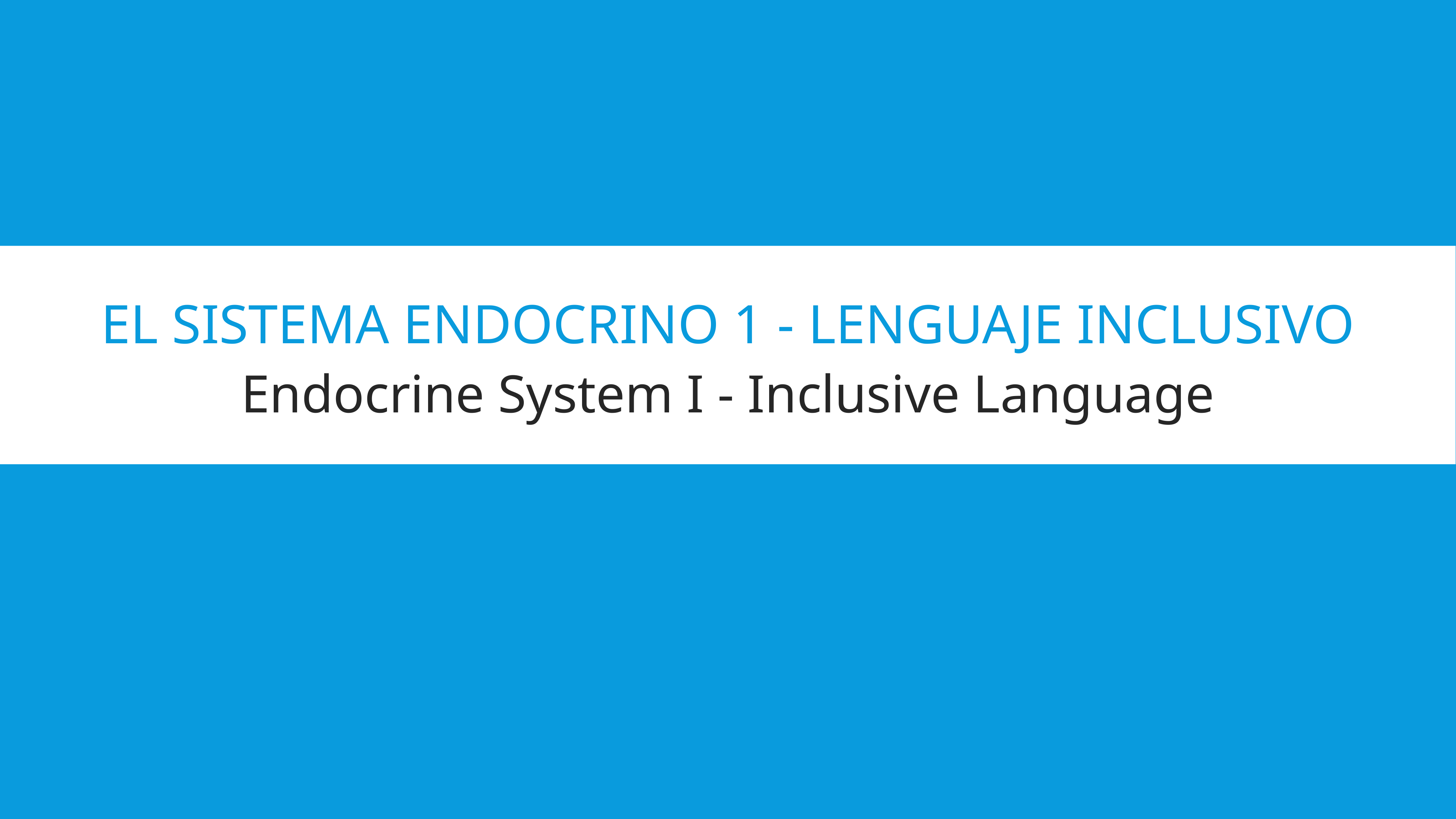

# El sistema endocrino 1 - lenguaje inclusivo
Endocrine System I - Inclusive Language

## Slide 2
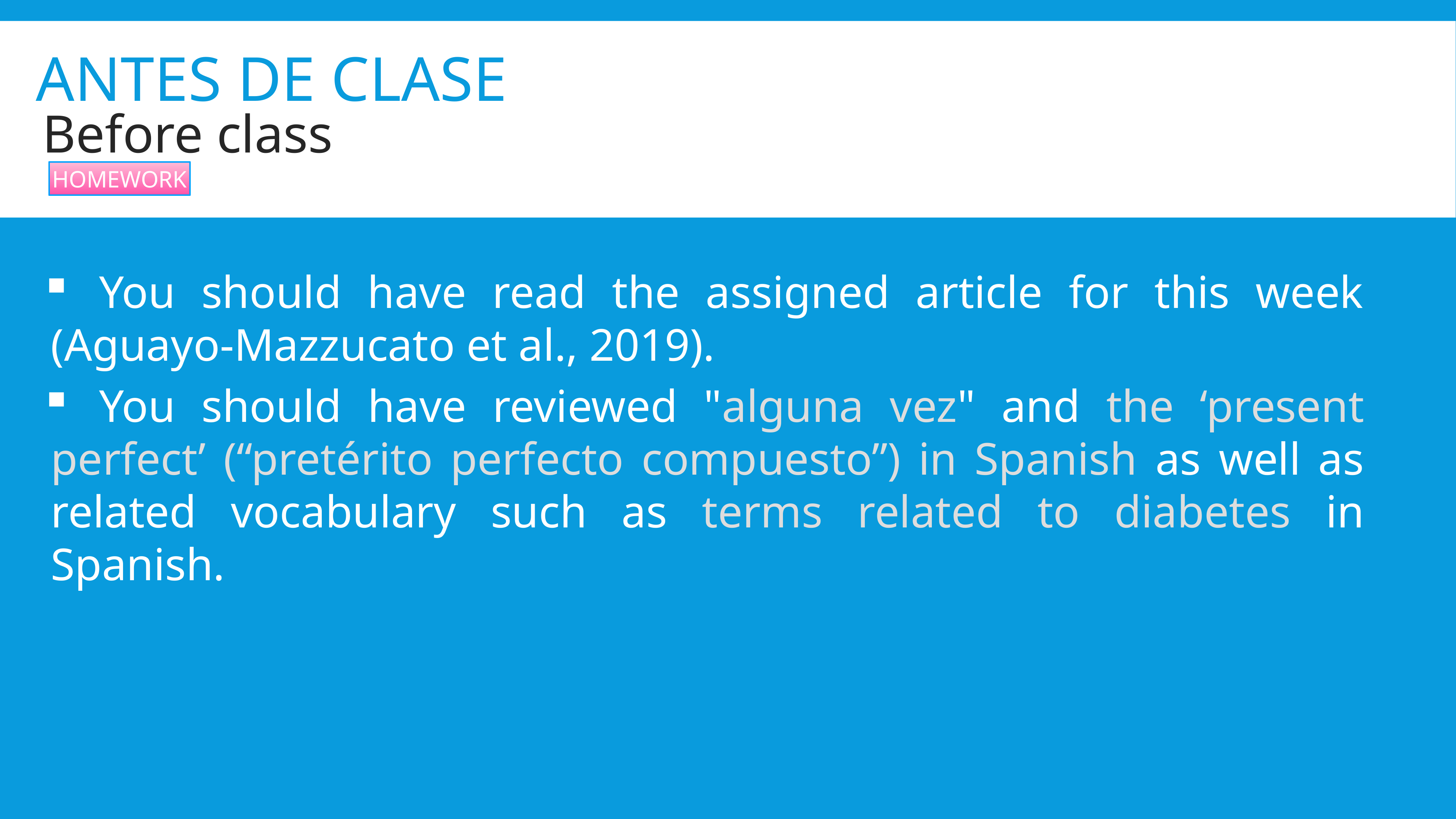

# Antes de clase
Before class
HOMEWORK
 You should have read the assigned article for this week (Aguayo-Mazzucato et al., 2019).
 You should have reviewed "alguna vez" and the ‘present perfect’ (“pretérito perfecto compuesto”) in Spanish as well as related vocabulary such as terms related to diabetes in Spanish.

## Slide 3
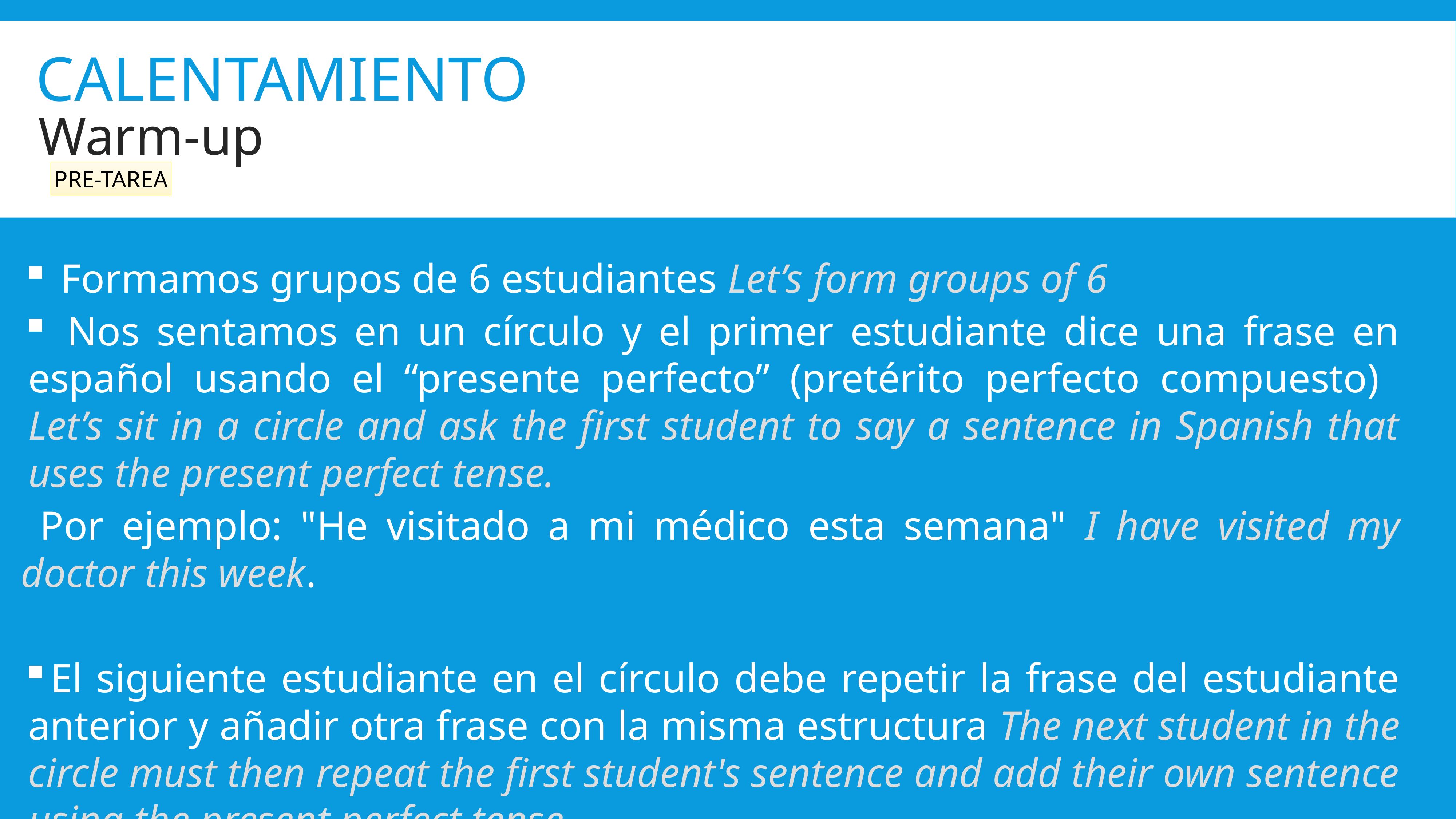

# calentamiento
Warm-up
PRE-TAREA
 Formamos grupos de 6 estudiantes Let’s form groups of 6
 Nos sentamos en un círculo y el primer estudiante dice una frase en español usando el “presente perfecto” (pretérito perfecto compuesto) Let’s sit in a circle and ask the first student to say a sentence in Spanish that uses the present perfect tense.
Por ejemplo: "He visitado a mi médico esta semana" I have visited my doctor this week.
El siguiente estudiante en el círculo debe repetir la frase del estudiante anterior y añadir otra frase con la misma estructura The next student in the circle must then repeat the first student's sentence and add their own sentence using the present perfect tense.
Por ejemplo: "Él me ha recetado un medicamento nuevo" He has prescribed a new medication for me.

## Slide 4
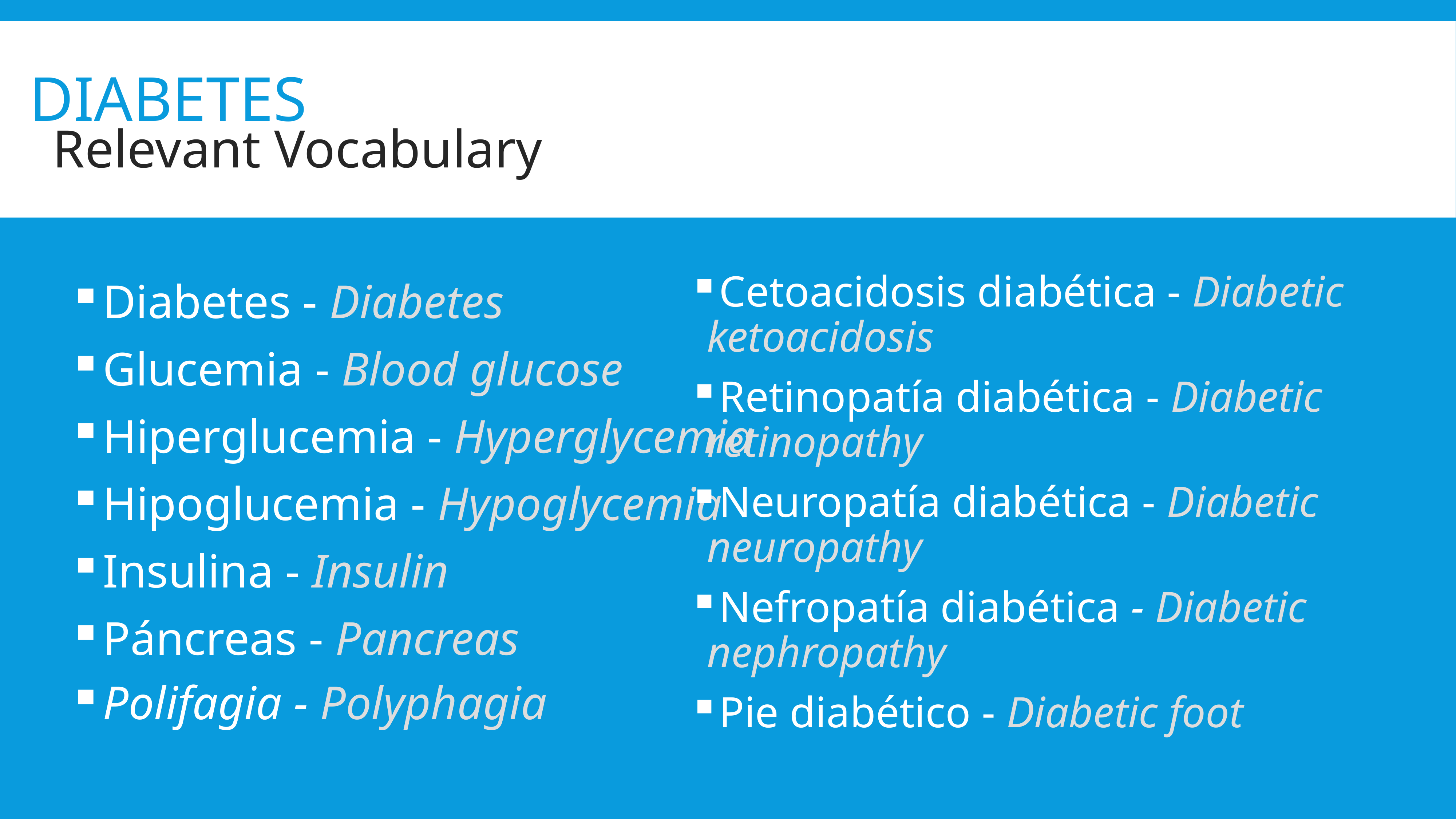

# DIABETES
Relevant Vocabulary
Cetoacidosis diabética - Diabetic ketoacidosis
Retinopatía diabética - Diabetic retinopathy
Neuropatía diabética - Diabetic neuropathy
Nefropatía diabética - Diabetic nephropathy
Pie diabético - Diabetic foot
Diabetes - Diabetes
Glucemia - Blood glucose
Hiperglucemia - Hyperglycemia
Hipoglucemia - Hypoglycemia
Insulina - Insulin
Páncreas - Pancreas
Polifagia - Polyphagia

## Slide 5
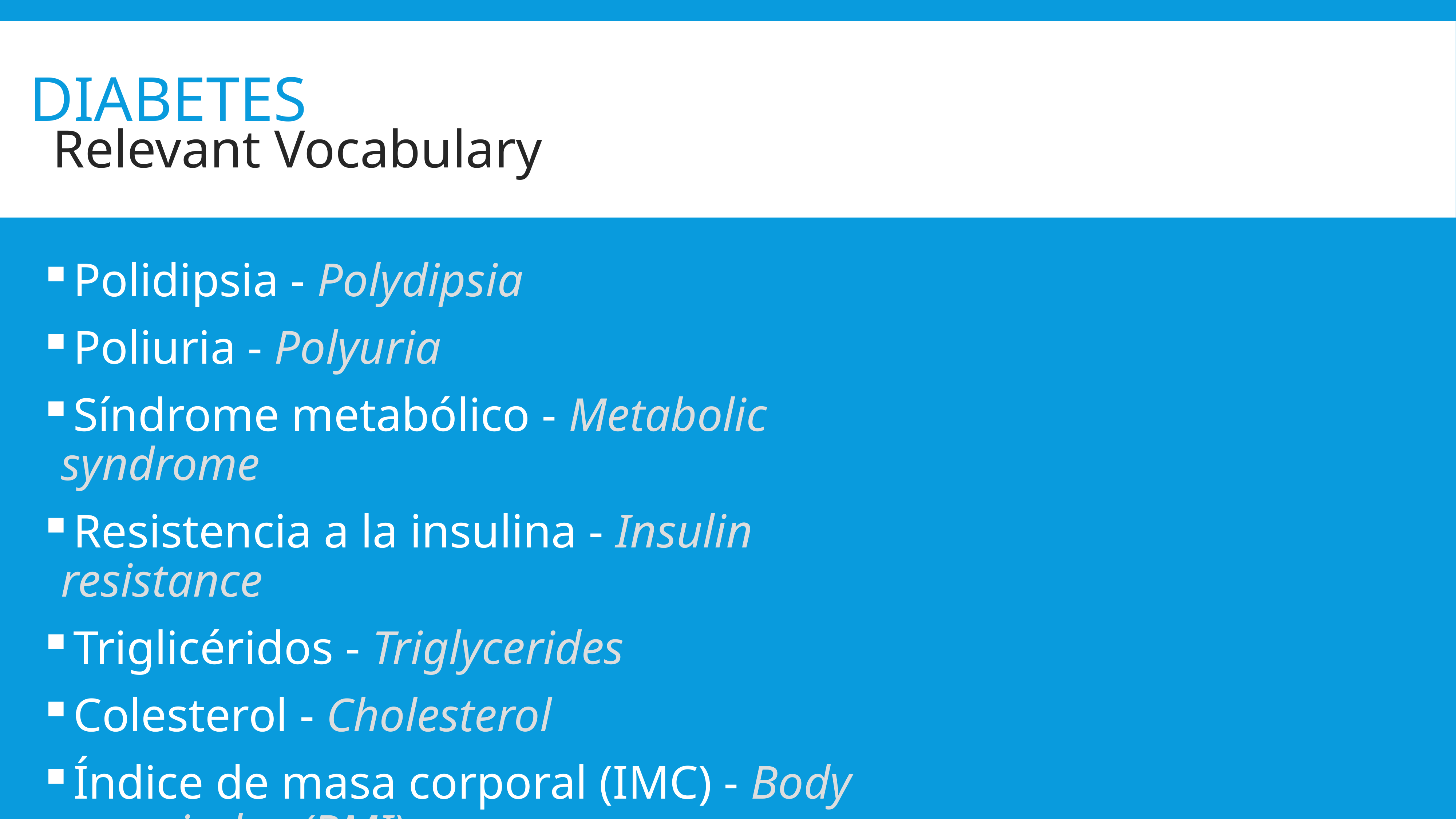

# DIABETES
Relevant Vocabulary
Polidipsia - Polydipsia
Poliuria - Polyuria
Síndrome metabólico - Metabolic syndrome
Resistencia a la insulina - Insulin resistance
Triglicéridos - Triglycerides
Colesterol - Cholesterol
Índice de masa corporal (IMC) - Body mass index (BMI)

## Slide 6
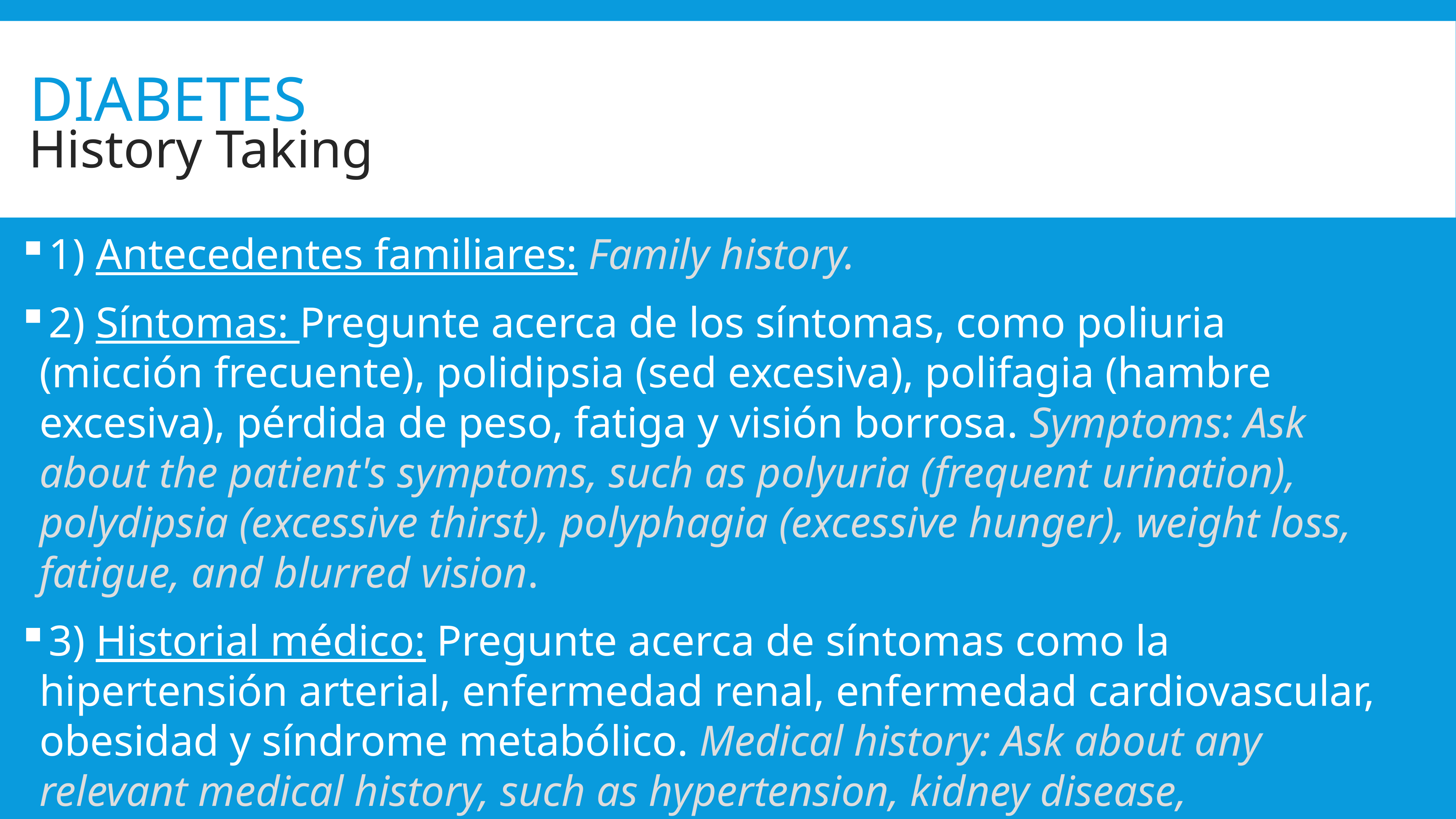

# DIABETES
History Taking
1) Antecedentes familiares: Family history.
2) Síntomas: Pregunte acerca de los síntomas, como poliuria (micción frecuente), polidipsia (sed excesiva), polifagia (hambre excesiva), pérdida de peso, fatiga y visión borrosa. Symptoms: Ask about the patient's symptoms, such as polyuria (frequent urination), polydipsia (excessive thirst), polyphagia (excessive hunger), weight loss, fatigue, and blurred vision.
3) Historial médico: Pregunte acerca de síntomas como la hipertensión arterial, enfermedad renal, enfermedad cardiovascular, obesidad y síndrome metabólico. Medical history: Ask about any relevant medical history, such as hypertension, kidney disease, cardiovascular disease, obesity, and metabolic syndrome.

## Slide 7
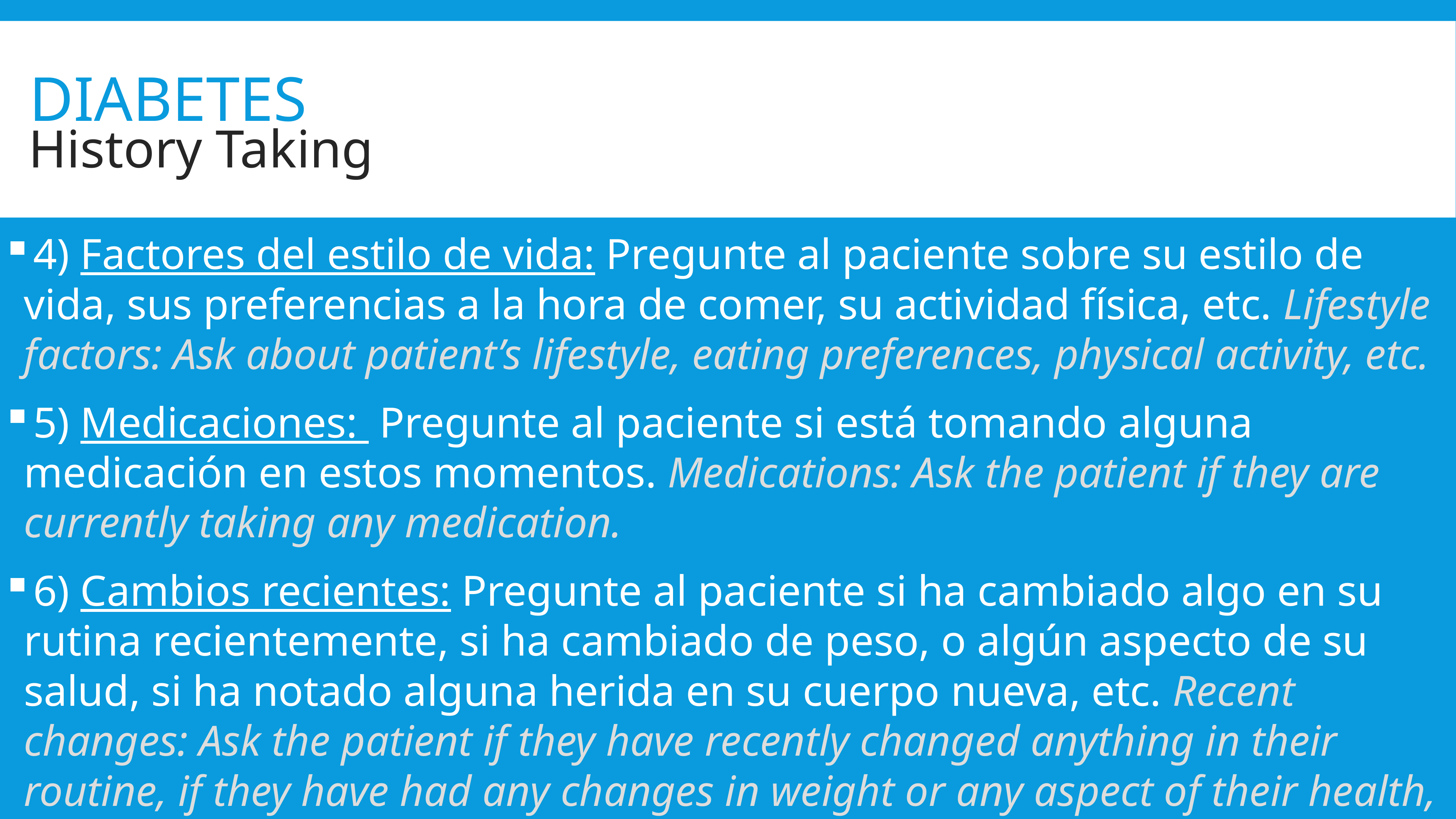

# DIABETES
History Taking
4) Factores del estilo de vida: Pregunte al paciente sobre su estilo de vida, sus preferencias a la hora de comer, su actividad física, etc. Lifestyle factors: Ask about patient’s lifestyle, eating preferences, physical activity, etc.
5) Medicaciones: Pregunte al paciente si está tomando alguna medicación en estos momentos. Medications: Ask the patient if they are currently taking any medication.
6) Cambios recientes: Pregunte al paciente si ha cambiado algo en su rutina recientemente, si ha cambiado de peso, o algún aspecto de su salud, si ha notado alguna herida en su cuerpo nueva, etc. Recent changes: Ask the patient if they have recently changed anything in their routine, if they have had any changes in weight or any aspect of their health, if they have noticed any new wounds on their body, etc.

## Slide 8
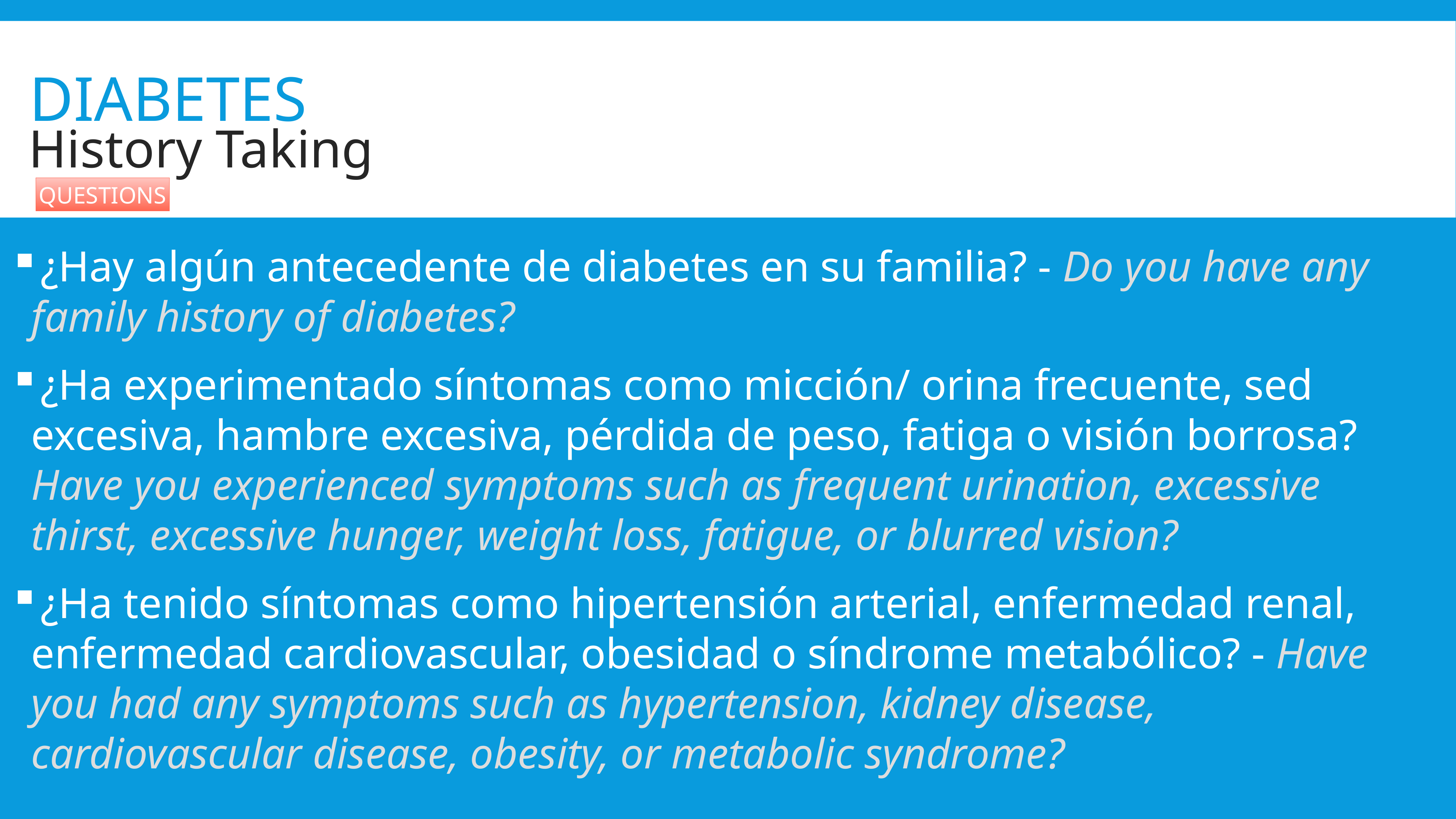

# DIABETES
History Taking
¿Hay algún antecedente de diabetes en su familia? - Do you have any family history of diabetes?
¿Ha experimentado síntomas como micción/ orina frecuente, sed excesiva, hambre excesiva, pérdida de peso, fatiga o visión borrosa? Have you experienced symptoms such as frequent urination, excessive thirst, excessive hunger, weight loss, fatigue, or blurred vision?
¿Ha tenido síntomas como hipertensión arterial, enfermedad renal, enfermedad cardiovascular, obesidad o síndrome metabólico? - Have you had any symptoms such as hypertension, kidney disease, cardiovascular disease, obesity, or metabolic syndrome?
QUESTIONS

## Slide 9
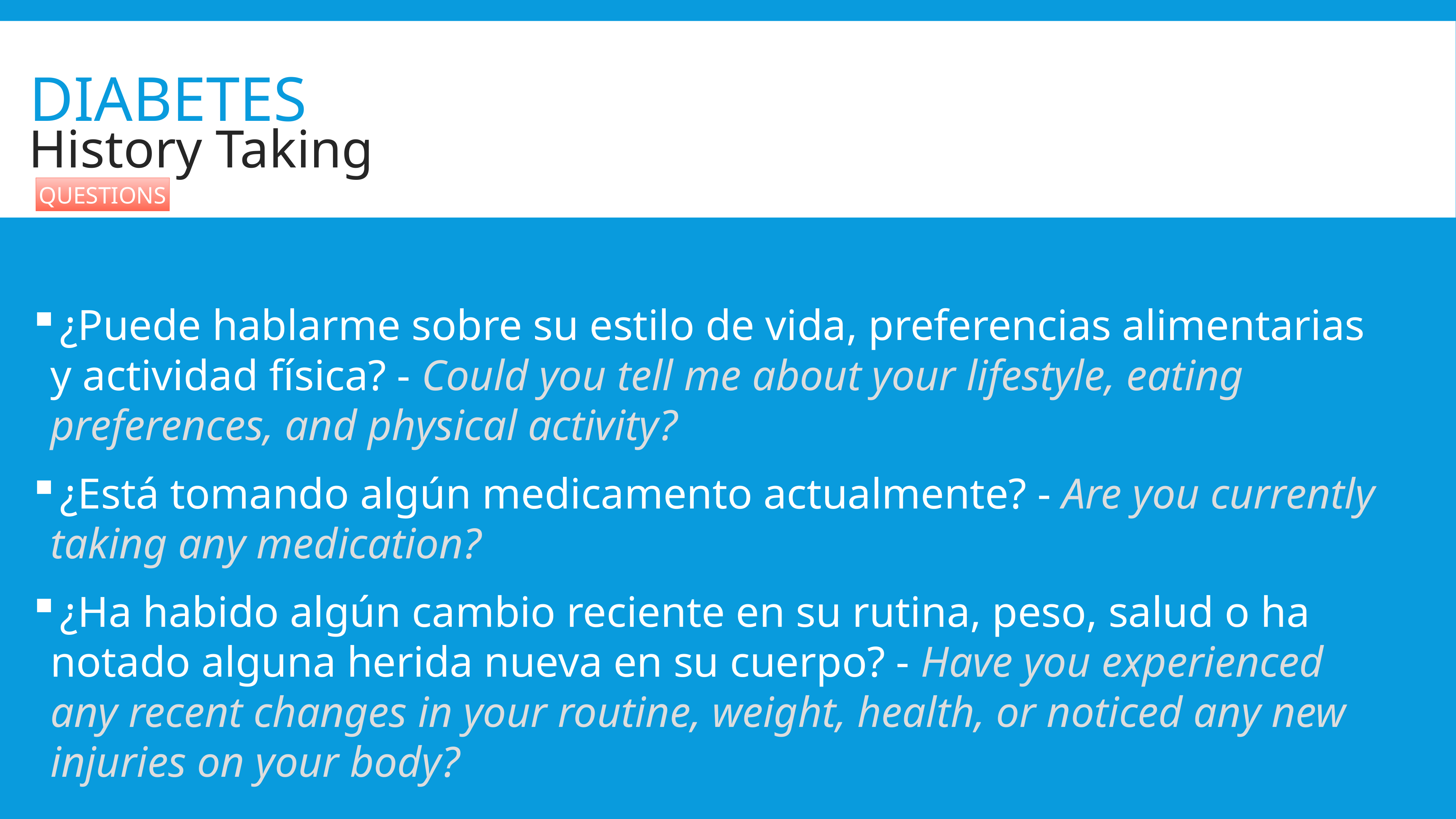

# DIABETES
History Taking
QUESTIONS
¿Puede hablarme sobre su estilo de vida, preferencias alimentarias y actividad física? - Could you tell me about your lifestyle, eating preferences, and physical activity?
¿Está tomando algún medicamento actualmente? - Are you currently taking any medication?
¿Ha habido algún cambio reciente en su rutina, peso, salud o ha notado alguna herida nueva en su cuerpo? - Have you experienced any recent changes in your routine, weight, health, or noticed any new injuries on your body?

## Slide 10
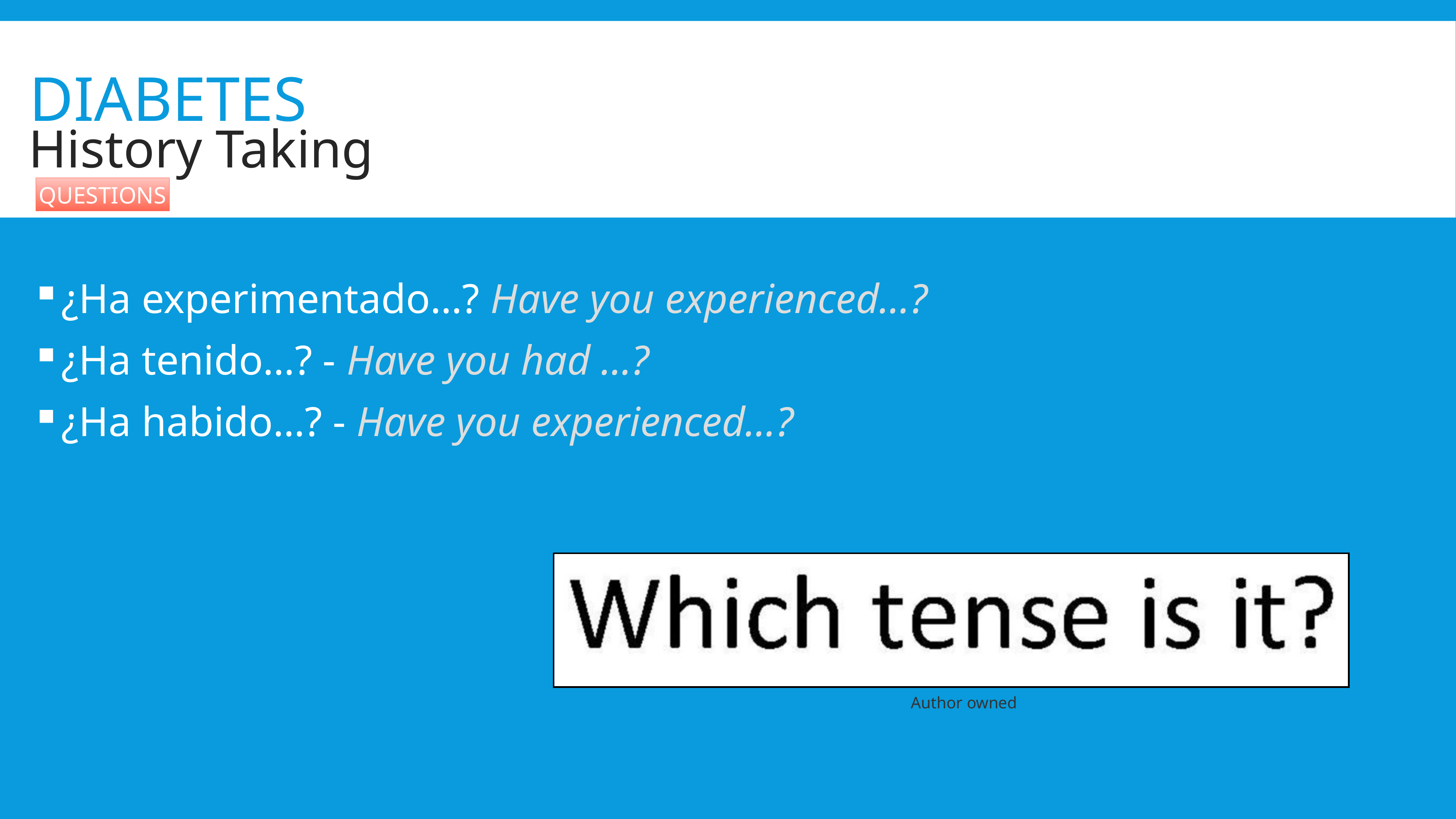

# DIABETES
History Taking
QUESTIONS
¿Ha experimentado…? Have you experienced…?
¿Ha tenido…? - Have you had …?
¿Ha habido…? - Have you experienced…?
Author owned

## Slide 11
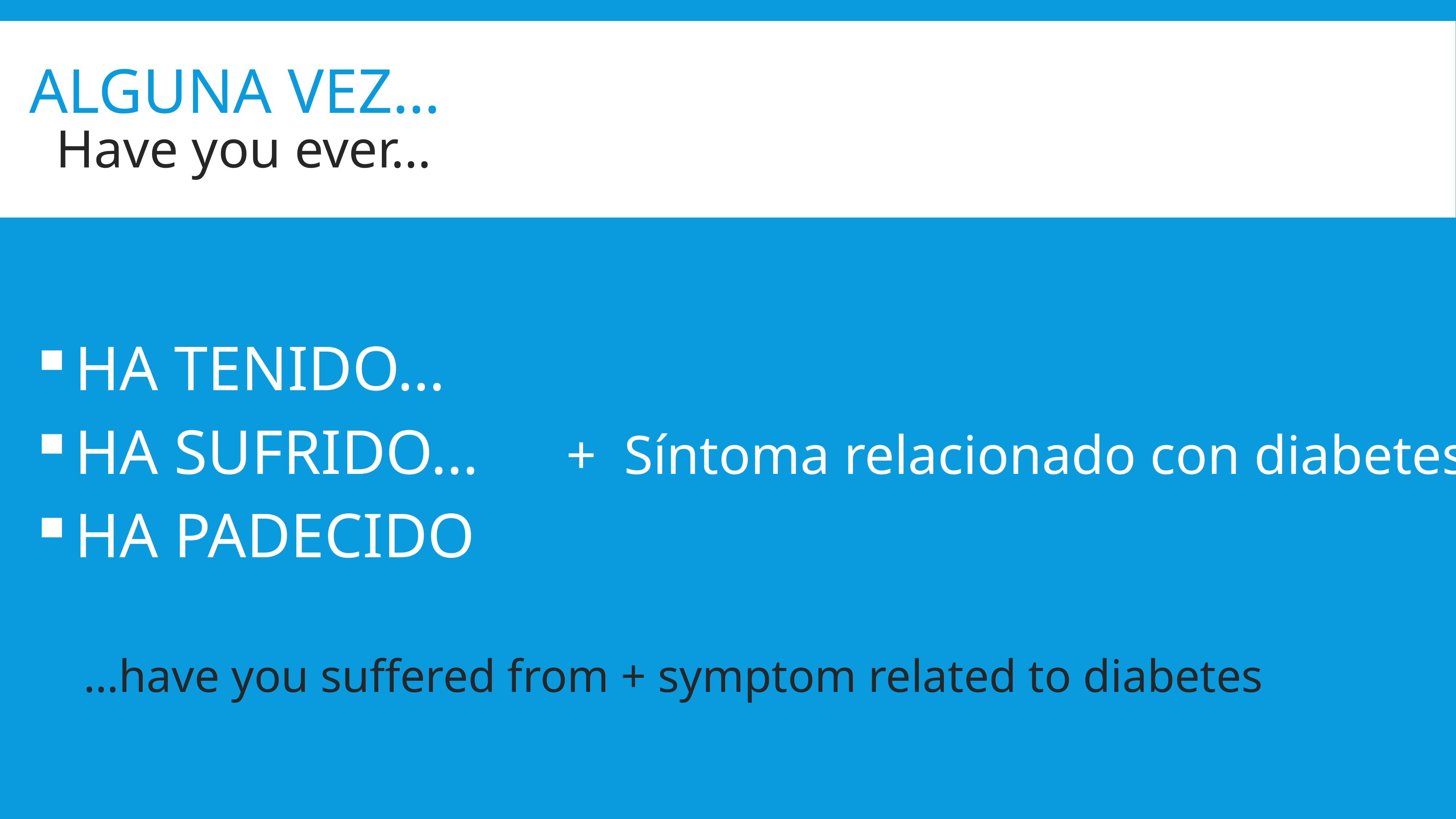

# ALGUNA VEZ…
Have you ever…
HA TENIDO…
HA SUFRIDO…
HA PADECIDO
 + Síntoma relacionado con diabetes
…have you suffered from + symptom related to diabetes

## Slide 12
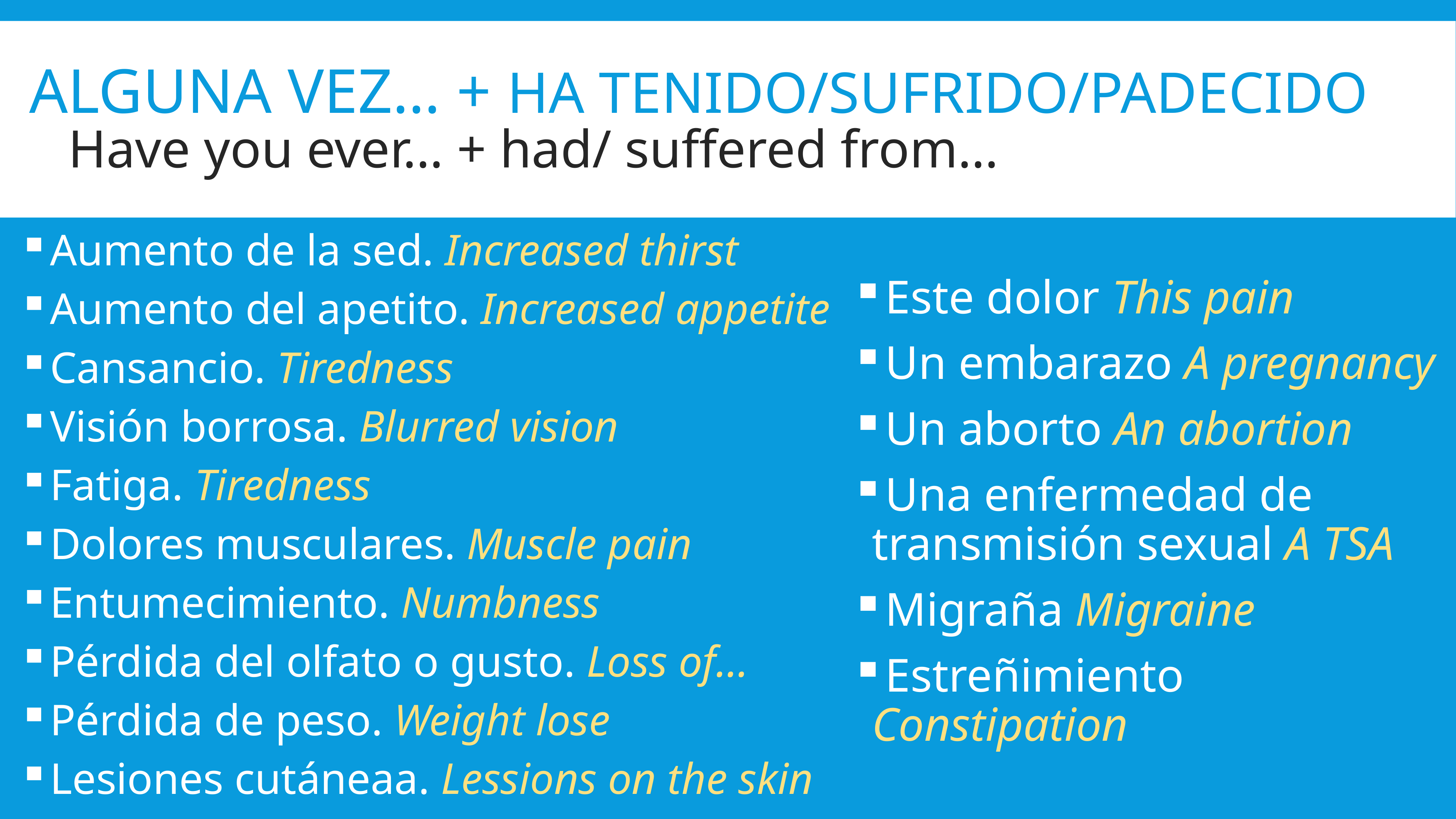

# ALGUNA VEZ… + HA tenido/sufrido/PADECIDO
Have you ever… + had/ suffered from…
Aumento de la sed. Increased thirst
Aumento del apetito. Increased appetite
Cansancio. Tiredness
Visión borrosa. Blurred vision
Fatiga. Tiredness
Dolores musculares. Muscle pain
Entumecimiento. Numbness
Pérdida del olfato o gusto. Loss of…
Pérdida de peso. Weight lose
Lesiones cutáneaa. Lessions on the skin
Este dolor This pain
Un embarazo A pregnancy
Un aborto An abortion
Una enfermedad de transmisión sexual A TSA
Migraña Migraine
Estreñimiento Constipation

## Slide 13
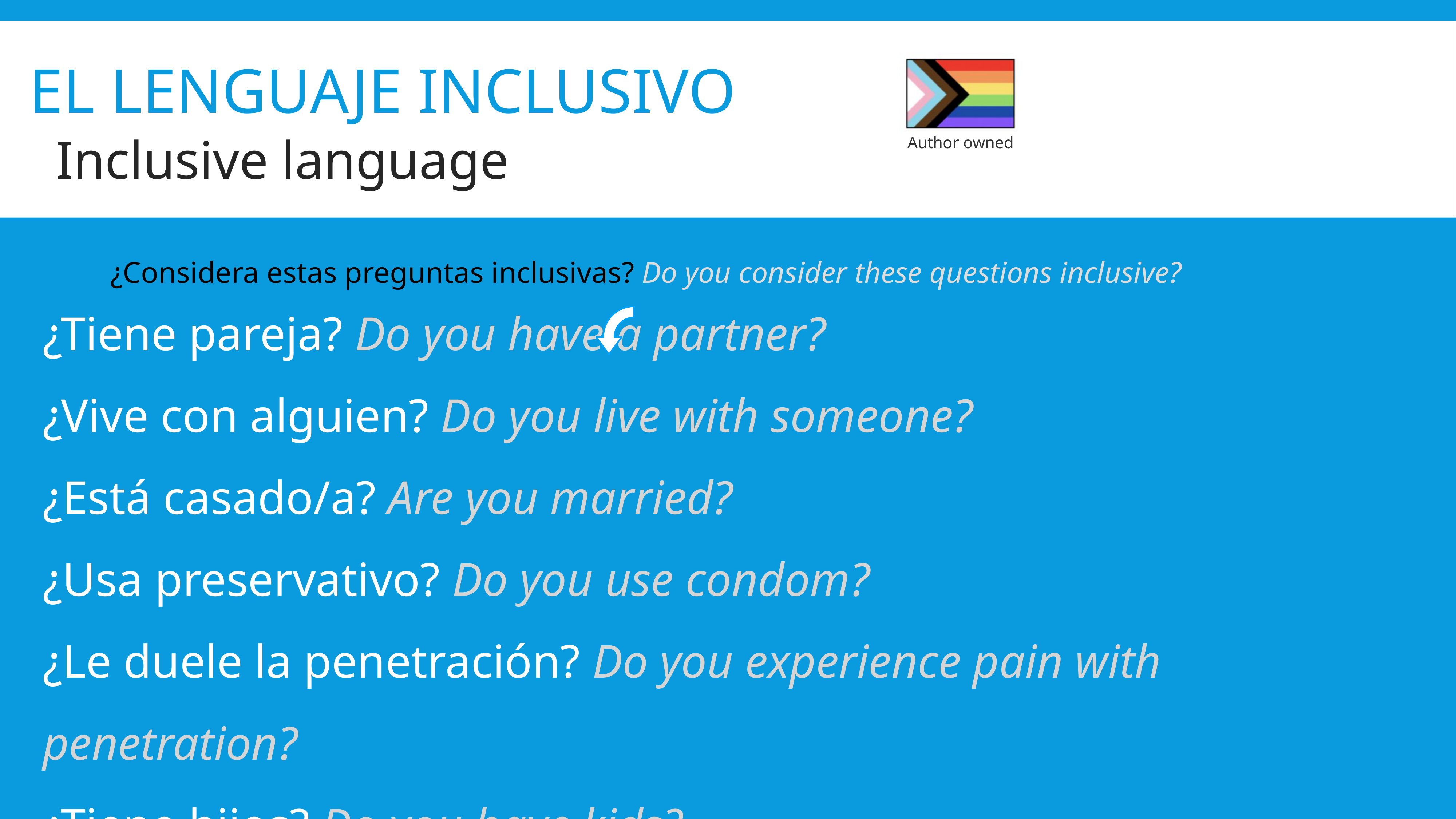

# El lenguaje inclusivo
Inclusive language
Author owned
¿Considera estas preguntas inclusivas? Do you consider these questions inclusive?
¿Tiene pareja? Do you have a partner?
¿Vive con alguien? Do you live with someone?
¿Está casado/a? Are you married?
¿Usa preservativo? Do you use condom?
¿Le duele la penetración? Do you experience pain with penetration?
¿Tiene hijos? Do you have kids?

## Slide 14
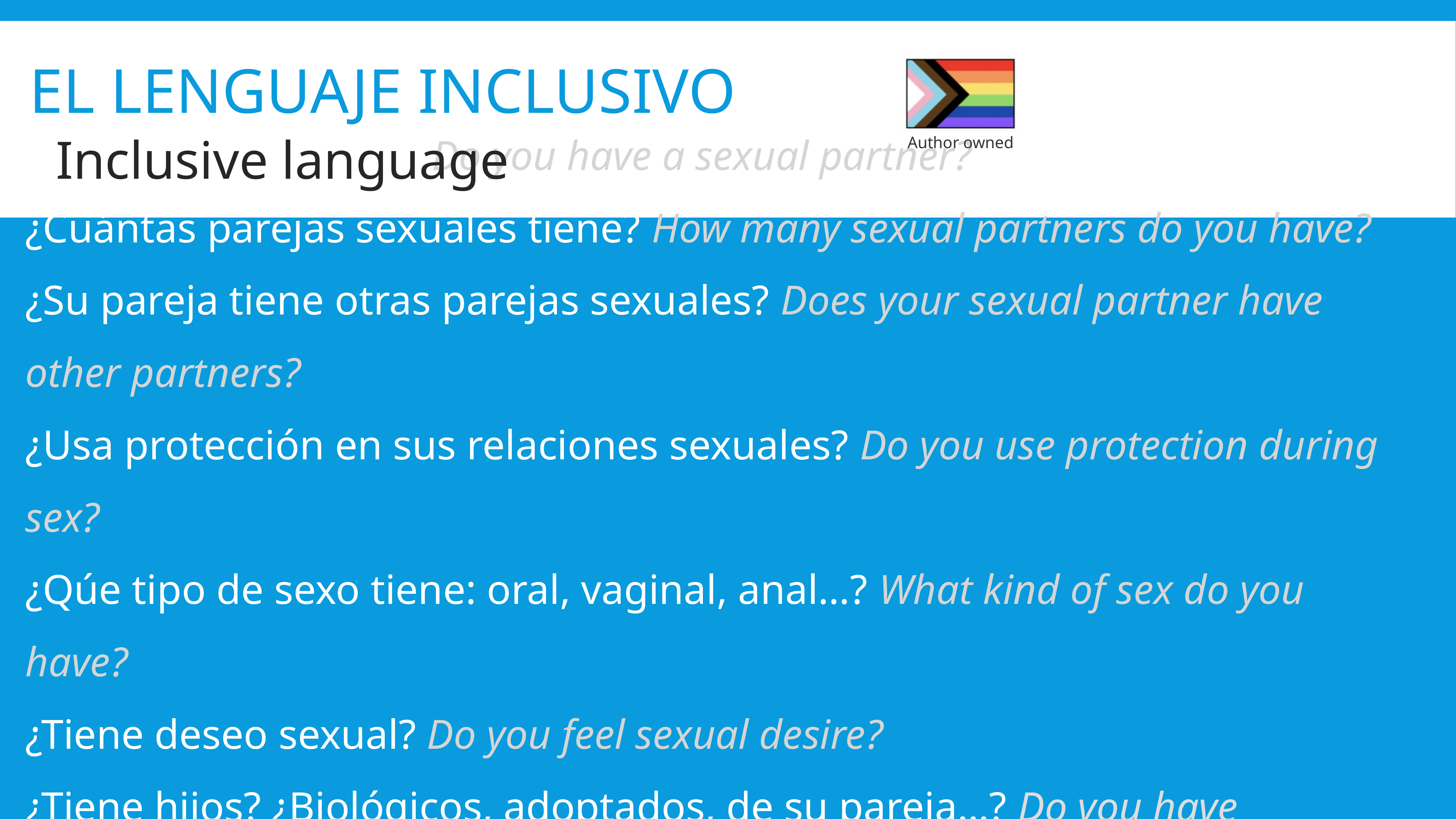

# El lenguaje inclusivo
Inclusive language
Author owned
¿Tiene pareja sexual? Do you have a sexual partner?
¿Cuántas parejas sexuales tiene? How many sexual partners do you have?
¿Su pareja tiene otras parejas sexuales? Does your sexual partner have other partners?
¿Usa protección en sus relaciones sexuales? Do you use protection during sex?
¿Qúe tipo de sexo tiene: oral, vaginal, anal…? What kind of sex do you have?
¿Tiene deseo sexual? Do you feel sexual desire?
¿Tiene hijos? ¿Biológicos, adoptados, de su pareja…? Do you have children?
Biological, adopted, from your partner...?

## Slide 15
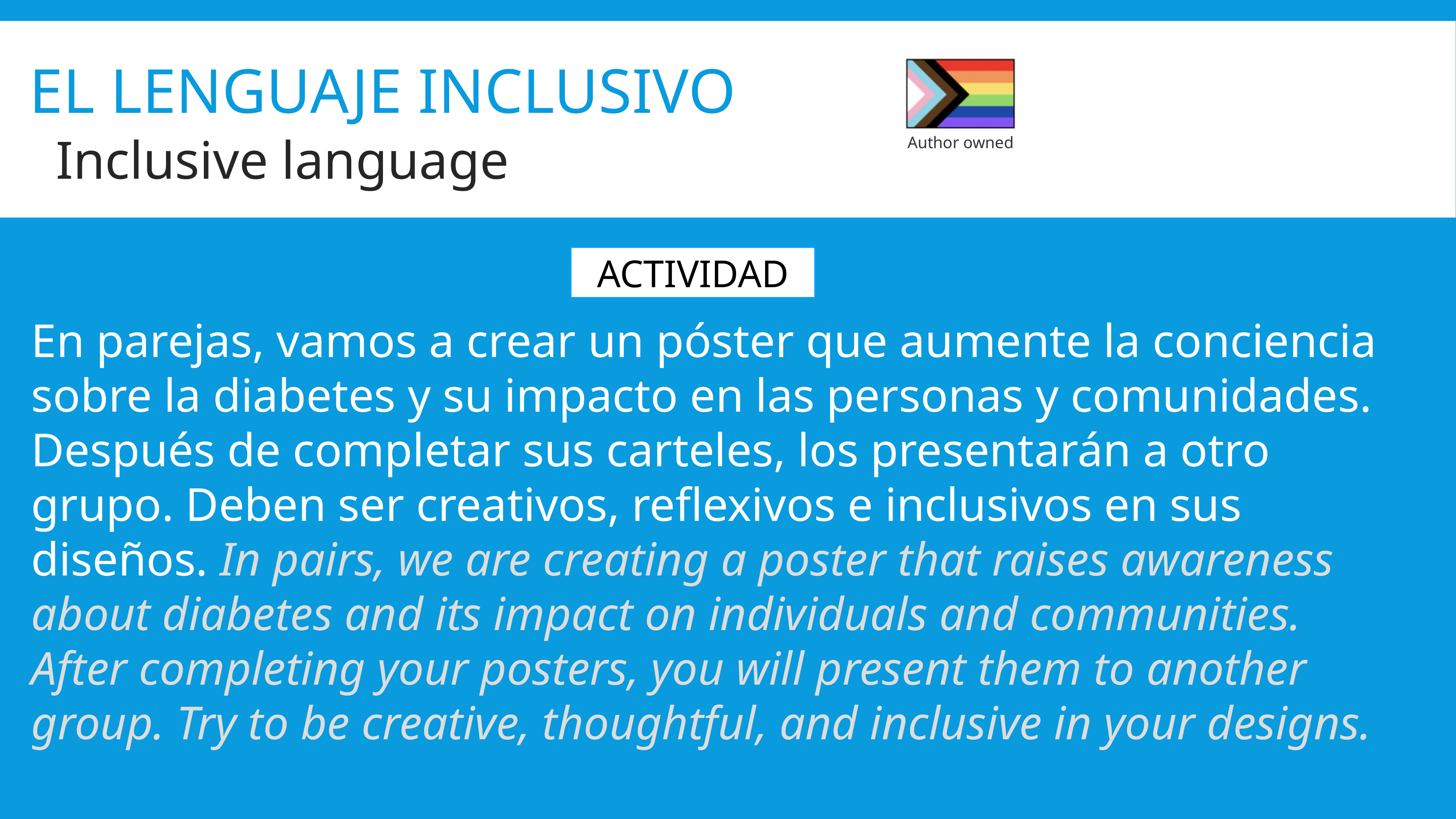

# El lenguaje inclusivo
Inclusive language
Author owned
ACTIVIDAD
En parejas, vamos a crear un póster que aumente la conciencia sobre la diabetes y su impacto en las personas y comunidades. Después de completar sus carteles, los presentarán a otro grupo. Deben ser creativos, reflexivos e inclusivos en sus diseños. In pairs, we are creating a poster that raises awareness about diabetes and its impact on individuals and communities. After completing your posters, you will present them to another group. Try to be creative, thoughtful, and inclusive in your designs.

## Slide 16
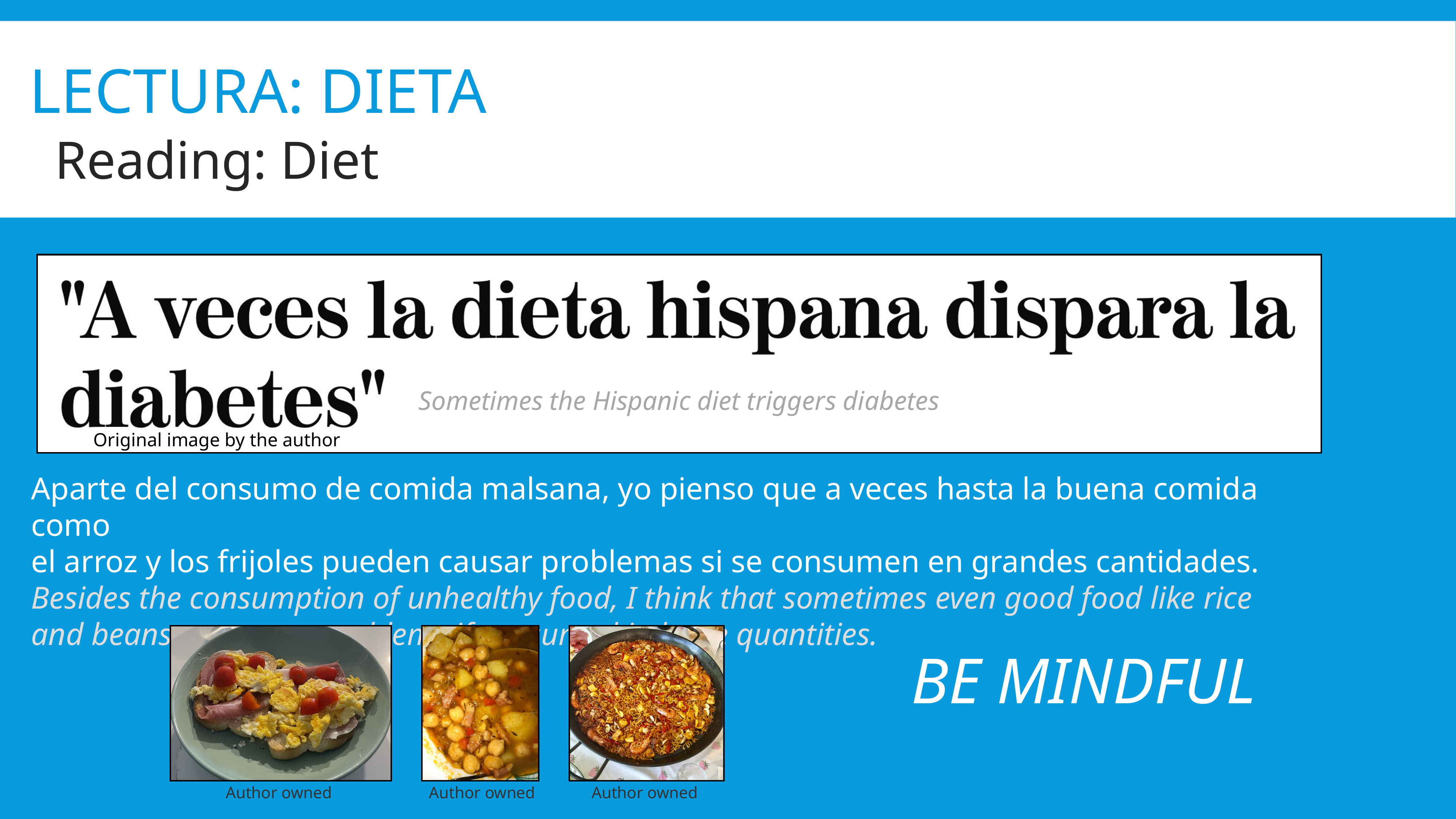

# Lectura: dieta
Reading: Diet
Sometimes the Hispanic diet triggers diabetes
Original image by the author
Aparte del consumo de comida malsana, yo pienso que a veces hasta la buena comida como
el arroz y los frijoles pueden causar problemas si se consumen en grandes cantidades. Besides the consumption of unhealthy food, I think that sometimes even good food like rice and beans can cause problems if consumed in large quantities.
BE MINDFUL
Author owned
Author owned
Author owned

## Slide 17
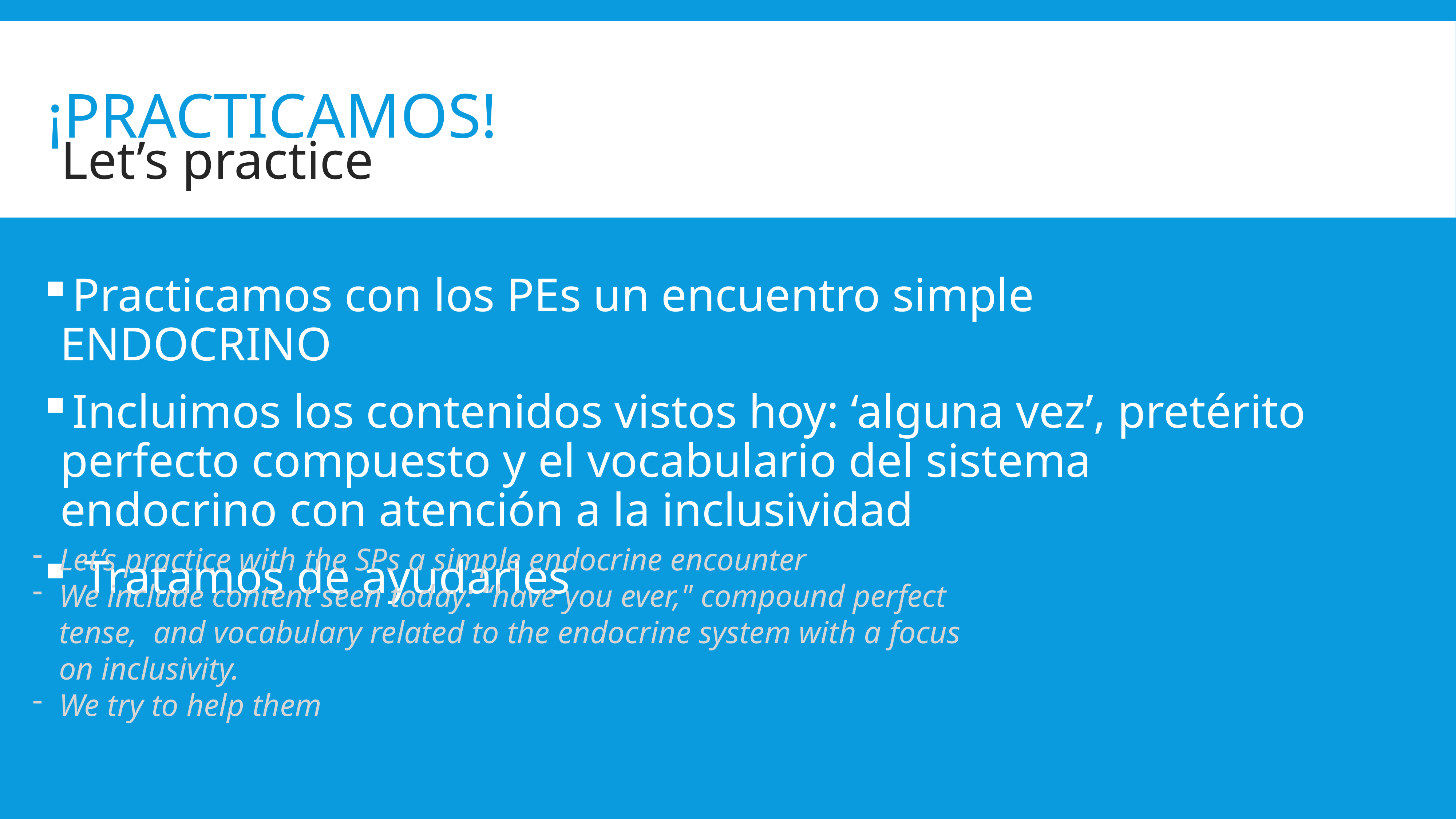

# ¡Practicamos!
Let’s practice
Practicamos con los PEs un encuentro simple ENDOCRINO
Incluimos los contenidos vistos hoy: ‘alguna vez’, pretérito perfecto compuesto y el vocabulario del sistema endocrino con atención a la inclusividad
 Tratamos de ayudarles
Let’s practice with the SPs a simple endocrine encounter
We include content seen today: “have you ever," compound perfect tense, and vocabulary related to the endocrine system with a focus on inclusivity.
We try to help them

## Slide 18
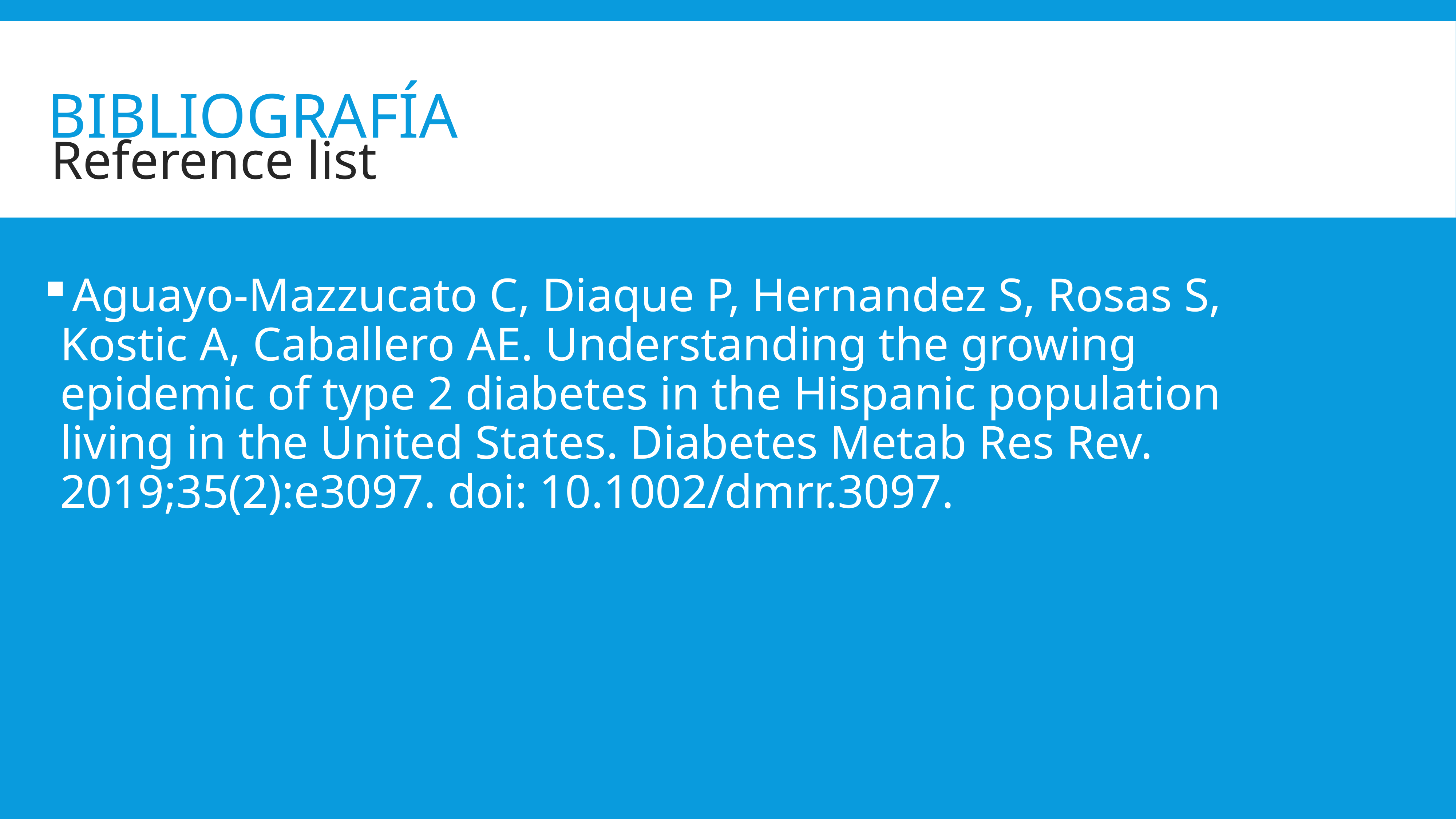

# BIBLIOGRAFía
Reference list
Aguayo-Mazzucato C, Diaque P, Hernandez S, Rosas S, Kostic A, Caballero AE. Understanding the growing epidemic of type 2 diabetes in the Hispanic population living in the United States. Diabetes Metab Res Rev. 2019;35(2):e3097. doi: 10.1002/dmrr.3097.
