# Supplementary material for: Integrating Gender-Affirming Care in a Medical Spanish Endocrine System Curriculum
Source: MedEdPORTAL. 2024 Oct 23;20:11456. doi: 10.15766/mep_2374-8265.11456 (PMC11496385; doi:10.15766/mep_2374-8265.11456)
Supplement: Supplementary file 1 — Facilitator Guide.docxLesson 1 Presentation.pptxLesson 2 Presentation.pptxLesson 3 Presentation.pptxLesson 1 Clinical Endocrine Checklist.docxLesson 2 Clinical Endocrine Checklist.docxLesson 3 Clinical Endocrine Checklist.docxLesson 1 SP Case.docxLesson 2 SP Case.docxLesson 3 SP Case.docxPre-Post Confidence Survey.docxPre-Post Spanish Endocrine Test.docxOSCE SP Diabetic Case.docxOSCE Door Note.docxOSCE Clinical Checklist Diabetic Encounter.docxOSCE Language Rubric for Diabetic Encounter.docx [file mep_2374-8265.11456-s001.zip › C. Lesson 2 Presentation.pptx]

## Slide 1
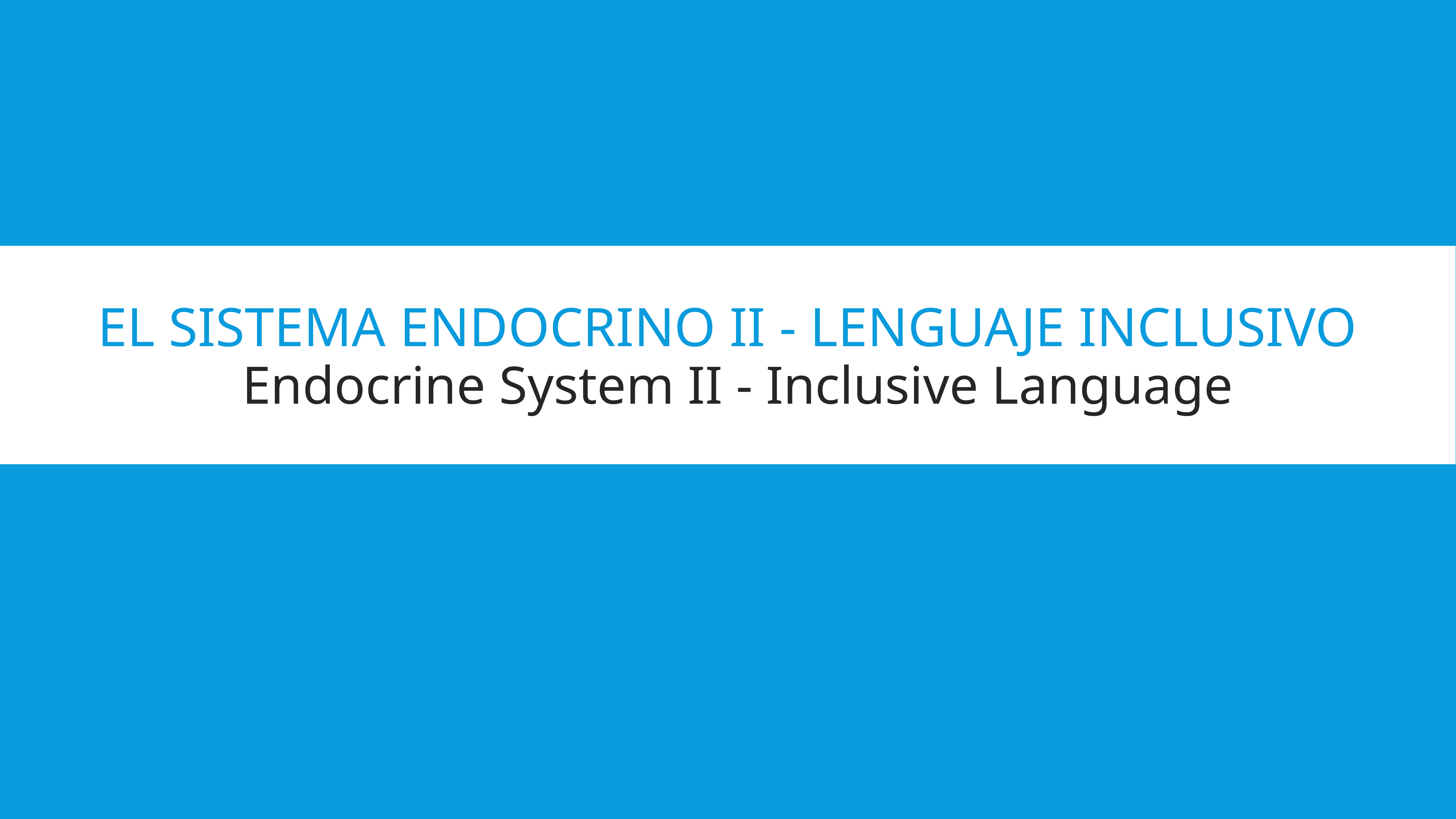

# El sistema endocrino iI - lenguaje inclusivo
Endocrine System II - Inclusive Language

## Slide 2
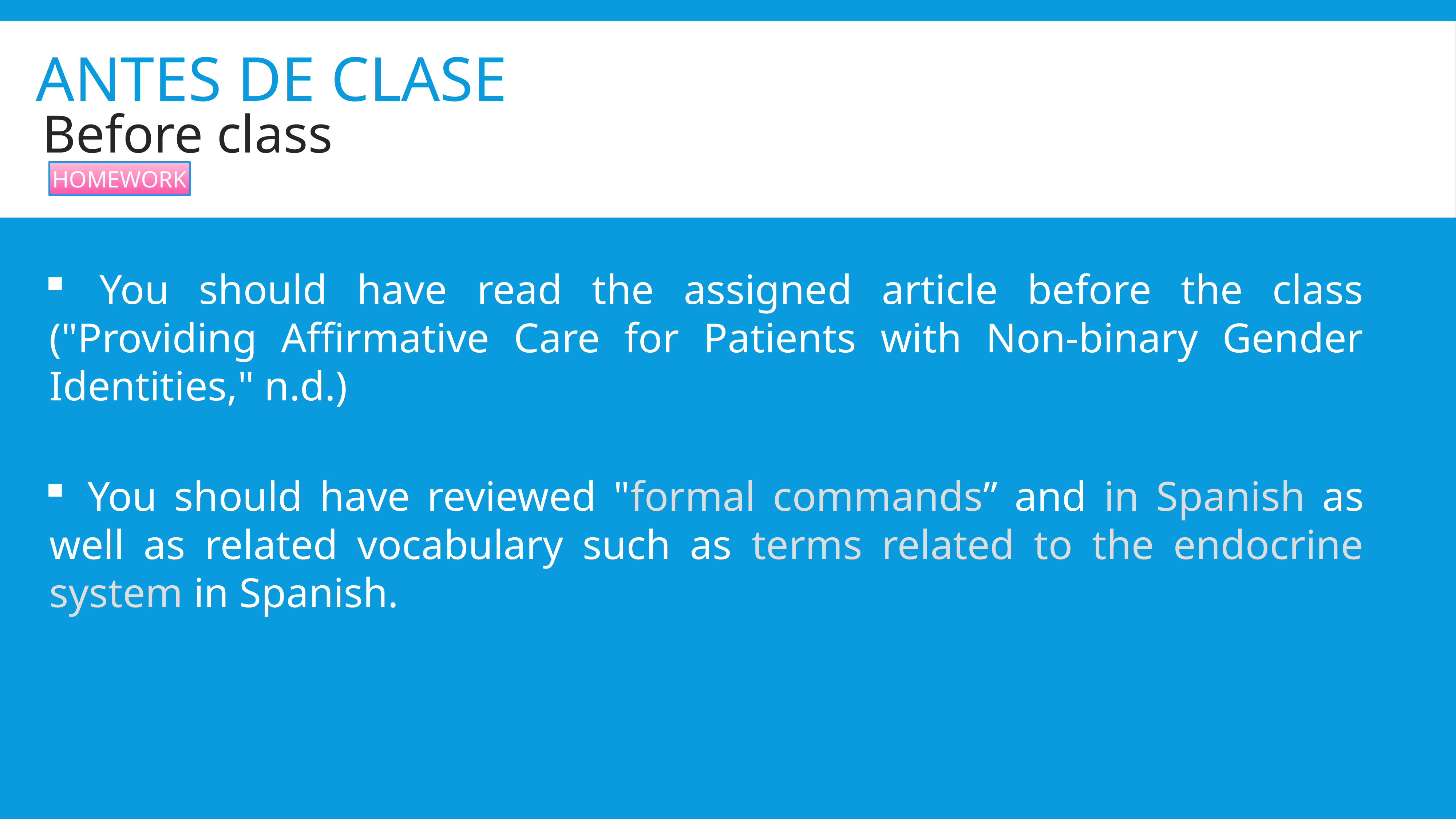

# Antes de clase
Before class
HOMEWORK
 You should have read the assigned article before the class ("Providing Affirmative Care for Patients with Non-binary Gender Identities," n.d.)
 You should have reviewed "formal commands” and in Spanish as well as related vocabulary such as terms related to the endocrine system in Spanish.

## Slide 3
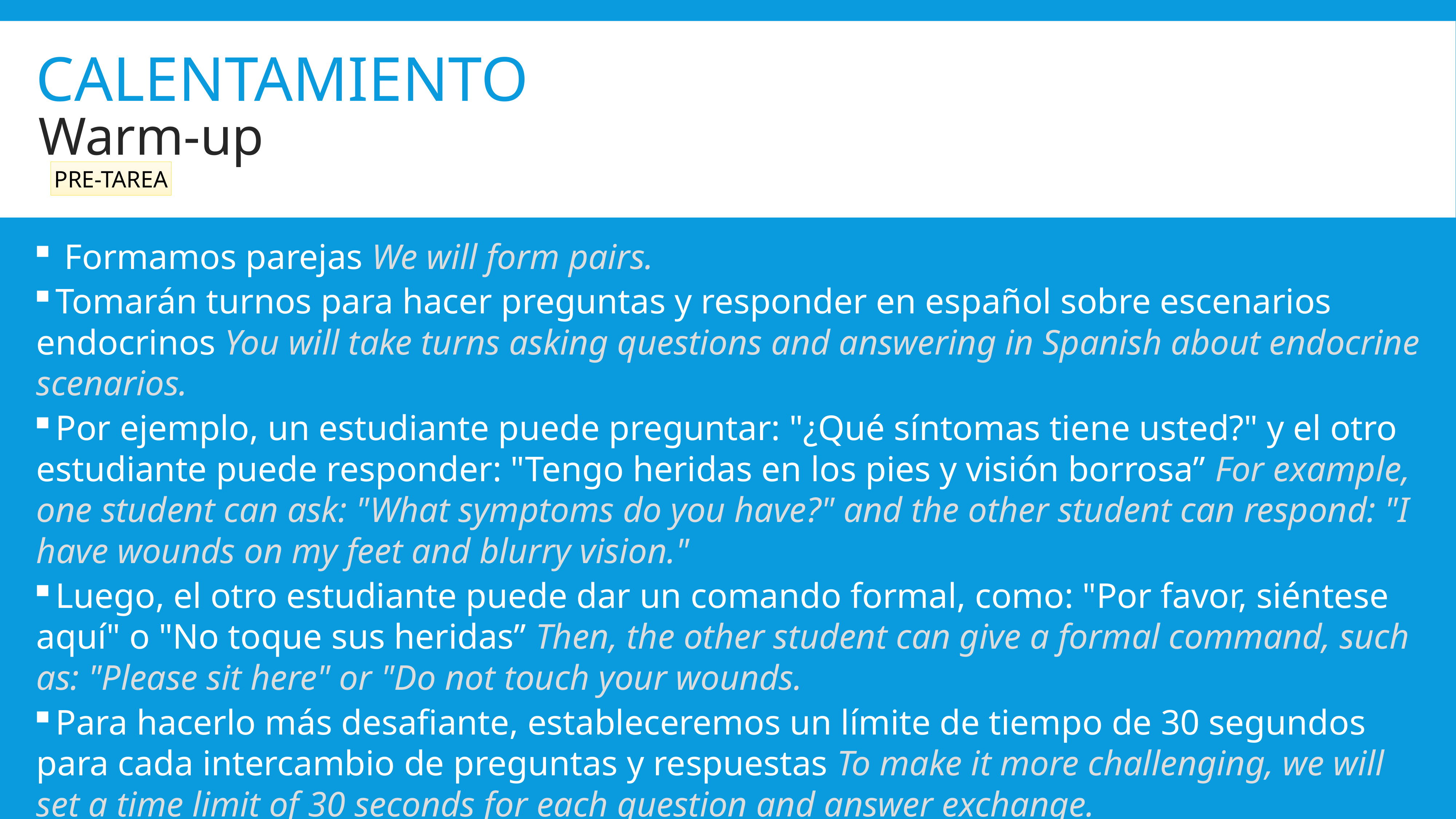

# calentamiento
Warm-up
PRE-TAREA
 Formamos parejas We will form pairs.
Tomarán turnos para hacer preguntas y responder en español sobre escenarios endocrinos You will take turns asking questions and answering in Spanish about endocrine scenarios.
Por ejemplo, un estudiante puede preguntar: "¿Qué síntomas tiene usted?" y el otro estudiante puede responder: "Tengo heridas en los pies y visión borrosa” For example, one student can ask: "What symptoms do you have?" and the other student can respond: "I have wounds on my feet and blurry vision."
Luego, el otro estudiante puede dar un comando formal, como: "Por favor, siéntese aquí" o "No toque sus heridas” Then, the other student can give a formal command, such as: "Please sit here" or "Do not touch your wounds.
Para hacerlo más desafiante, estableceremos un límite de tiempo de 30 segundos para cada intercambio de preguntas y respuestas To make it more challenging, we will set a time limit of 30 seconds for each question and answer exchange.

## Slide 4
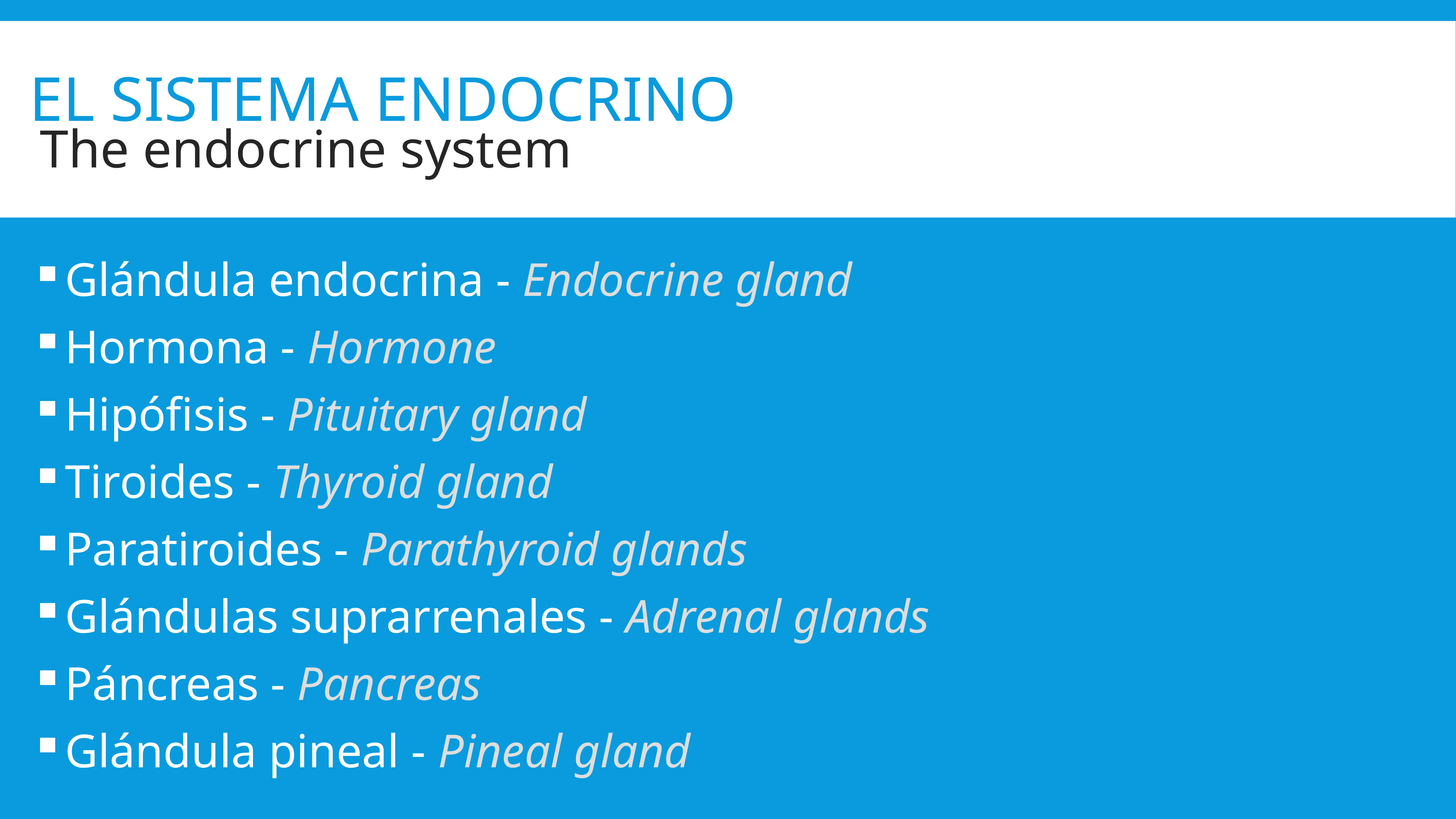

# EL SISTEMA ENDOCRINO
The endocrine system
Glándula endocrina - Endocrine gland
Hormona - Hormone
Hipófisis - Pituitary gland
Tiroides - Thyroid gland
Paratiroides - Parathyroid glands
Glándulas suprarrenales - Adrenal glands
Páncreas - Pancreas
Glándula pineal - Pineal gland

## Slide 5
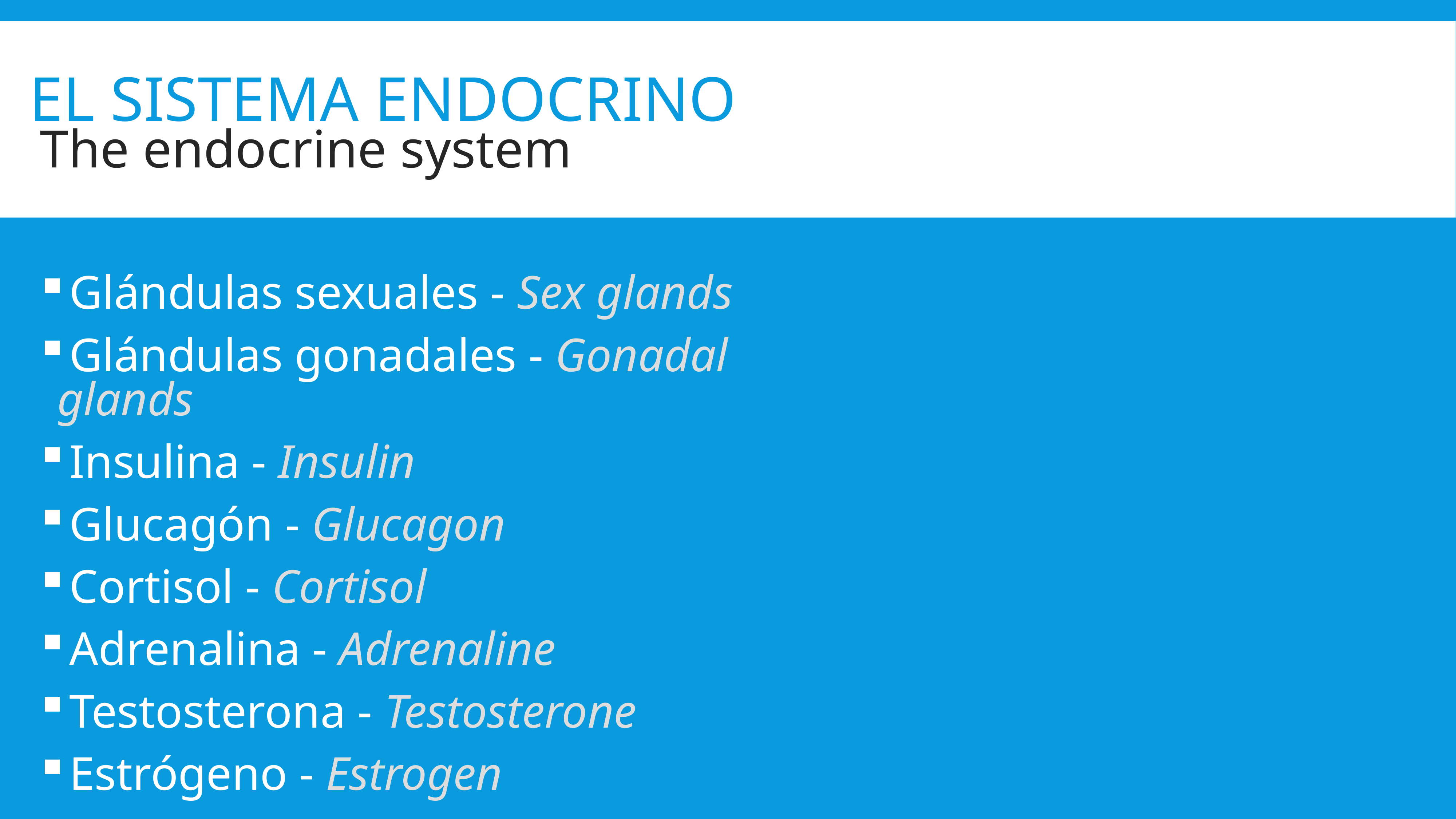

# EL SISTEMA ENDOCRINO
The endocrine system
Glándulas sexuales - Sex glands
Glándulas gonadales - Gonadal glands
Insulina - Insulin
Glucagón - Glucagon
Cortisol - Cortisol
Adrenalina - Adrenaline
Testosterona - Testosterone
Estrógeno - Estrogen

## Slide 6
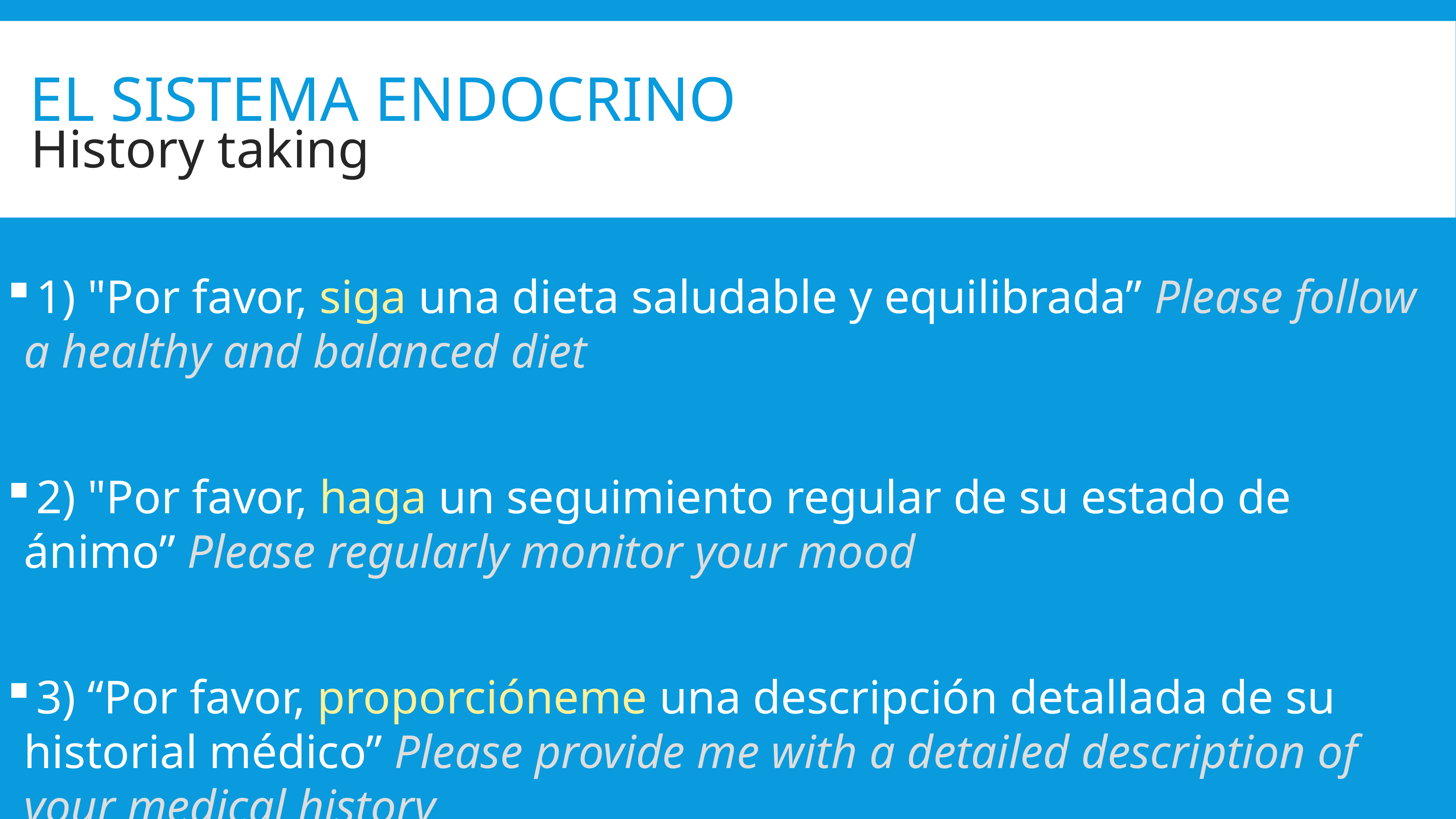

# EL SISTEMA ENDOCRINO
History taking
1) "Por favor, siga una dieta saludable y equilibrada” Please follow a healthy and balanced diet
2) "Por favor, haga un seguimiento regular de su estado de ánimo” Please regularly monitor your mood
3) “Por favor, proporcióneme una descripción detallada de su historial médico” Please provide me with a detailed description of your medical history

## Slide 7
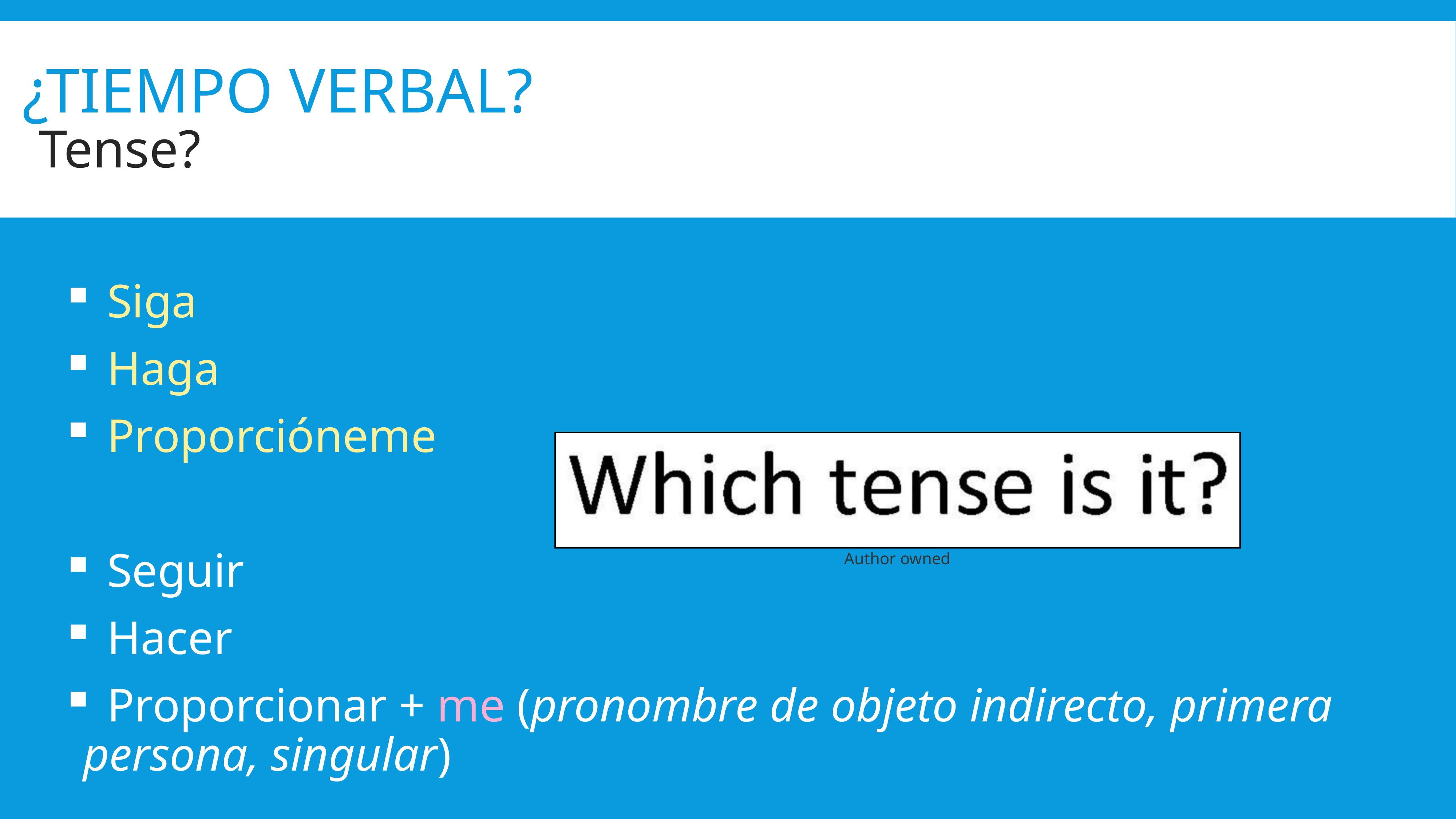

# ¿Tiempo verbal?
Tense?
 Siga
 Haga
 Proporcióneme
 Seguir
 Hacer
 Proporcionar + me (pronombre de objeto indirecto, primera persona, singular)
Author owned

## Slide 8
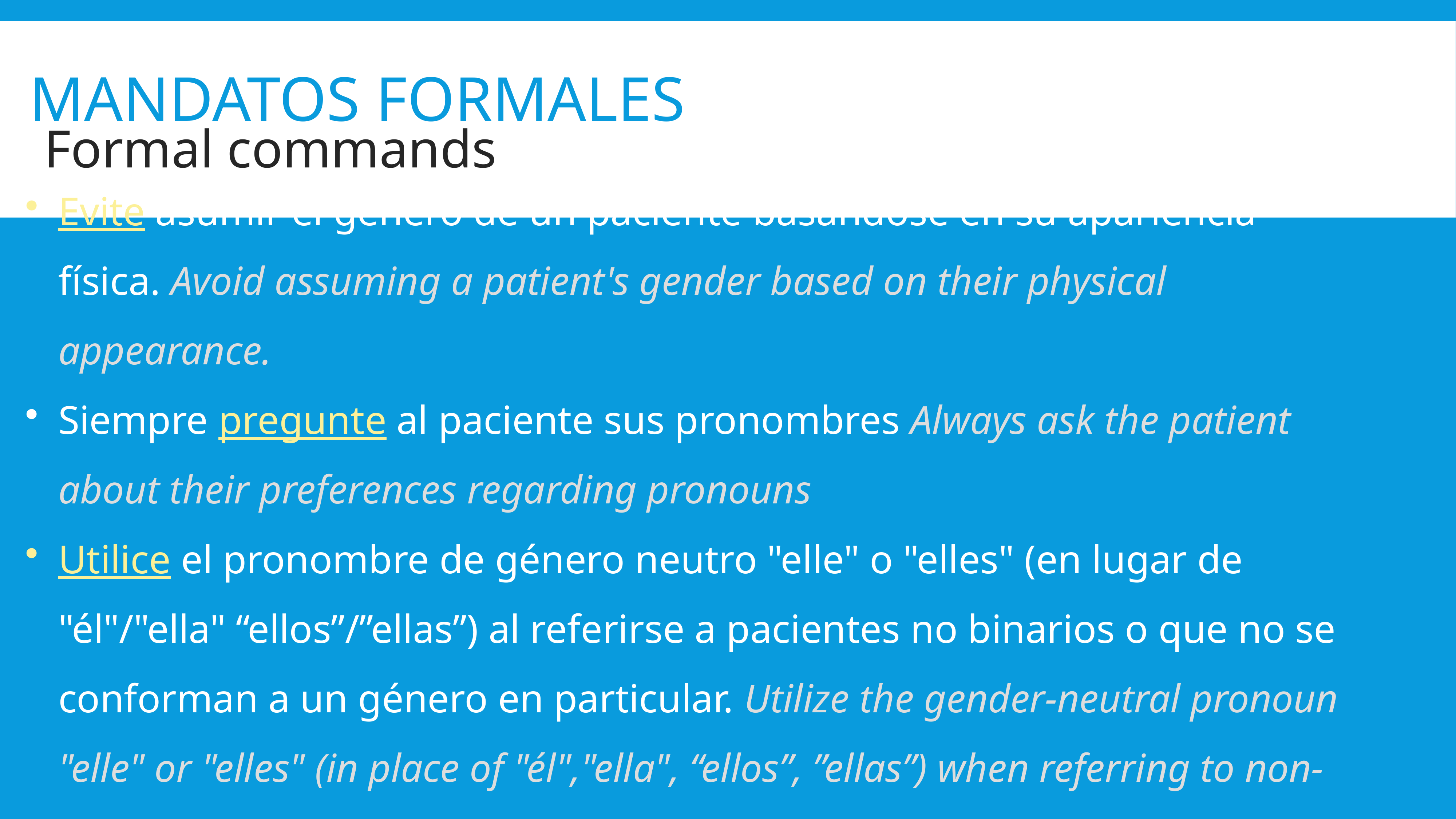

# Mandatos formales
Formal commands
Evite asumir el género de un paciente basándose en su apariencia física. Avoid assuming a patient's gender based on their physical appearance.
Siempre pregunte al paciente sus pronombres Always ask the patient about their preferences regarding pronouns
Utilice el pronombre de género neutro "elle" o "elles" (en lugar de "él"/"ella" “ellos”/”ellas”) al referirse a pacientes no binarios o que no se conforman a un género en particular. Utilize the gender-neutral pronoun "elle" or "elles" (in place of "él","ella", “ellos”, ”ellas”) when referring to non-binary or gender non-conforming patients

## Slide 9
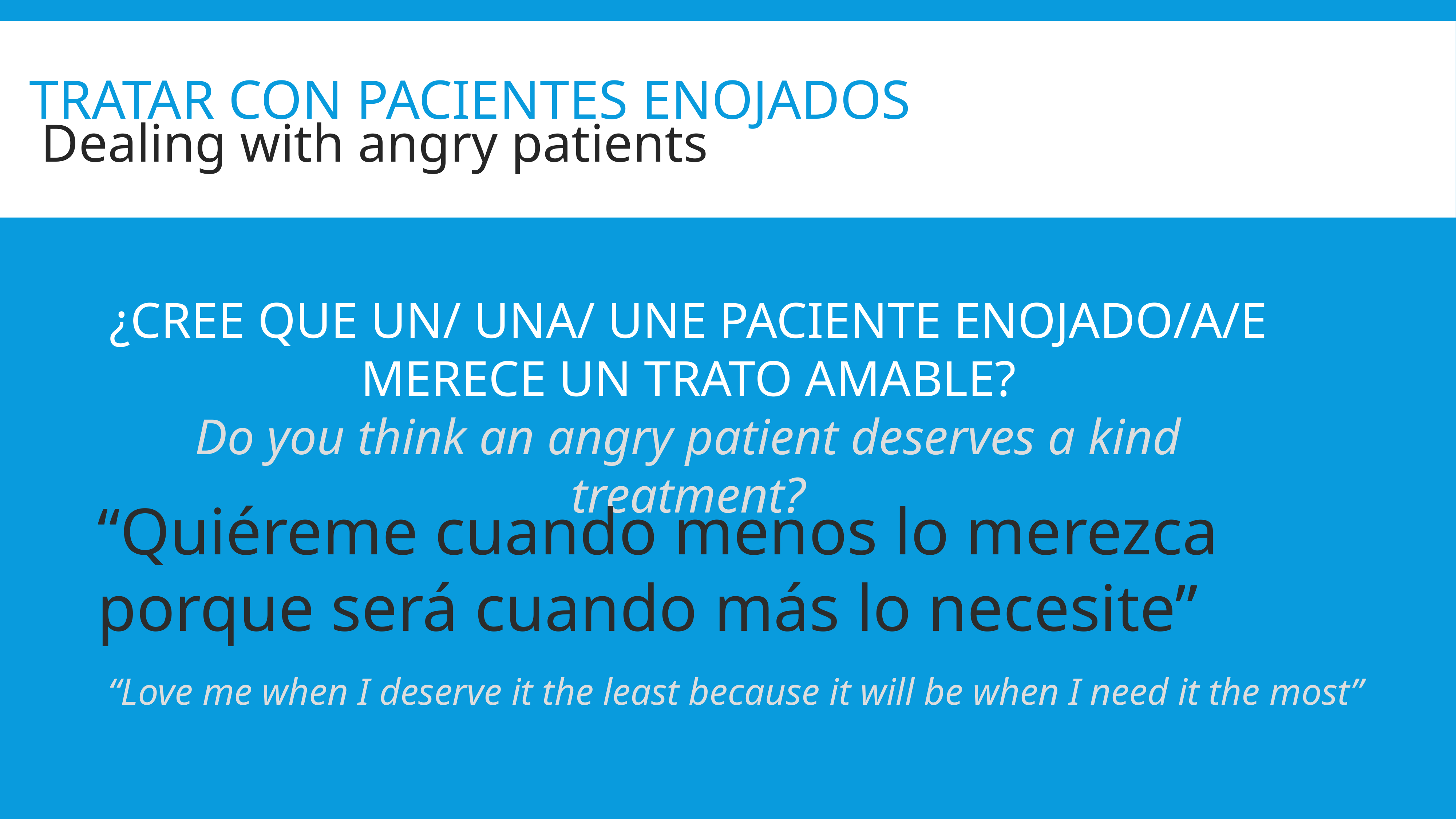

# Tratar con pacientes enojados
Dealing with angry patients
¿CREE QUE UN/ UNA/ UNE PACIENTE ENOJADO/A/E MERECE UN TRATO AMABLE?
Do you think an angry patient deserves a kind treatment?
“Quiéreme cuando menos lo merezca porque será cuando más lo necesite”
“Love me when I deserve it the least because it will be when I need it the most”

## Slide 10
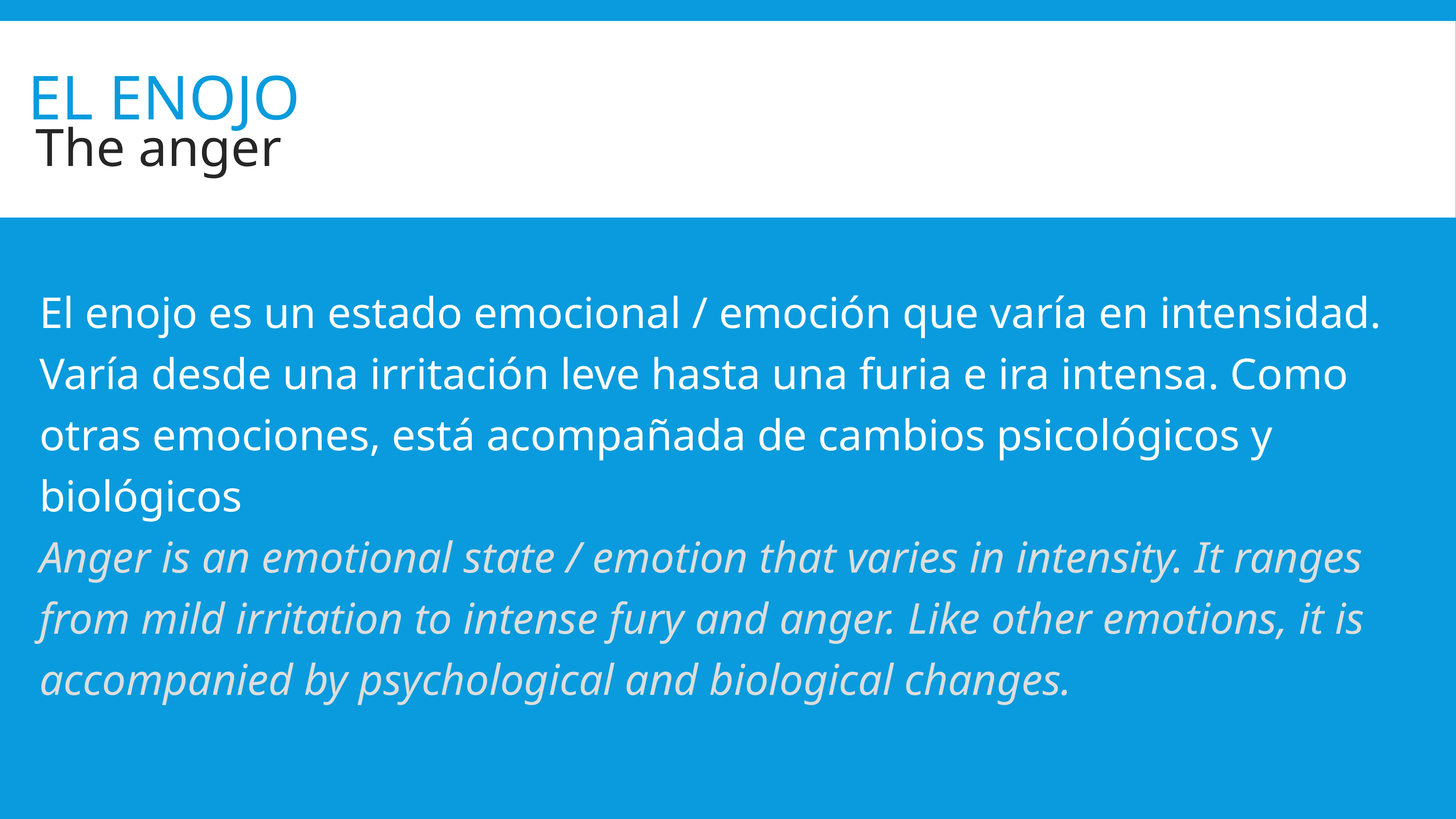

# El enojo
The anger
El enojo es un estado emocional / emoción que varía en intensidad. Varía desde una irritación leve hasta una furia e ira intensa. Como otras emociones, está acompañada de cambios psicológicos y biológicos
Anger is an emotional state / emotion that varies in intensity. It ranges from mild irritation to intense fury and anger. Like other emotions, it is accompanied by psychological and biological changes.

## Slide 11
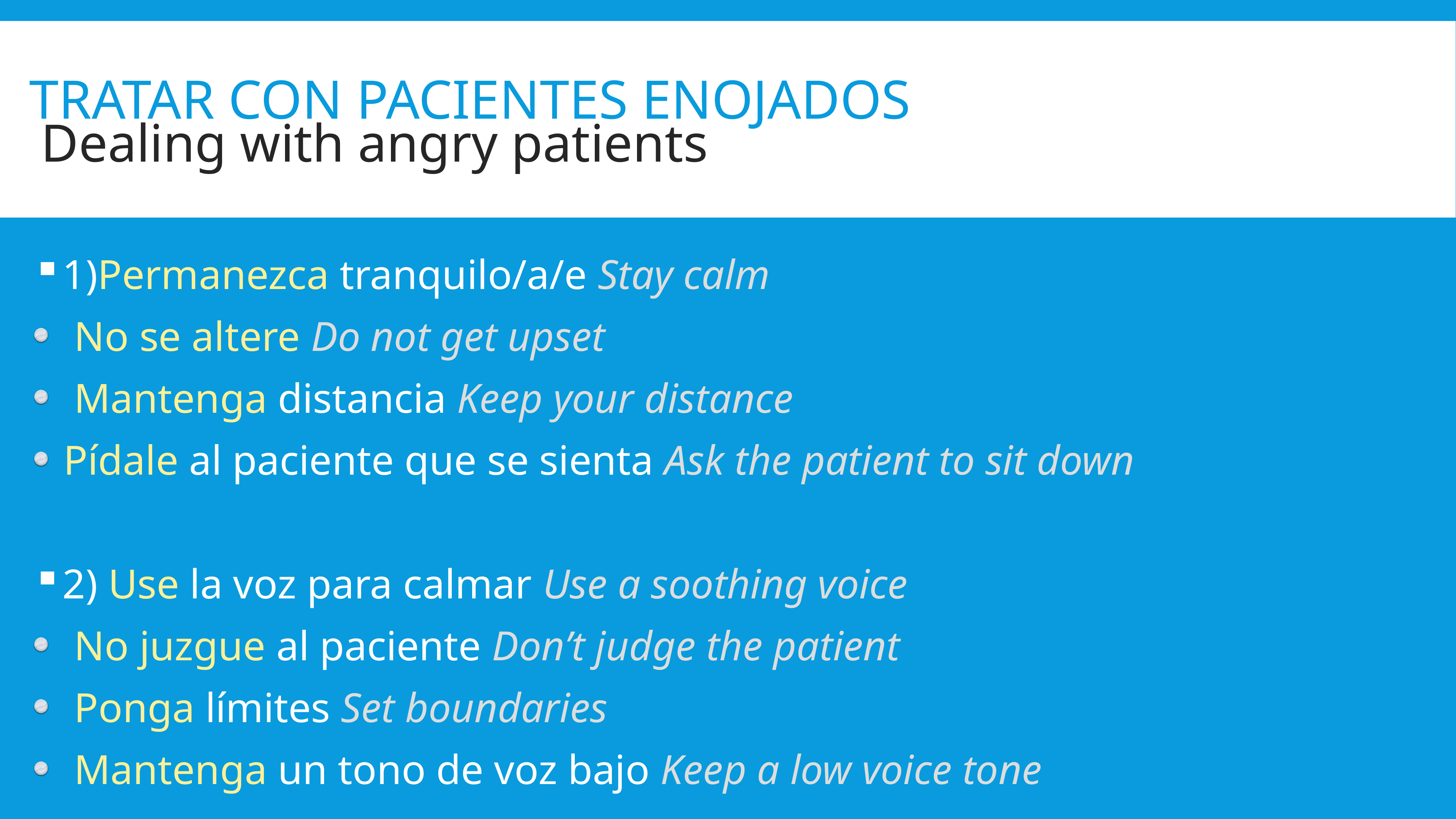

# Tratar con pacientes enojados
Dealing with angry patients
1)Permanezca tranquilo/a/e Stay calm
 No se altere Do not get upset
 Mantenga distancia Keep your distance
Pídale al paciente que se sienta Ask the patient to sit down
2) Use la voz para calmar Use a soothing voice
 No juzgue al paciente Don’t judge the patient
 Ponga límites Set boundaries
 Mantenga un tono de voz bajo Keep a low voice tone

## Slide 12
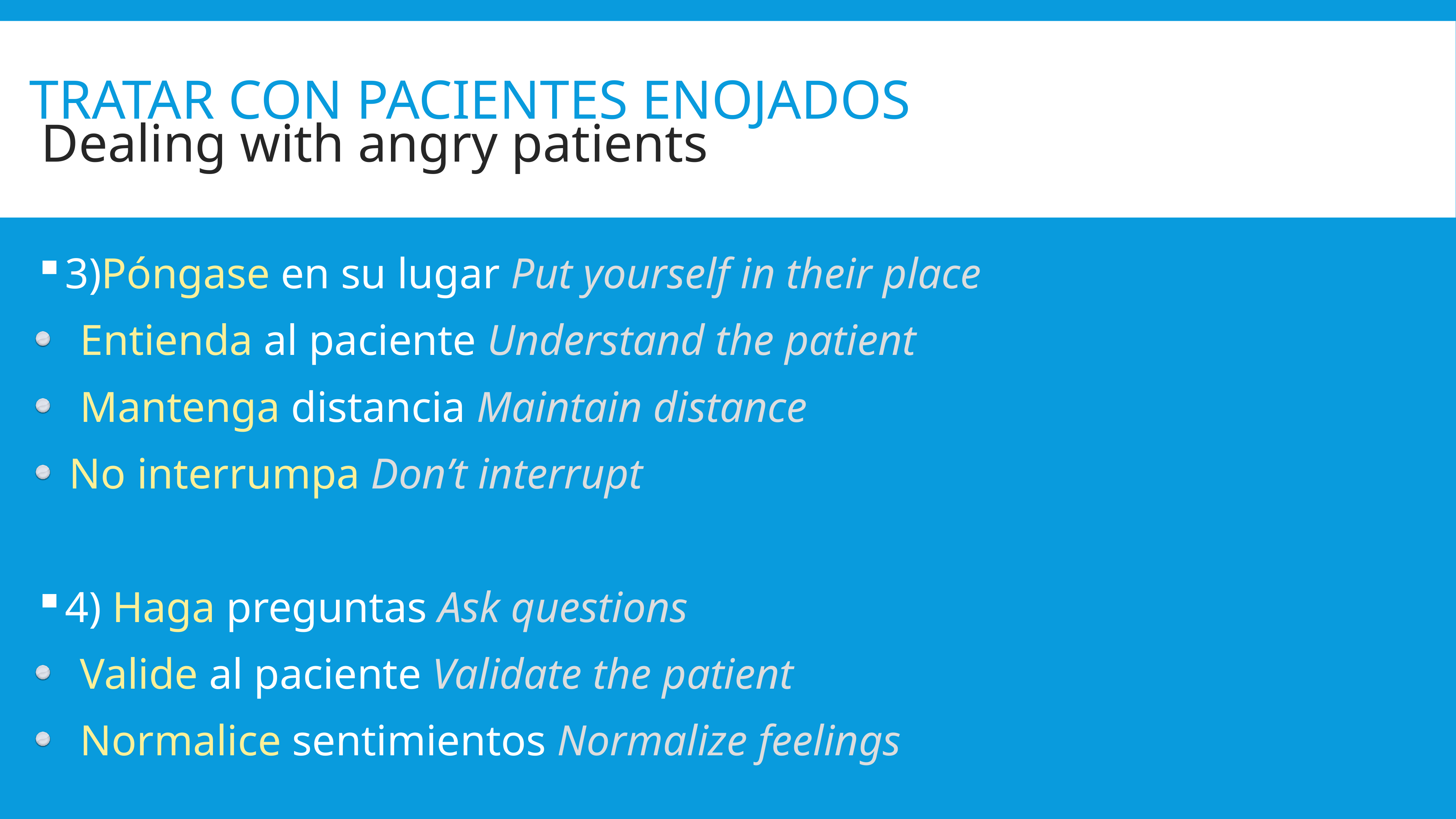

# Tratar con pacientes enojados
Dealing with angry patients
3)Póngase en su lugar Put yourself in their place
 Entienda al paciente Understand the patient
 Mantenga distancia Maintain distance
No interrumpa Don’t interrupt
4) Haga preguntas Ask questions
 Valide al paciente Validate the patient
 Normalice sentimientos Normalize feelings

## Slide 13
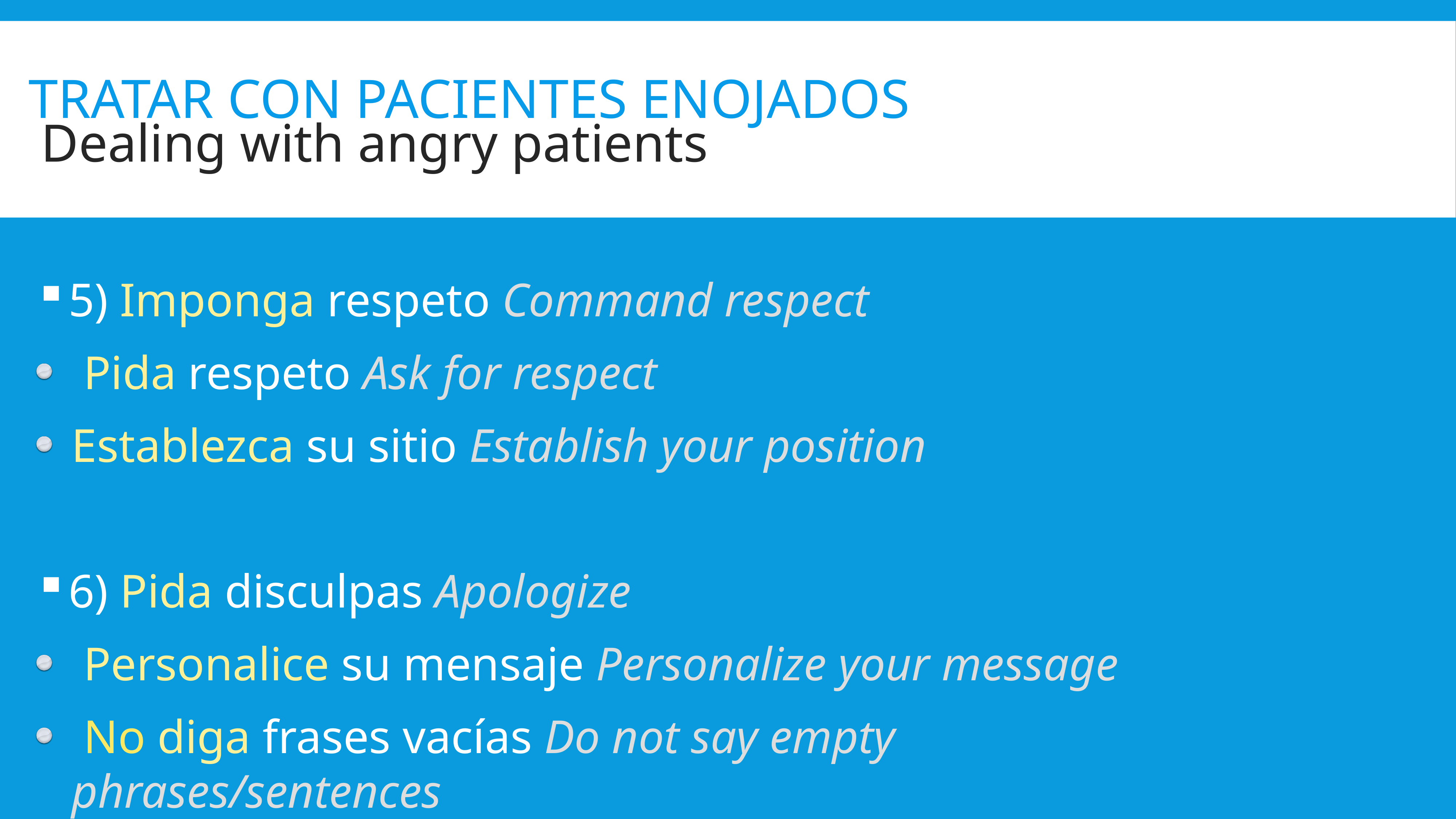

# Tratar con pacientes enojados
Dealing with angry patients
5) Imponga respeto Command respect
 Pida respeto Ask for respect
Establezca su sitio Establish your position
6) Pida disculpas Apologize
 Personalice su mensaje Personalize your message
 No diga frases vacías Do not say empty phrases/sentences

## Slide 14
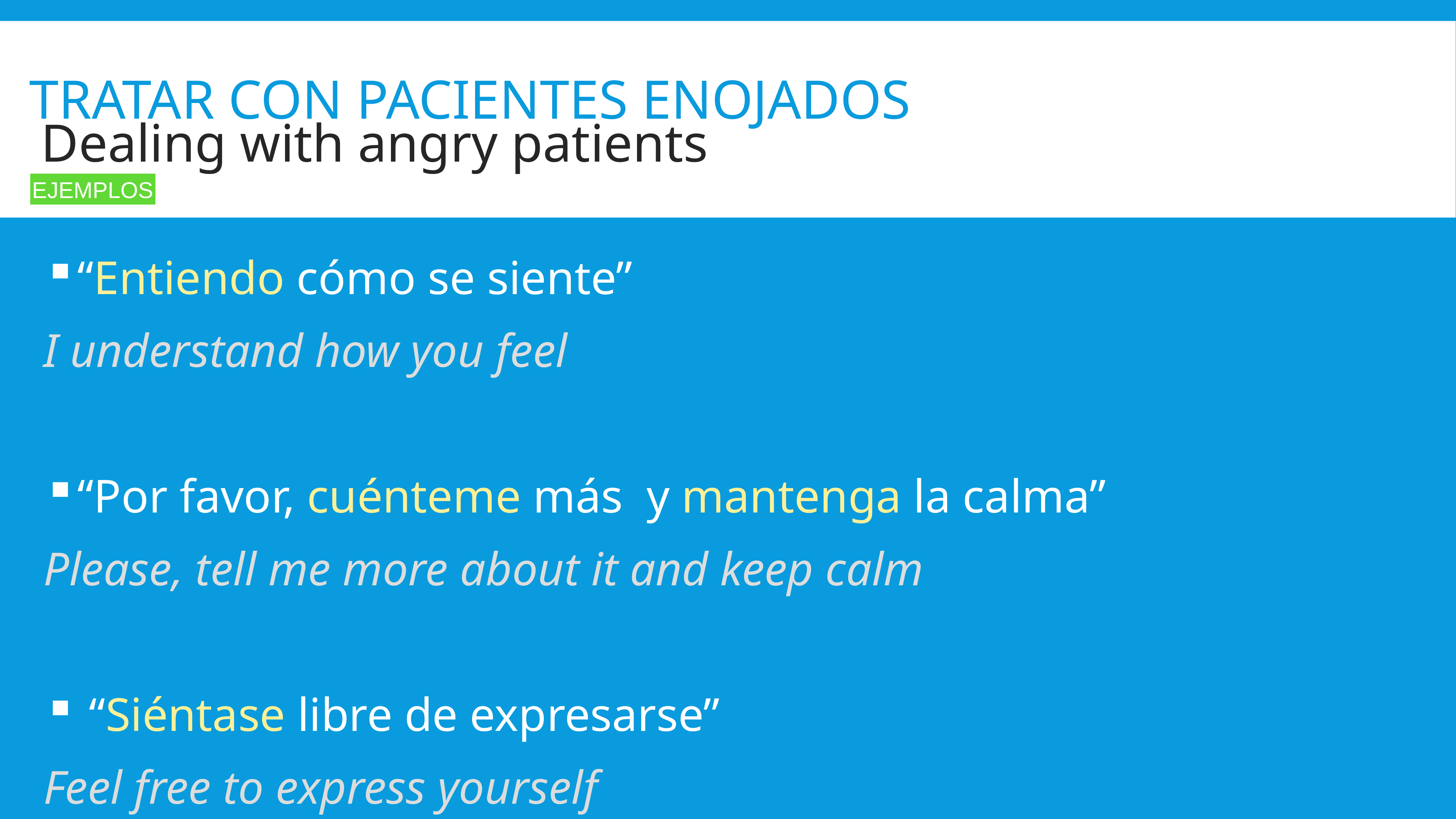

# Tratar con pacientes enojados
Dealing with angry patients
EJEMPLOS
“Entiendo cómo se siente”
I understand how you feel
“Por favor, cuénteme más  y mantenga la calma”
Please, tell me more about it and keep calm
 “Siéntase libre de expresarse”
Feel free to express yourself

## Slide 15
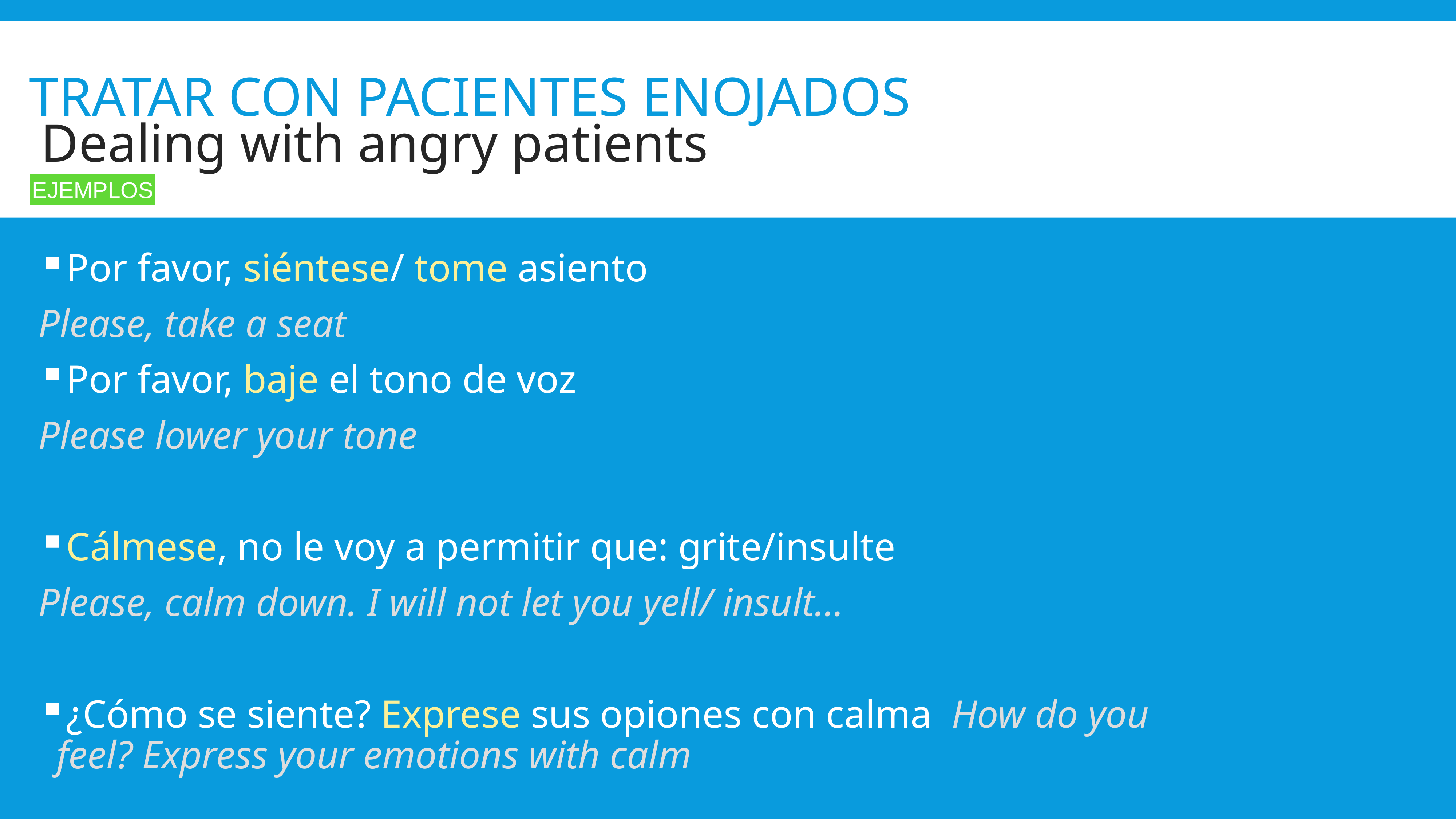

# Tratar con pacientes enojados
Dealing with angry patients
EJEMPLOS
Por favor, siéntese/ tome asiento
Please, take a seat
Por favor, baje el tono de voz
Please lower your tone
Cálmese, no le voy a permitir que: grite/insulte
Please, calm down. I will not let you yell/ insult…
¿Cómo se siente? Exprese sus opiones con calma How do you feel? Express your emotions with calm

## Slide 16
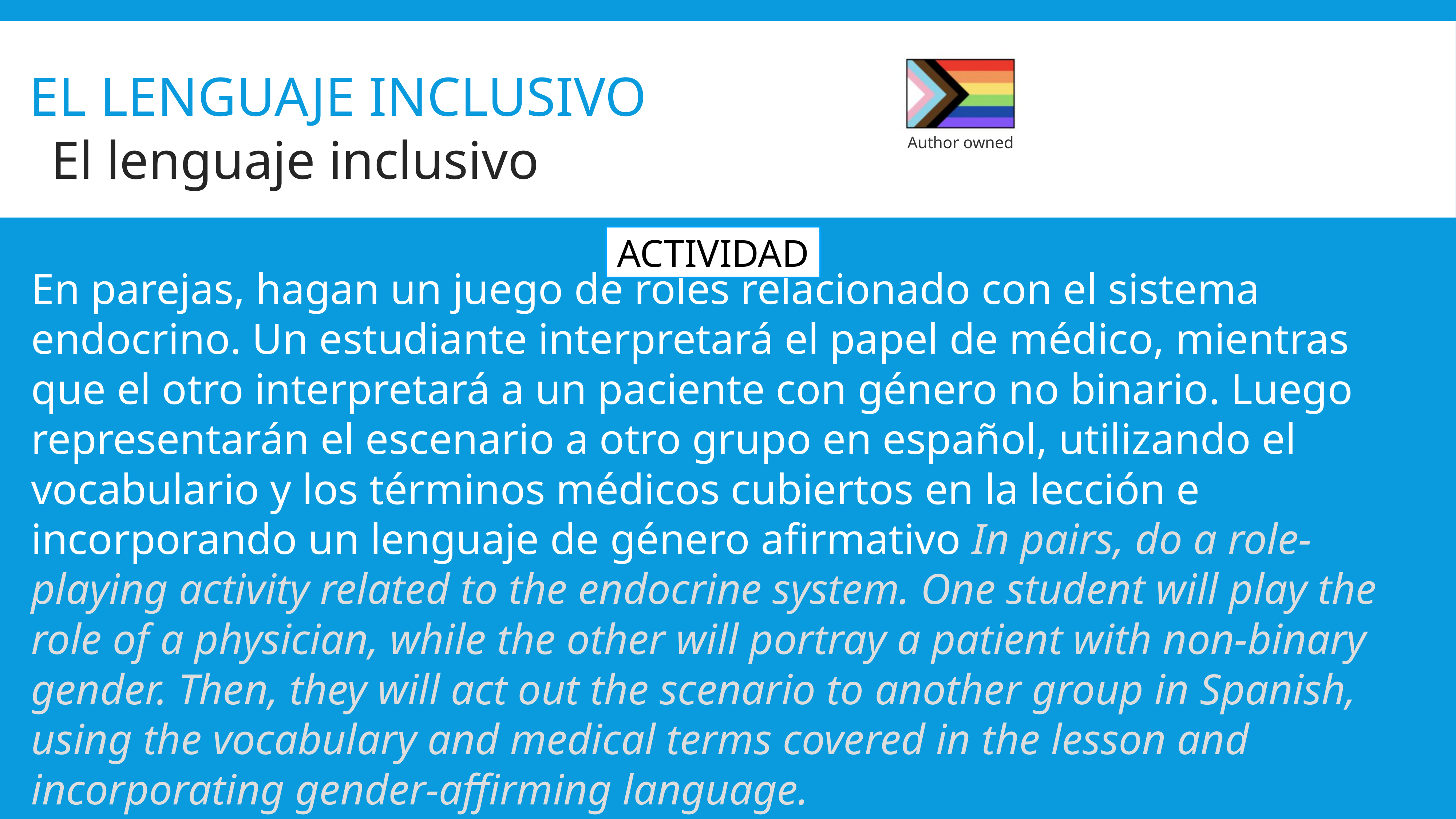

# El lenguaje inclusivo
El lenguaje inclusivo
Author owned
ACTIVIDAD
En parejas, hagan un juego de roles relacionado con el sistema endocrino. Un estudiante interpretará el papel de médico, mientras que el otro interpretará a un paciente con género no binario. Luego representarán el escenario a otro grupo en español, utilizando el vocabulario y los términos médicos cubiertos en la lección e incorporando un lenguaje de género afirmativo In pairs, do a role-playing activity related to the endocrine system. One student will play the role of a physician, while the other will portray a patient with non-binary gender. Then, they will act out the scenario to another group in Spanish, using the vocabulary and medical terms covered in the lesson and incorporating gender-affirming language.

## Slide 17
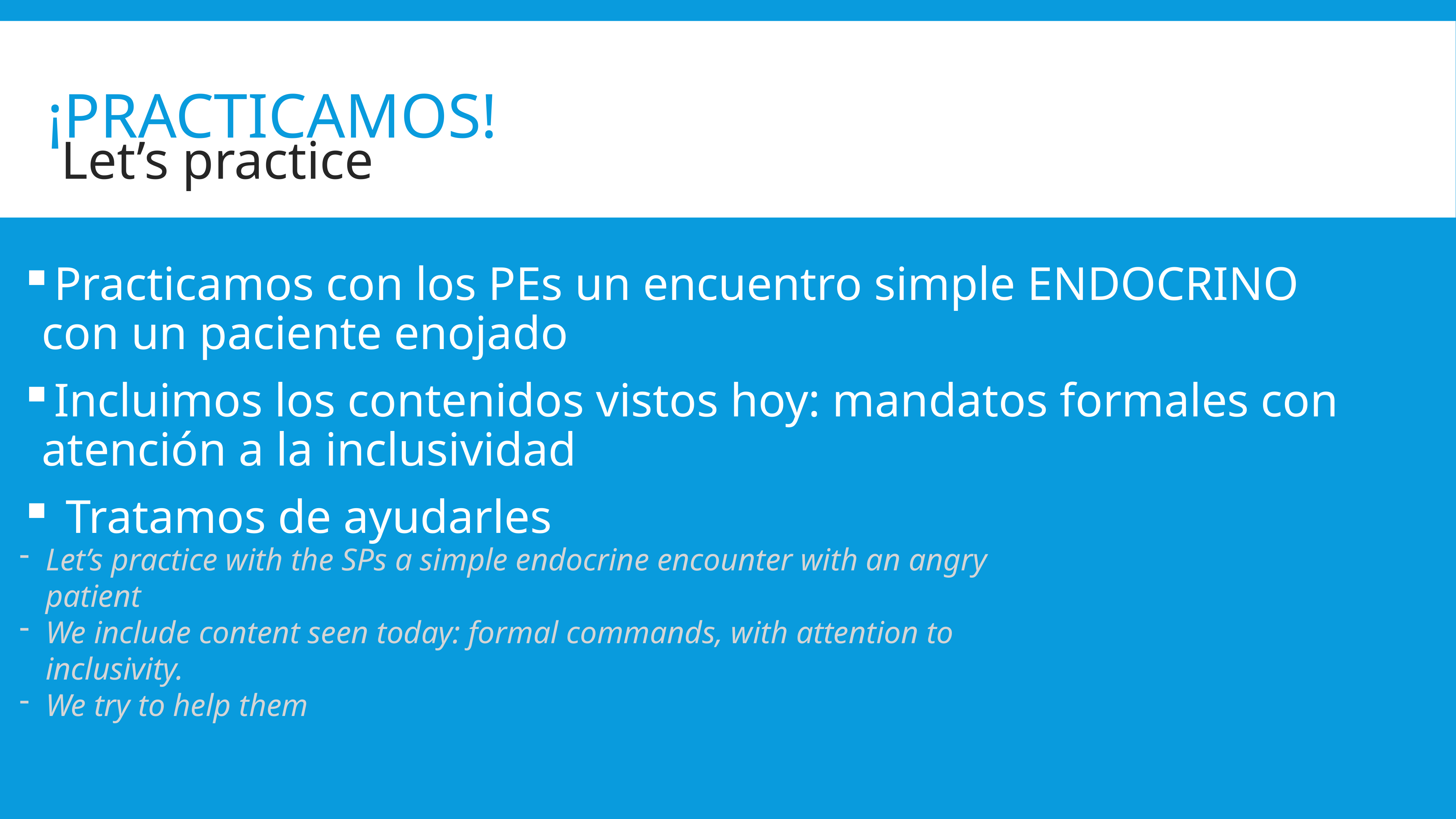

# ¡Practicamos!
Let’s practice
Practicamos con los PEs un encuentro simple ENDOCRINO con un paciente enojado
Incluimos los contenidos vistos hoy: mandatos formales con atención a la inclusividad
 Tratamos de ayudarles
Let’s practice with the SPs a simple endocrine encounter with an angry patient
We include content seen today: formal commands, with attention to inclusivity.
We try to help them

## Slide 18
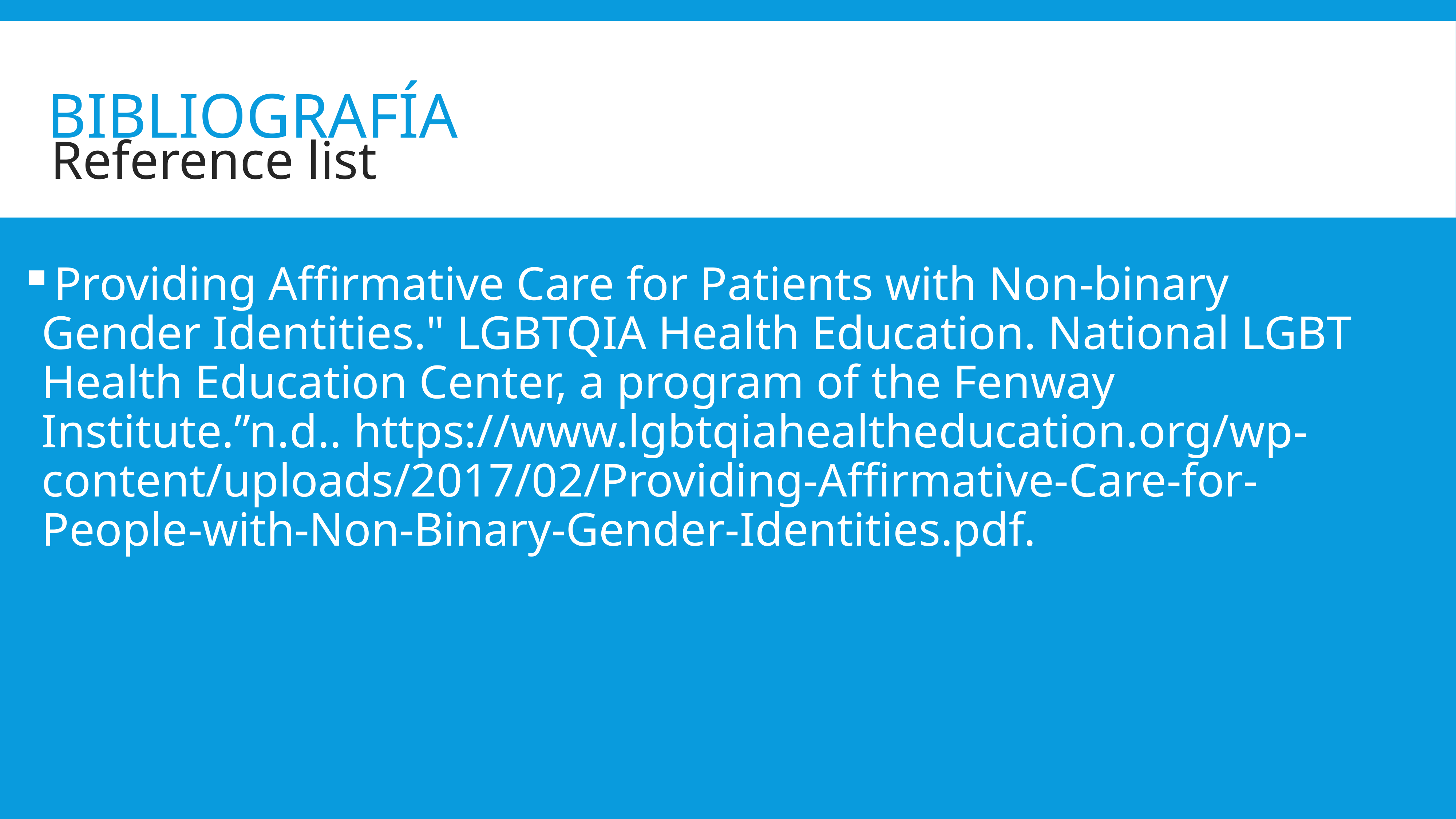

# BIBLIOGRAfía
Reference list
Providing Affirmative Care for Patients with Non-binary Gender Identities." LGBTQIA Health Education. National LGBT Health Education Center, a program of the Fenway Institute.”n.d.. https://www.lgbtqiahealtheducation.org/wp-content/uploads/2017/02/Providing-Affirmative-Care-for-People-with-Non-Binary-Gender-Identities.pdf.
