# Supplementary material for: Integrating Gender-Affirming Care in a Medical Spanish Endocrine System Curriculum
Source: MedEdPORTAL. 2024 Oct 23;20:11456. doi: 10.15766/mep_2374-8265.11456 (PMC11496385; doi:10.15766/mep_2374-8265.11456)
Supplement: Supplementary file 1 — Facilitator Guide.docxLesson 1 Presentation.pptxLesson 2 Presentation.pptxLesson 3 Presentation.pptxLesson 1 Clinical Endocrine Checklist.docxLesson 2 Clinical Endocrine Checklist.docxLesson 3 Clinical Endocrine Checklist.docxLesson 1 SP Case.docxLesson 2 SP Case.docxLesson 3 SP Case.docxPre-Post Confidence Survey.docxPre-Post Spanish Endocrine Test.docxOSCE SP Diabetic Case.docxOSCE Door Note.docxOSCE Clinical Checklist Diabetic Encounter.docxOSCE Language Rubric for Diabetic Encounter.docx [file mep_2374-8265.11456-s001.zip › D. Lesson 3 Presentation.pptx]

## Slide 1
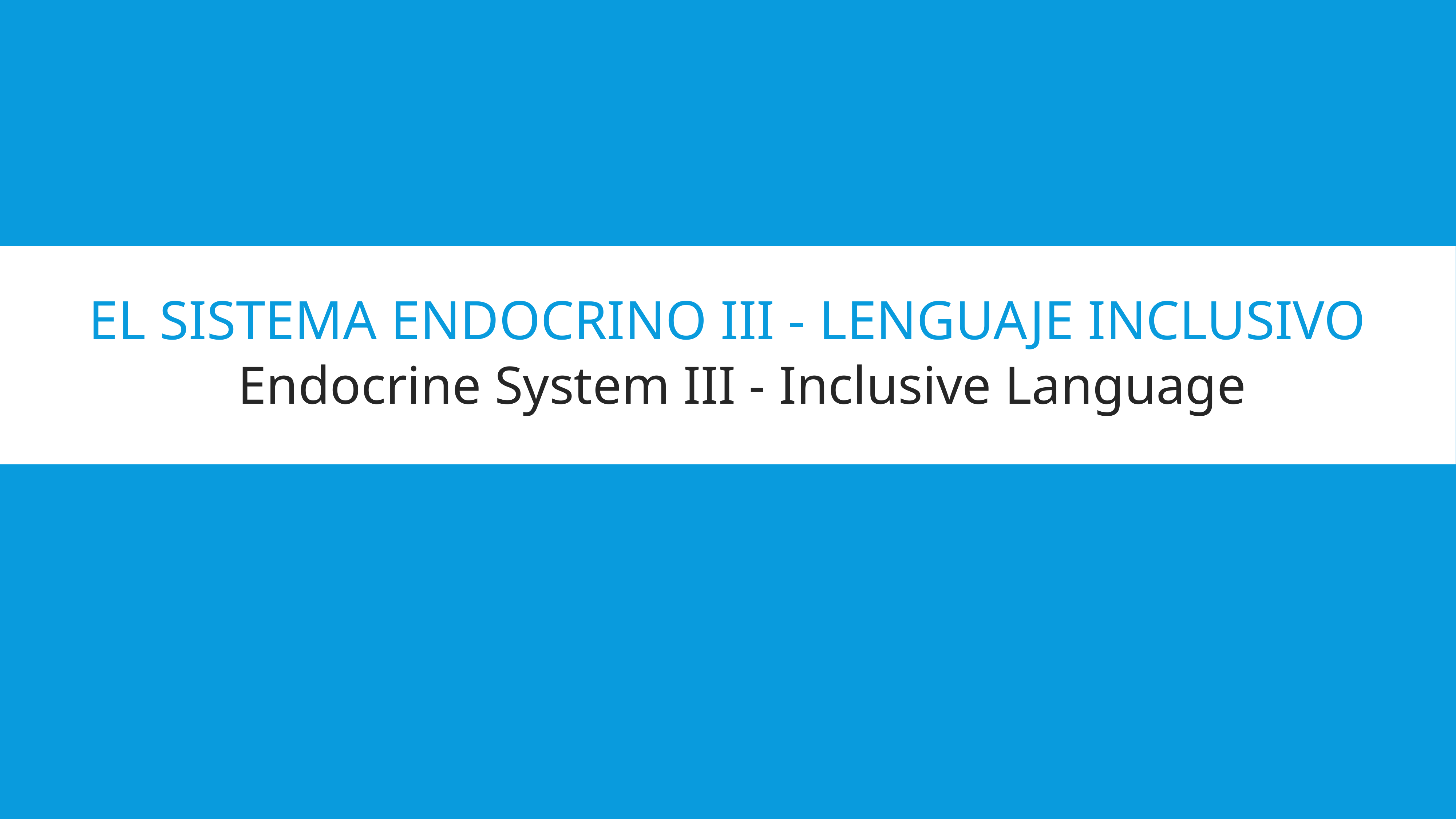

# El sistema endocrino iII - lenguaje inclusivo
Endocrine System III - Inclusive Language

## Slide 2
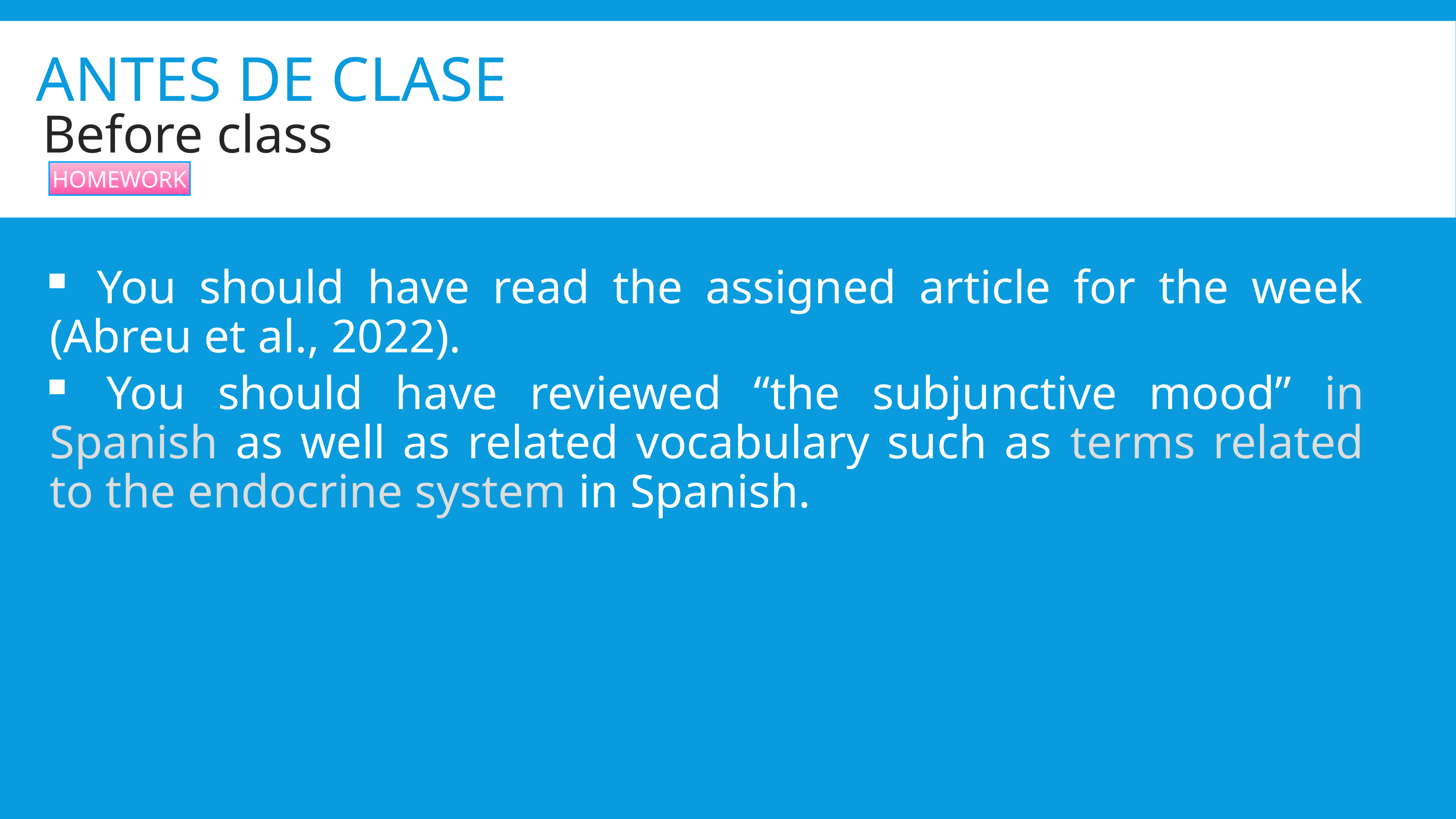

# Antes de clase
Before class
HOMEWORK
 You should have read the assigned article for the week (Abreu et al., 2022).
 You should have reviewed “the subjunctive mood” in Spanish as well as related vocabulary such as terms related to the endocrine system in Spanish.

## Slide 3
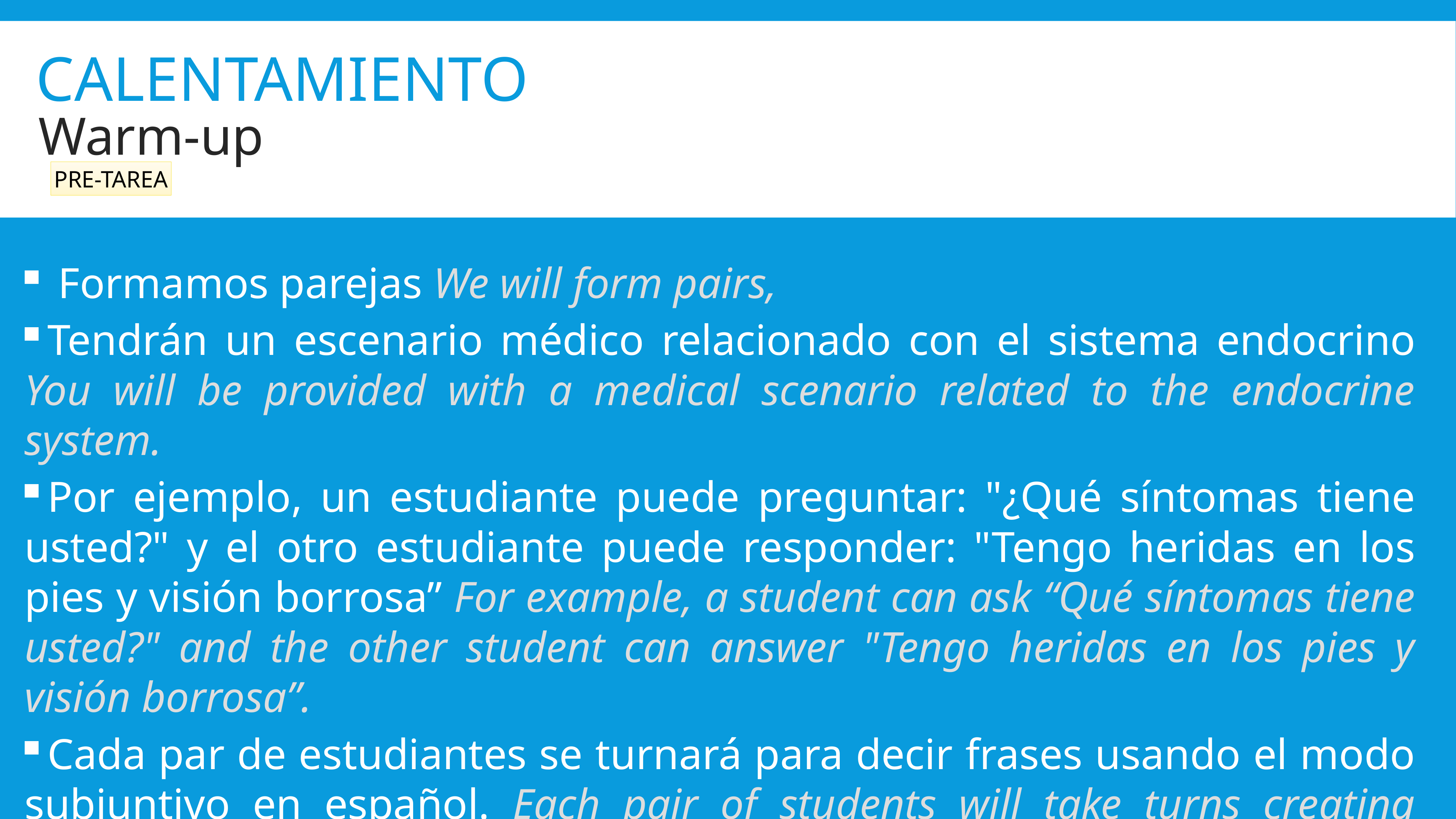

# calentamiento
Warm-up
PRE-TAREA
 Formamos parejas We will form pairs,
Tendrán un escenario médico relacionado con el sistema endocrino You will be provided with a medical scenario related to the endocrine system.
Por ejemplo, un estudiante puede preguntar: "¿Qué síntomas tiene usted?" y el otro estudiante puede responder: "Tengo heridas en los pies y visión borrosa” For example, a student can ask “Qué síntomas tiene usted?" and the other student can answer "Tengo heridas en los pies y visión borrosa”.
Cada par de estudiantes se turnará para decir frases usando el modo subjuntivo en español. Each pair of students will take turns creating sentences using the subjunctive mood in Spanish.

## Slide 4
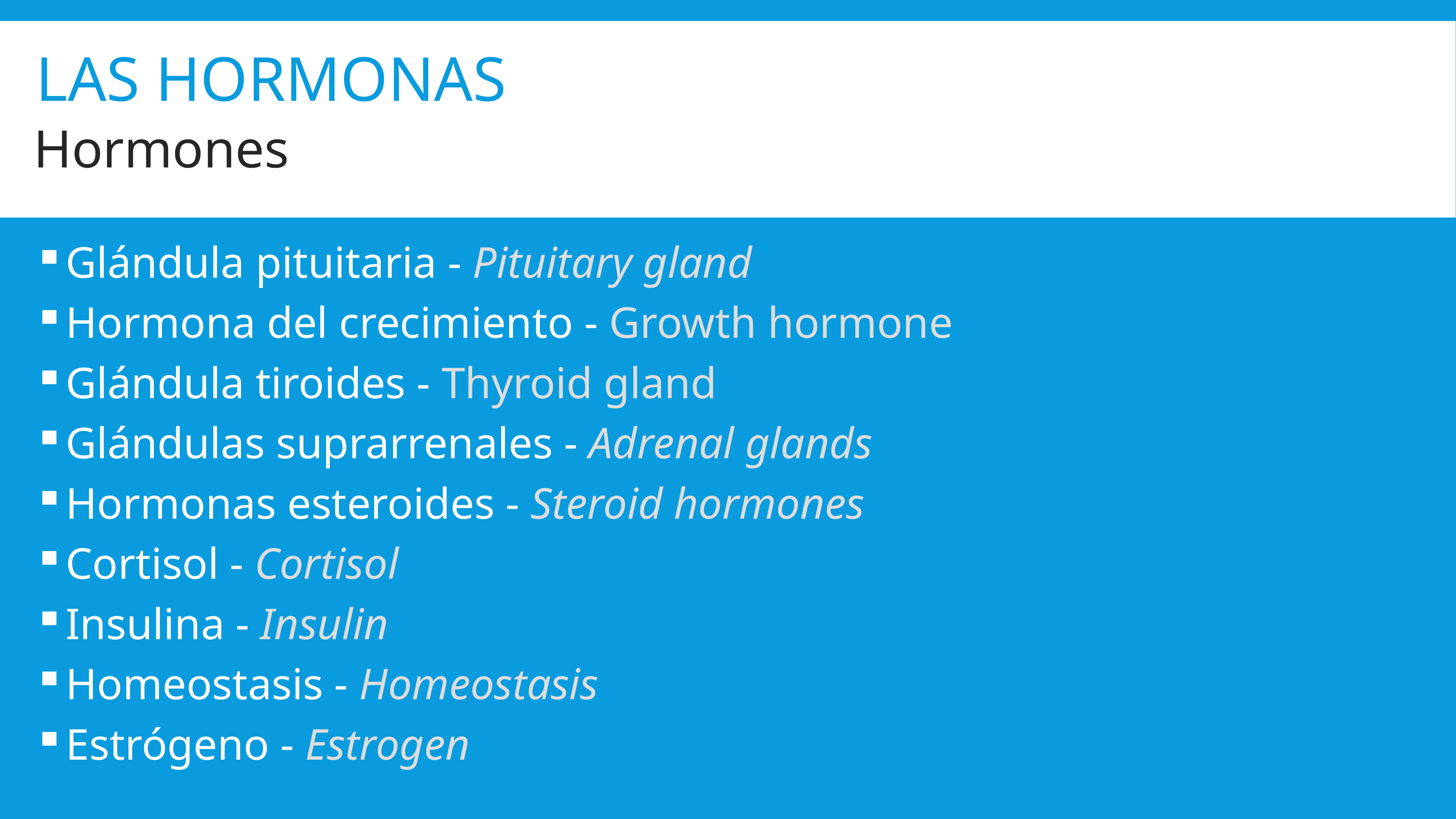

# Las hormonas
Hormones
Glándula pituitaria - Pituitary gland
Hormona del crecimiento - Growth hormone
Glándula tiroides - Thyroid gland
Glándulas suprarrenales - Adrenal glands
Hormonas esteroides - Steroid hormones
Cortisol - Cortisol
Insulina - Insulin
Homeostasis - Homeostasis
Estrógeno - Estrogen

## Slide 5
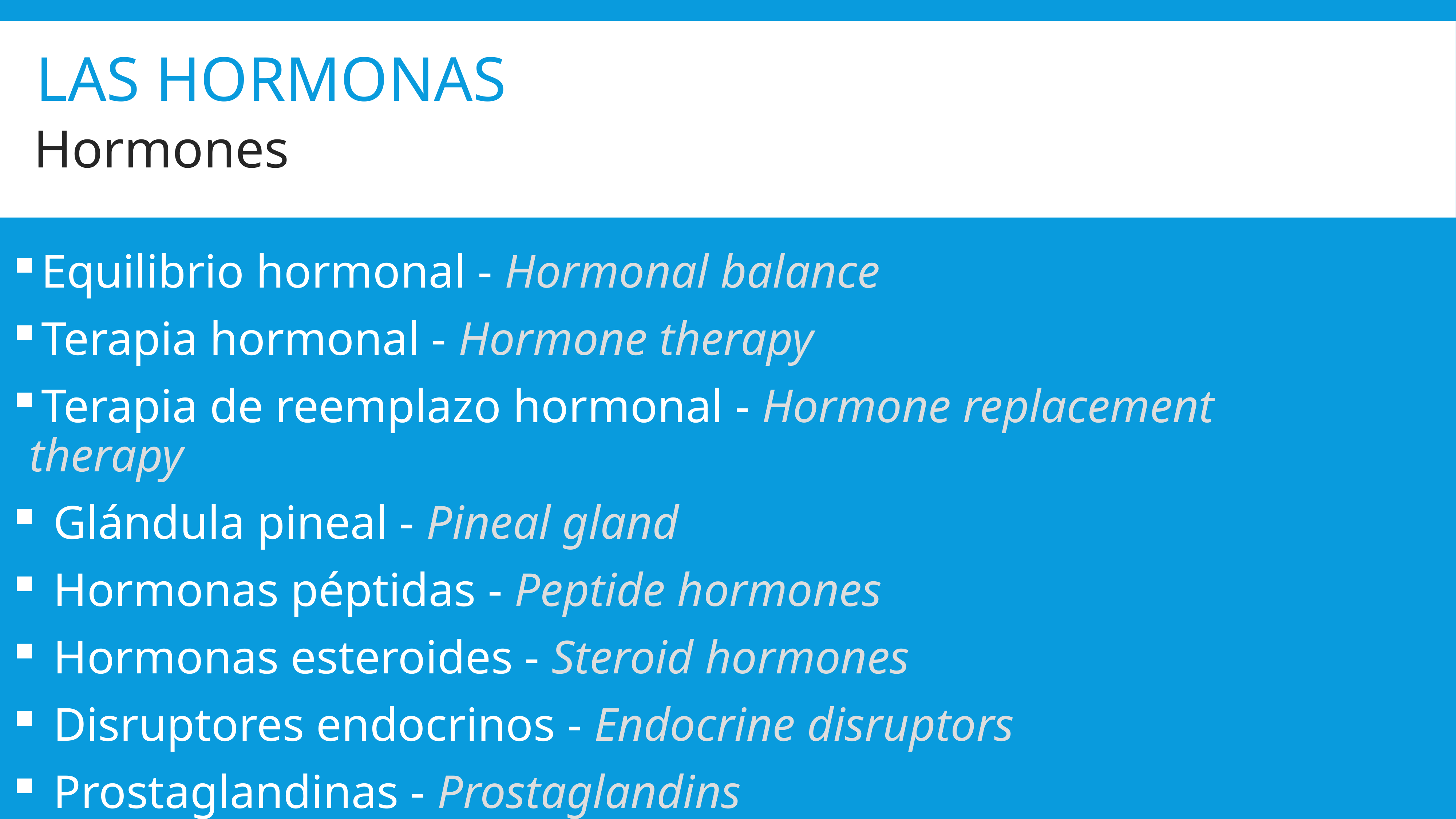

# Las hormonas
Hormones
Equilibrio hormonal - Hormonal balance
Terapia hormonal - Hormone therapy
Terapia de reemplazo hormonal - Hormone replacement therapy
 Glándula pineal - Pineal gland
 Hormonas péptidas - Peptide hormones
 Hormonas esteroides - Steroid hormones
 Disruptores endocrinos - Endocrine disruptors
 Prostaglandinas - Prostaglandins

## Slide 6
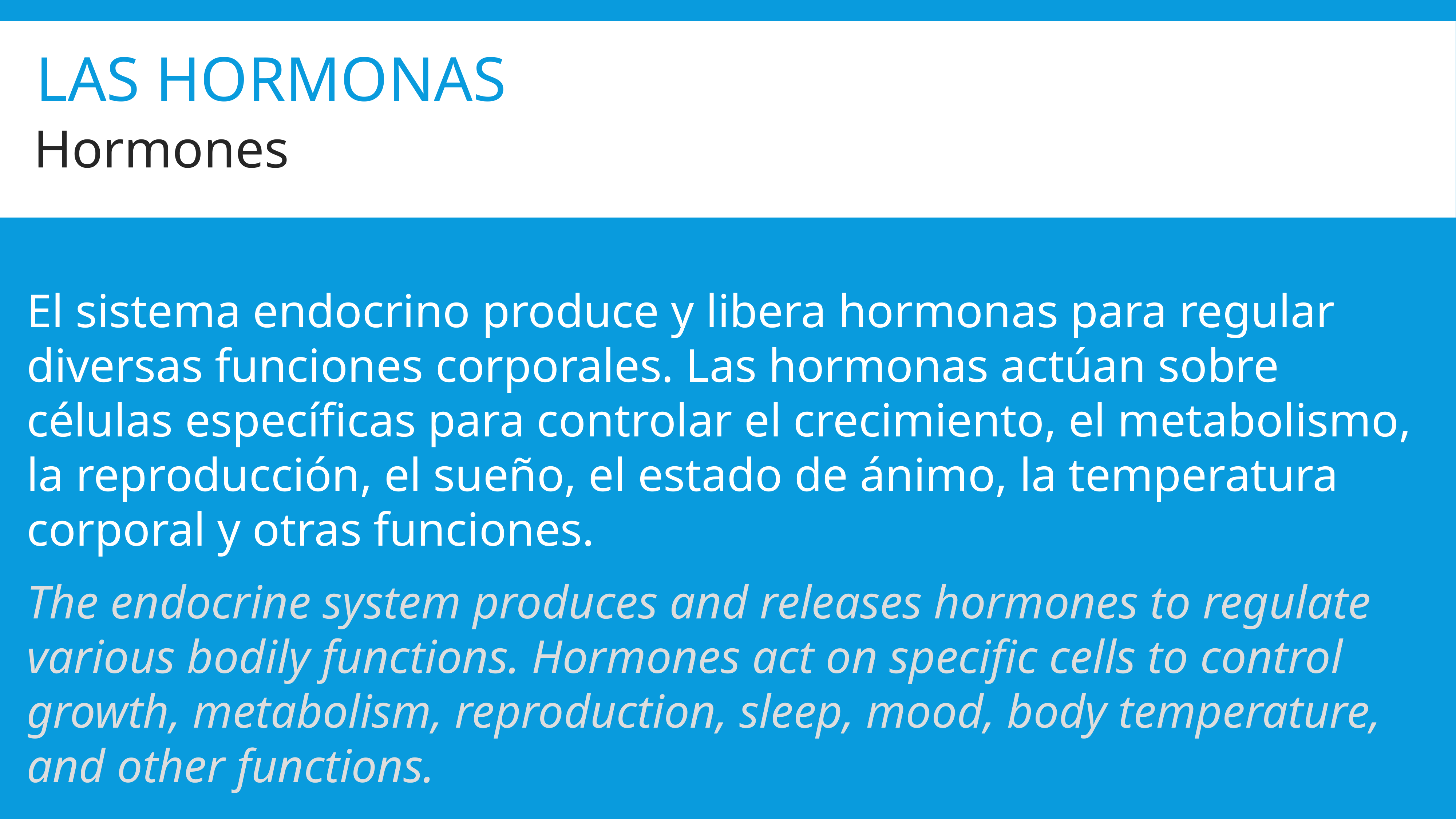

# Las hormonas
Hormones
El sistema endocrino produce y libera hormonas para regular diversas funciones corporales. Las hormonas actúan sobre células específicas para controlar el crecimiento, el metabolismo, la reproducción, el sueño, el estado de ánimo, la temperatura corporal y otras funciones.
The endocrine system produces and releases hormones to regulate various bodily functions. Hormones act on specific cells to control growth, metabolism, reproduction, sleep, mood, body temperature, and other functions.

## Slide 7
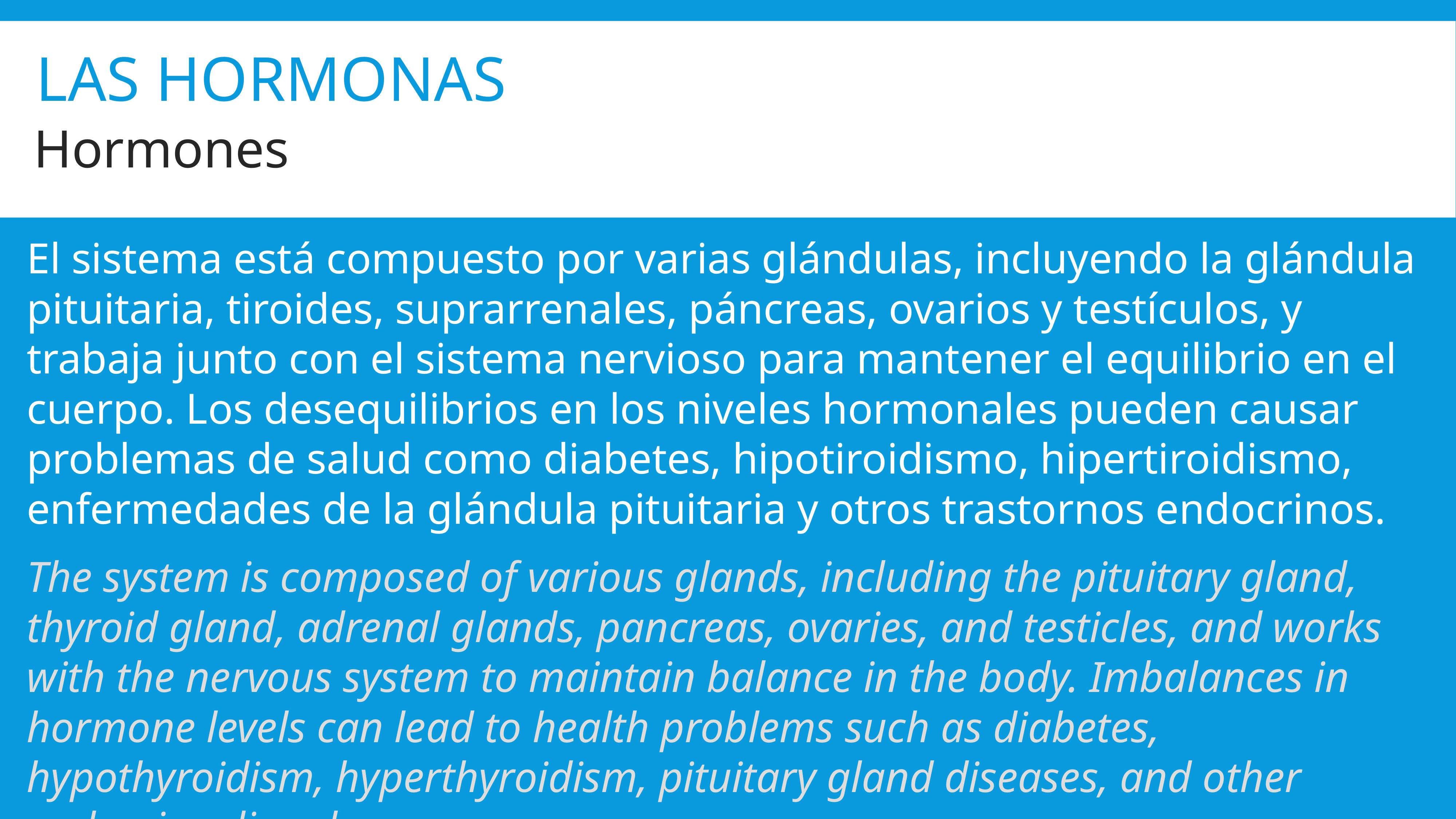

# Las hormonas
Hormones
El sistema está compuesto por varias glándulas, incluyendo la glándula pituitaria, tiroides, suprarrenales, páncreas, ovarios y testículos, y trabaja junto con el sistema nervioso para mantener el equilibrio en el cuerpo. Los desequilibrios en los niveles hormonales pueden causar problemas de salud como diabetes, hipotiroidismo, hipertiroidismo, enfermedades de la glándula pituitaria y otros trastornos endocrinos.
The system is composed of various glands, including the pituitary gland, thyroid gland, adrenal glands, pancreas, ovaries, and testicles, and works with the nervous system to maintain balance in the body. Imbalances in hormone levels can lead to health problems such as diabetes, hypothyroidism, hyperthyroidism, pituitary gland diseases, and other endocrine disorders.

## Slide 8
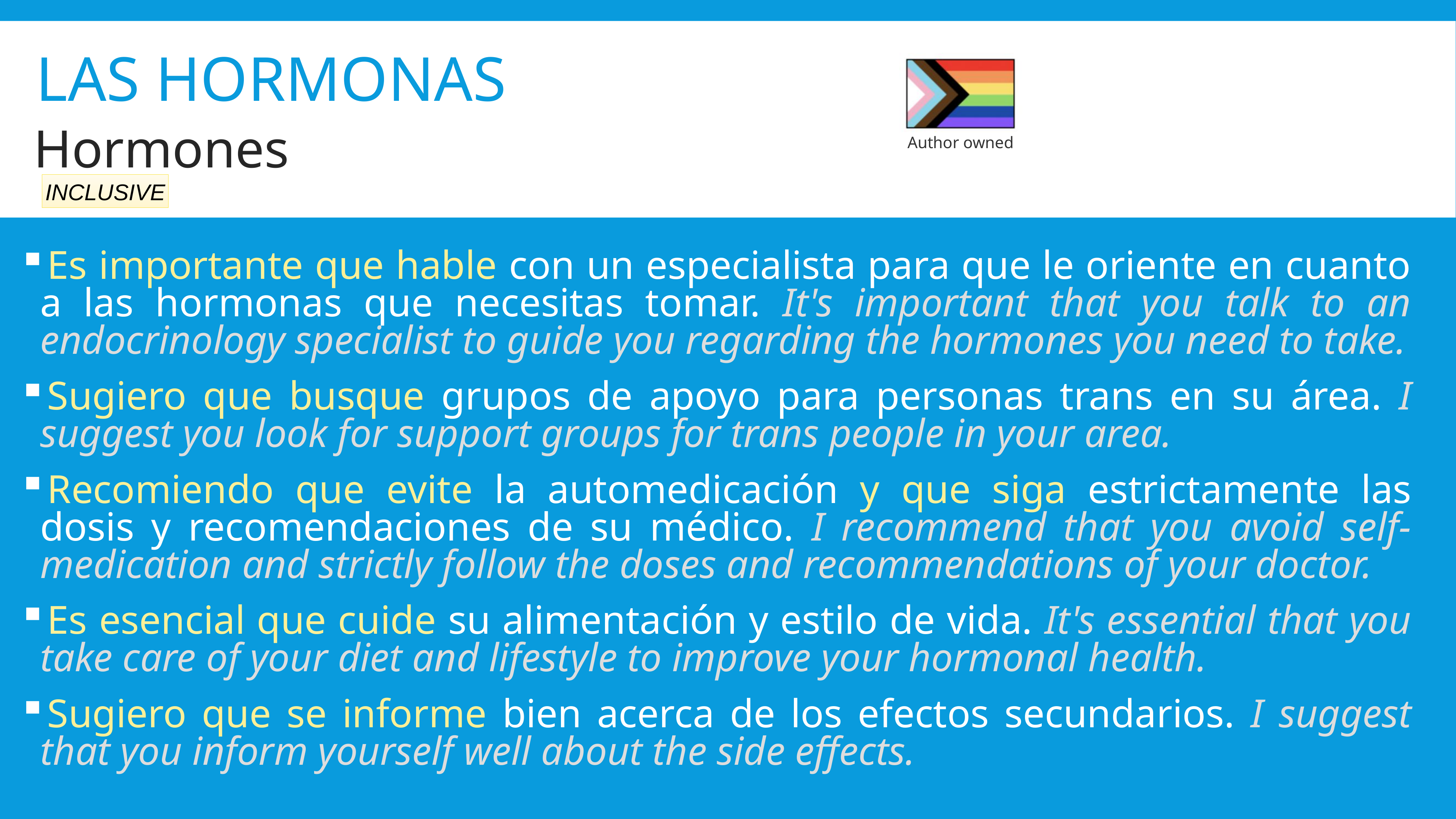

# Las hormonas
Hormones
Author owned
INCLUSIVE
Es importante que hable con un especialista para que le oriente en cuanto a las hormonas que necesitas tomar. It's important that you talk to an endocrinology specialist to guide you regarding the hormones you need to take.
Sugiero que busque grupos de apoyo para personas trans en su área. I suggest you look for support groups for trans people in your area.
Recomiendo que evite la automedicación y que siga estrictamente las dosis y recomendaciones de su médico. I recommend that you avoid self-medication and strictly follow the doses and recommendations of your doctor.
Es esencial que cuide su alimentación y estilo de vida. It's essential that you take care of your diet and lifestyle to improve your hormonal health.
Sugiero que se informe bien acerca de los efectos secundarios. I suggest that you inform yourself well about the side effects.

## Slide 9
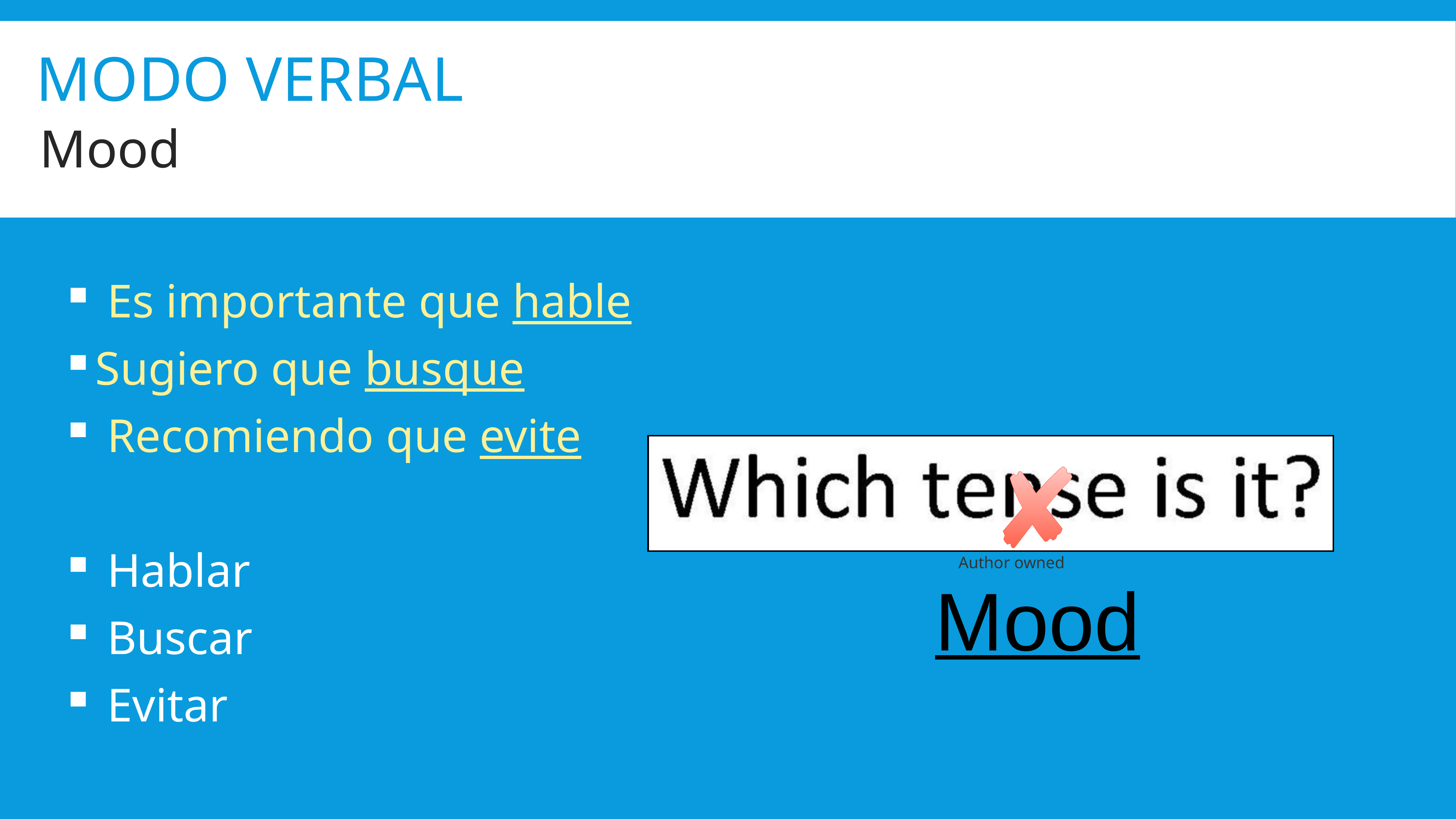

# Modo verbal
Mood
 Es importante que hable
Sugiero que busque
 Recomiendo que evite
 Hablar
 Buscar
 Evitar
Author owned
Mood

## Slide 10
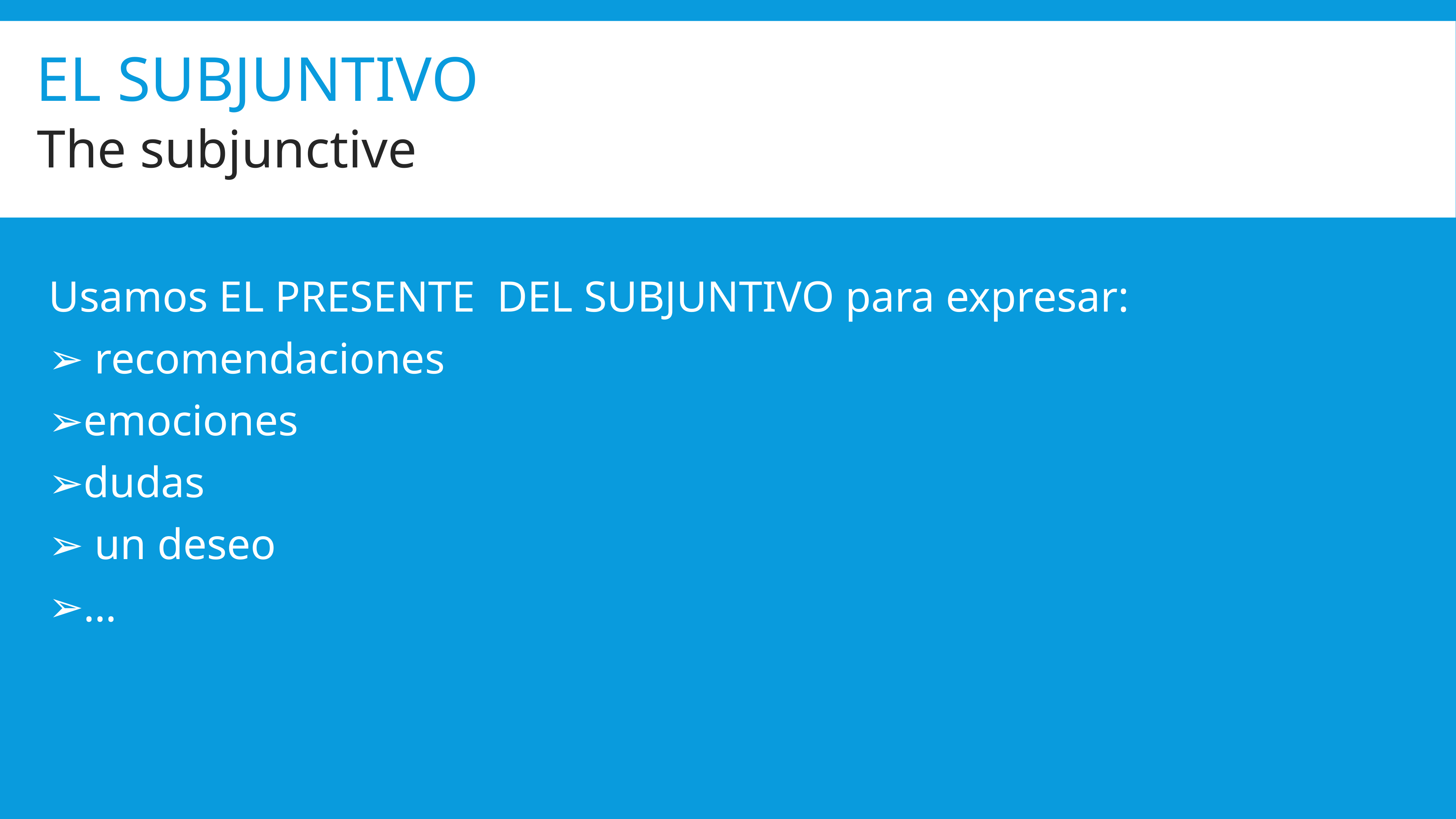

# El subjuntivo
The subjunctive
Usamos EL PRESENTE DEL SUBJUNTIVO para expresar:
 recomendaciones
emociones
dudas
 un deseo
…

## Slide 11
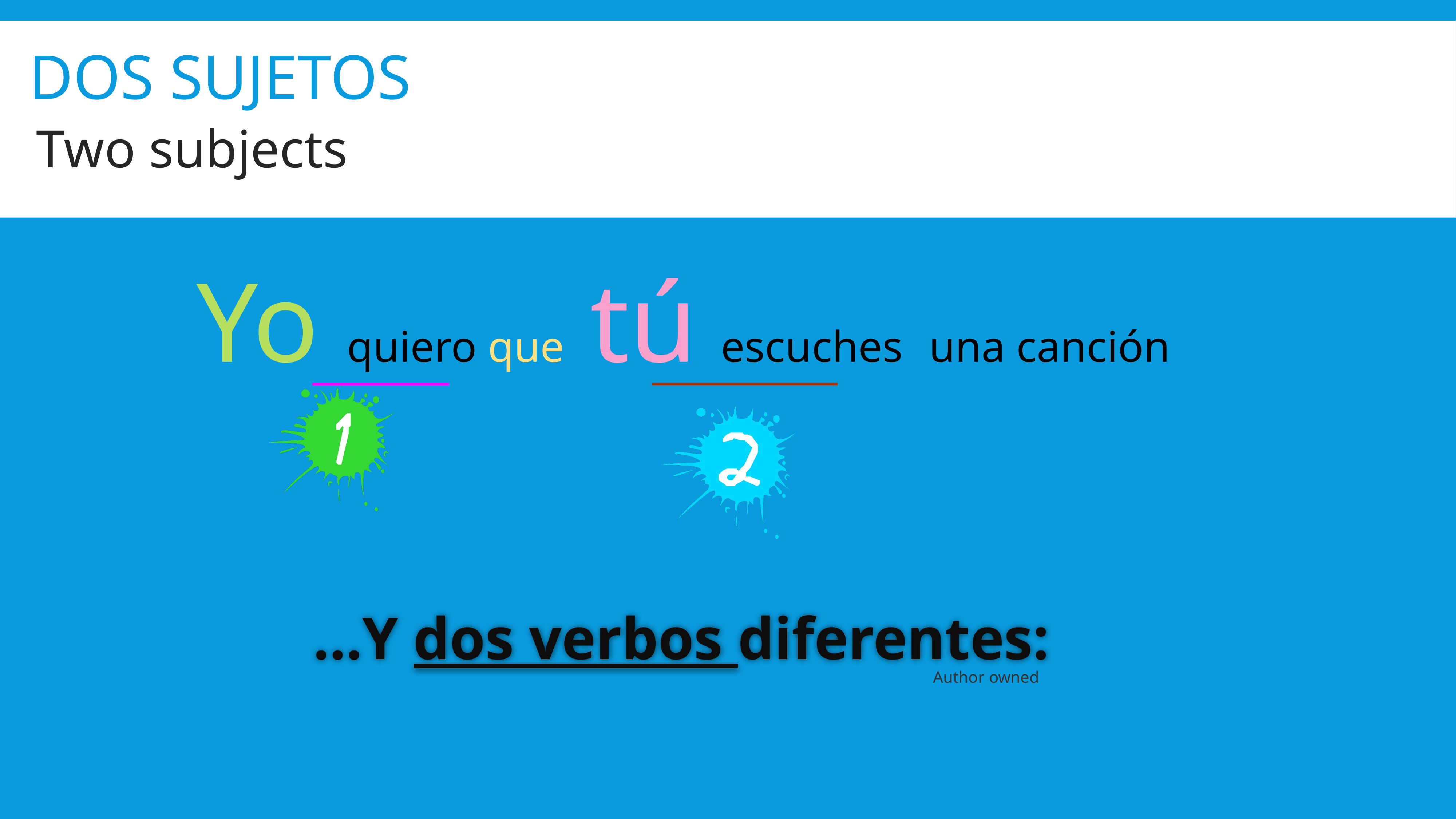

Dos sujetos
Two subjects
Yo quiero que tú escuches una canción
…Y dos verbos diferentes:
Author owned

## Slide 12
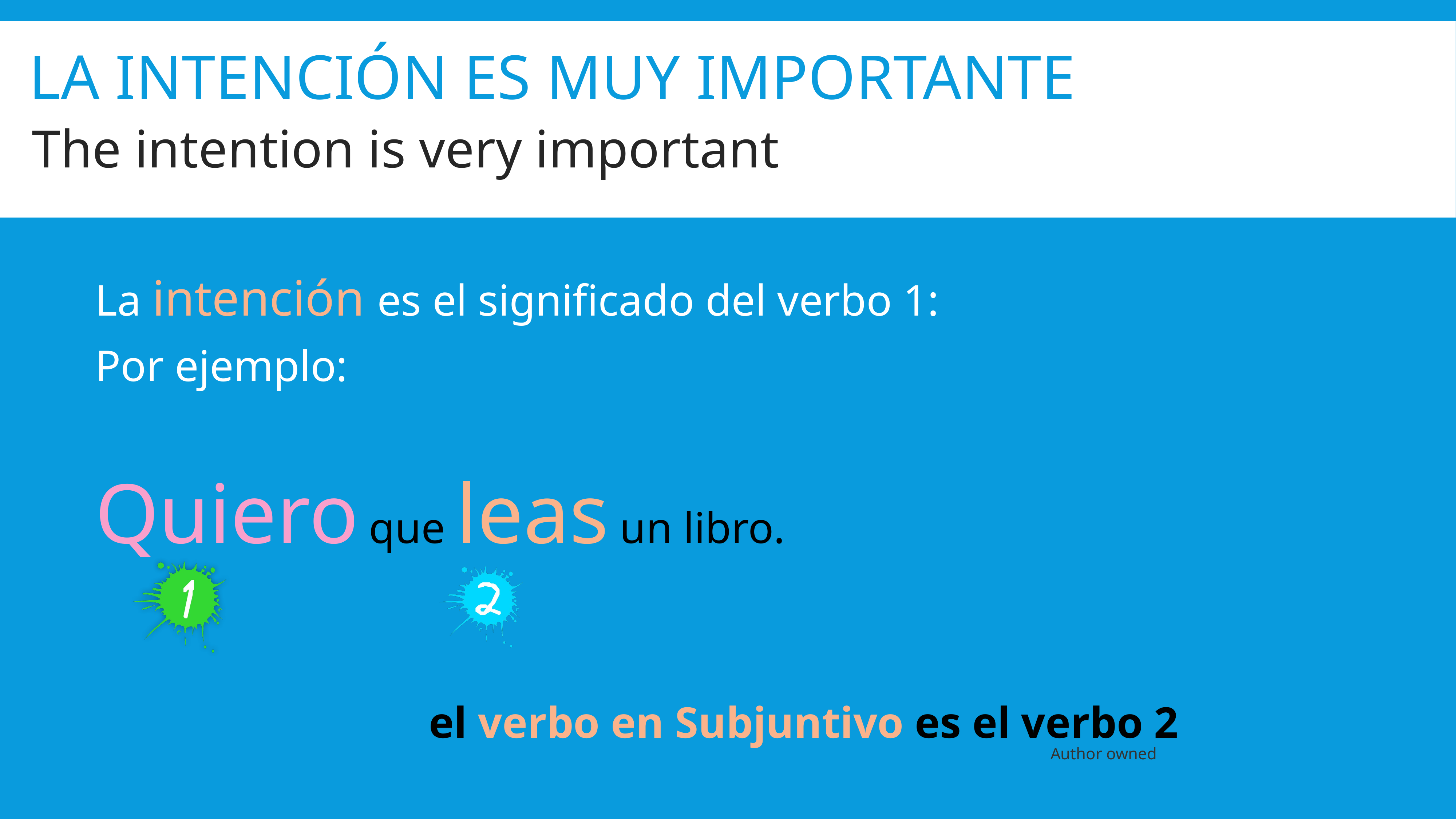

La intención es muy importante
The intention is very important
La intención es el significado del verbo 1:
Por ejemplo:
Quiero que leas un libro.
el verbo en Subjuntivo es el verbo 2
Author owned

## Slide 13
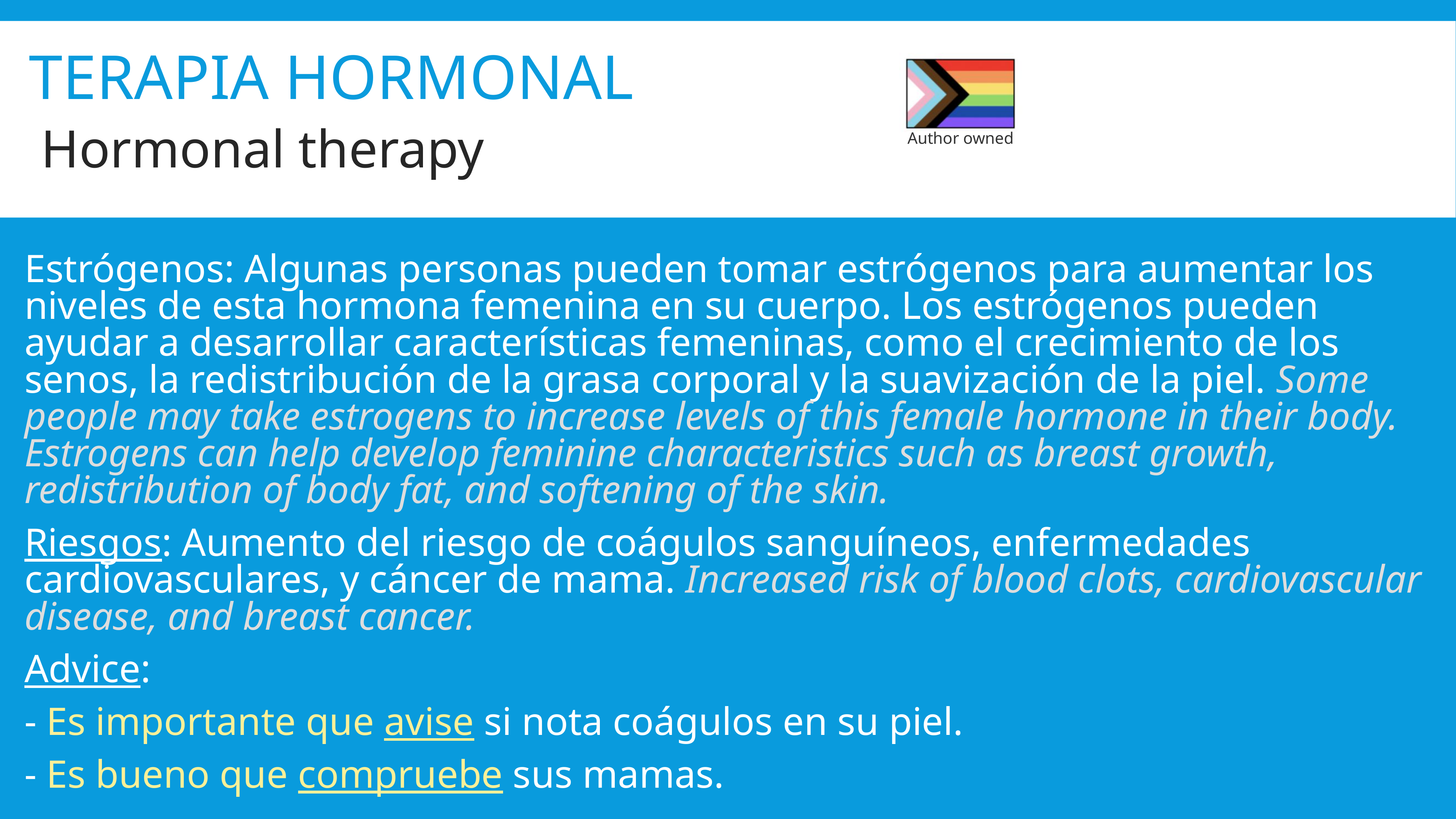

Terapia hormonal
Hormonal therapy
Author owned
Estrógenos: Algunas personas pueden tomar estrógenos para aumentar los niveles de esta hormona femenina en su cuerpo. Los estrógenos pueden ayudar a desarrollar características femeninas, como el crecimiento de los senos, la redistribución de la grasa corporal y la suavización de la piel. Some people may take estrogens to increase levels of this female hormone in their body. Estrogens can help develop feminine characteristics such as breast growth, redistribution of body fat, and softening of the skin.
Riesgos: Aumento del riesgo de coágulos sanguíneos, enfermedades cardiovasculares, y cáncer de mama. Increased risk of blood clots, cardiovascular disease, and breast cancer.
Advice:
- Es importante que avise si nota coágulos en su piel.
- Es bueno que compruebe sus mamas.

## Slide 14
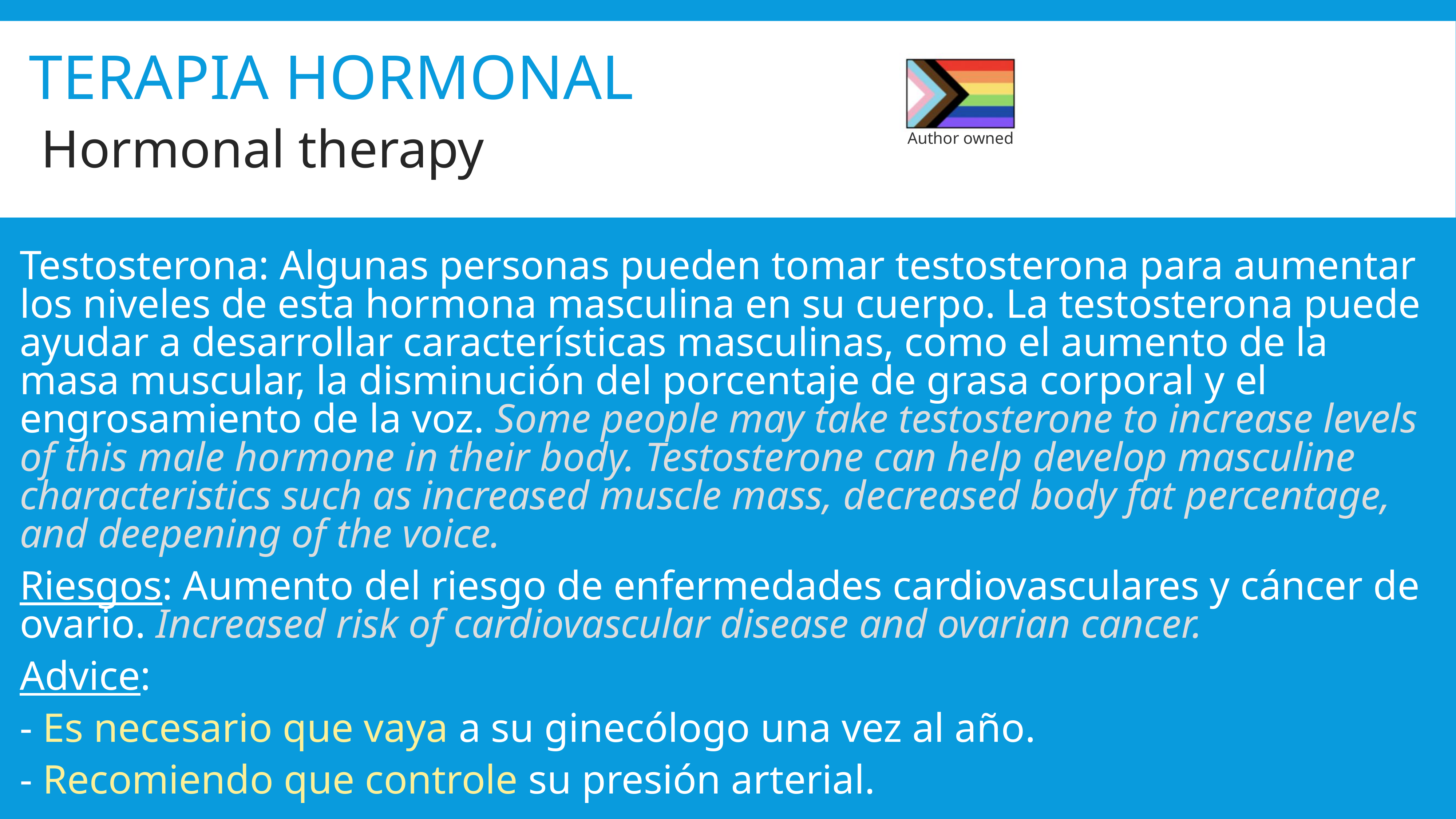

Terapia hormonal
Hormonal therapy
Author owned
Testosterona: Algunas personas pueden tomar testosterona para aumentar los niveles de esta hormona masculina en su cuerpo. La testosterona puede ayudar a desarrollar características masculinas, como el aumento de la masa muscular, la disminución del porcentaje de grasa corporal y el engrosamiento de la voz. Some people may take testosterone to increase levels of this male hormone in their body. Testosterone can help develop masculine characteristics such as increased muscle mass, decreased body fat percentage, and deepening of the voice.
Riesgos: Aumento del riesgo de enfermedades cardiovasculares y cáncer de ovario. Increased risk of cardiovascular disease and ovarian cancer.
Advice:
- Es necesario que vaya a su ginecólogo una vez al año.
- Recomiendo que controle su presión arterial.

## Slide 15
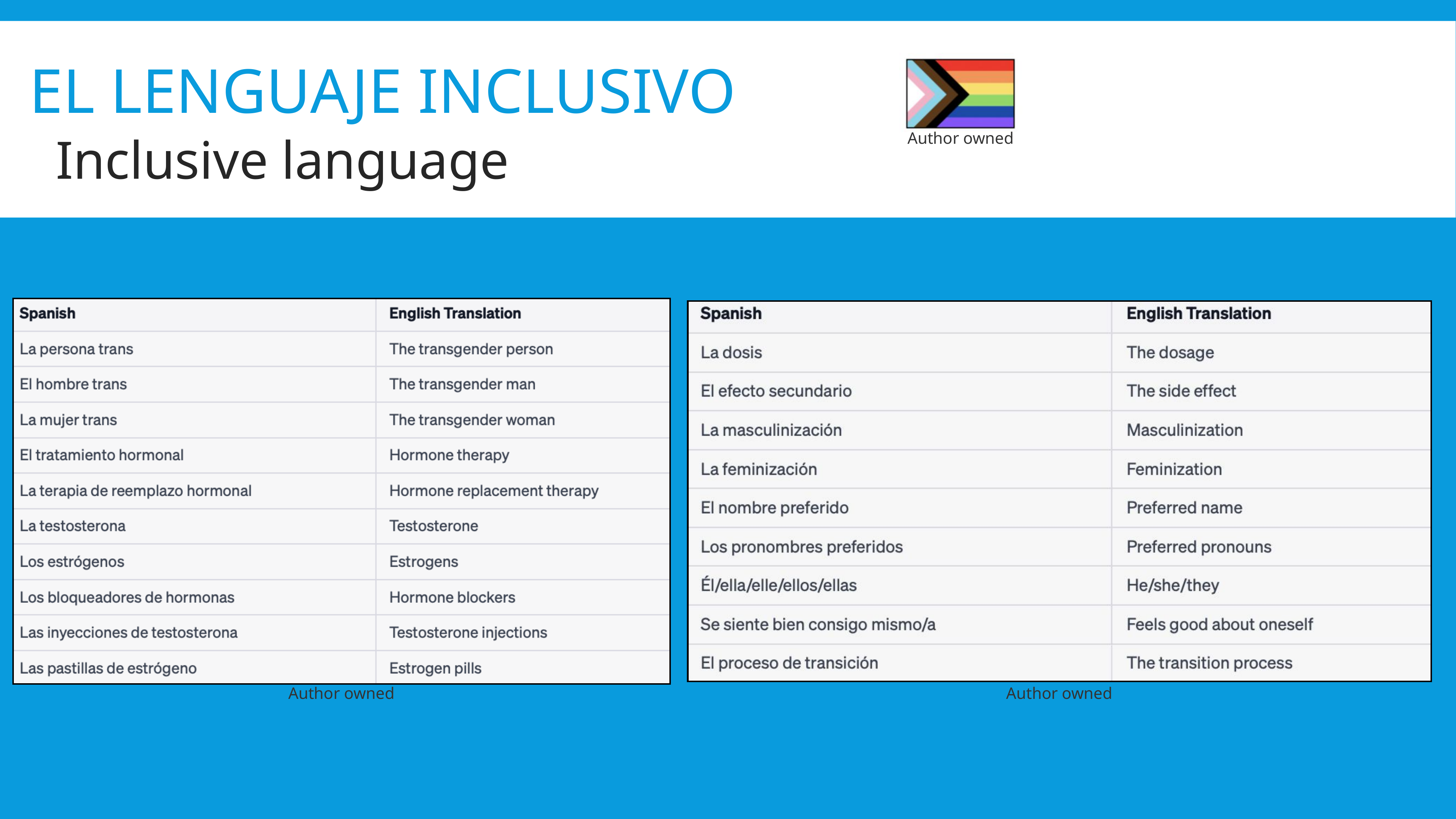

# El lenguaje inclusivo
Inclusive language
Author owned
Author owned
Author owned

## Slide 16
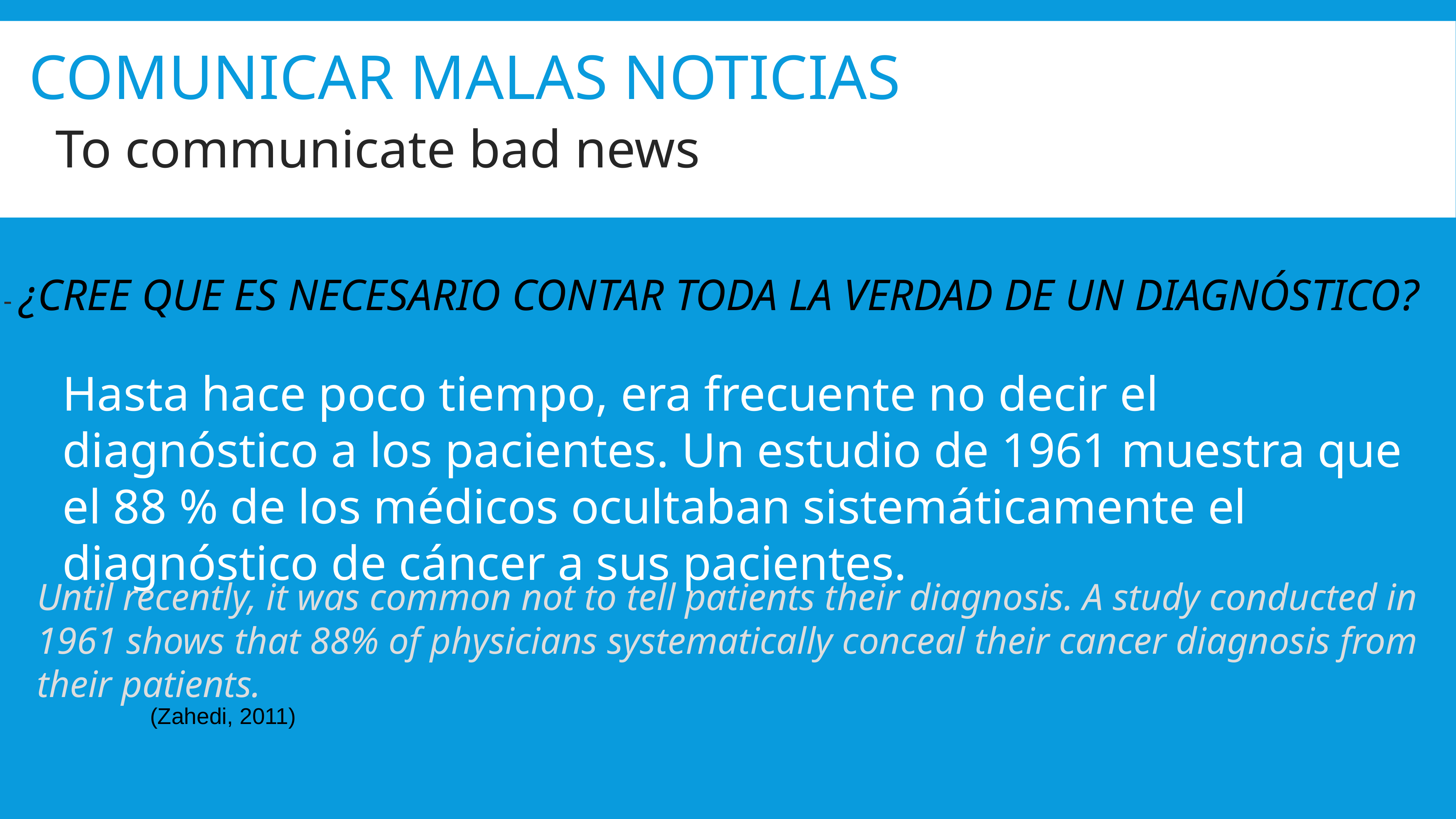

Comunicar malas noticias
To communicate bad news
- ¿CREE QUE ES NECESARIO CONTAR TODA LA VERDAD DE UN DIAGNÓSTICO?
Hasta hace poco tiempo, era frecuente no decir el diagnóstico a los pacientes. Un estudio de 1961 muestra que el 88 % de los médicos ocultaban sistemáticamente el diagnóstico de cáncer a sus pacientes.
Until recently, it was common not to tell patients their diagnosis. A study conducted in 1961 shows that 88% of physicians systematically conceal their cancer diagnosis from their patients.
(Zahedi, 2011)

## Slide 17
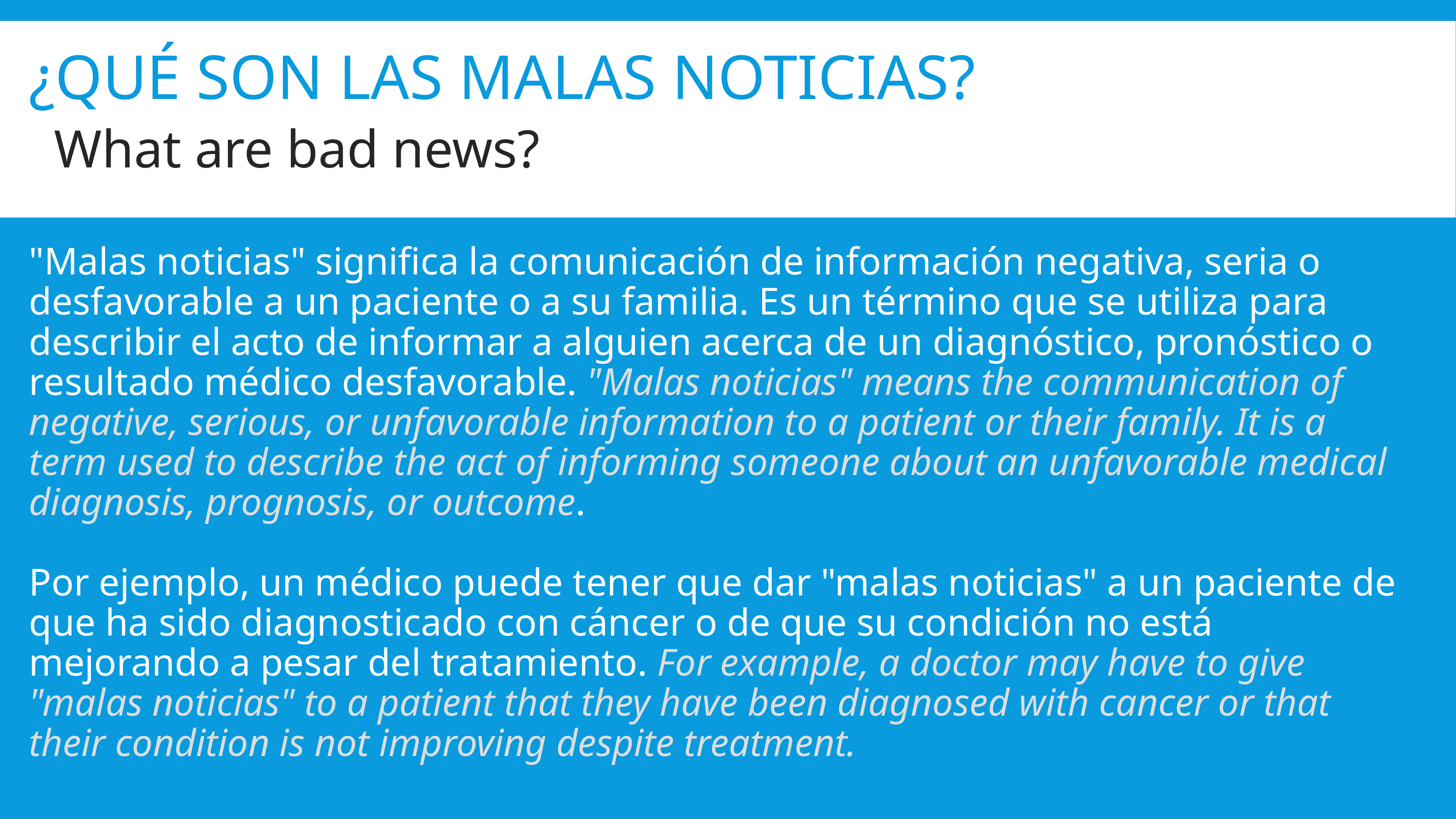

¿qué son las malas noticias?
What are bad news?
"Malas noticias" significa la comunicación de información negativa, seria o desfavorable a un paciente o a su familia. Es un término que se utiliza para describir el acto de informar a alguien acerca de un diagnóstico, pronóstico o resultado médico desfavorable. "Malas noticias" means the communication of negative, serious, or unfavorable information to a patient or their family. It is a term used to describe the act of informing someone about an unfavorable medical diagnosis, prognosis, or outcome.
Por ejemplo, un médico puede tener que dar "malas noticias" a un paciente de que ha sido diagnosticado con cáncer o de que su condición no está mejorando a pesar del tratamiento. For example, a doctor may have to give "malas noticias" to a patient that they have been diagnosed with cancer or that their condition is not improving despite treatment.

## Slide 18
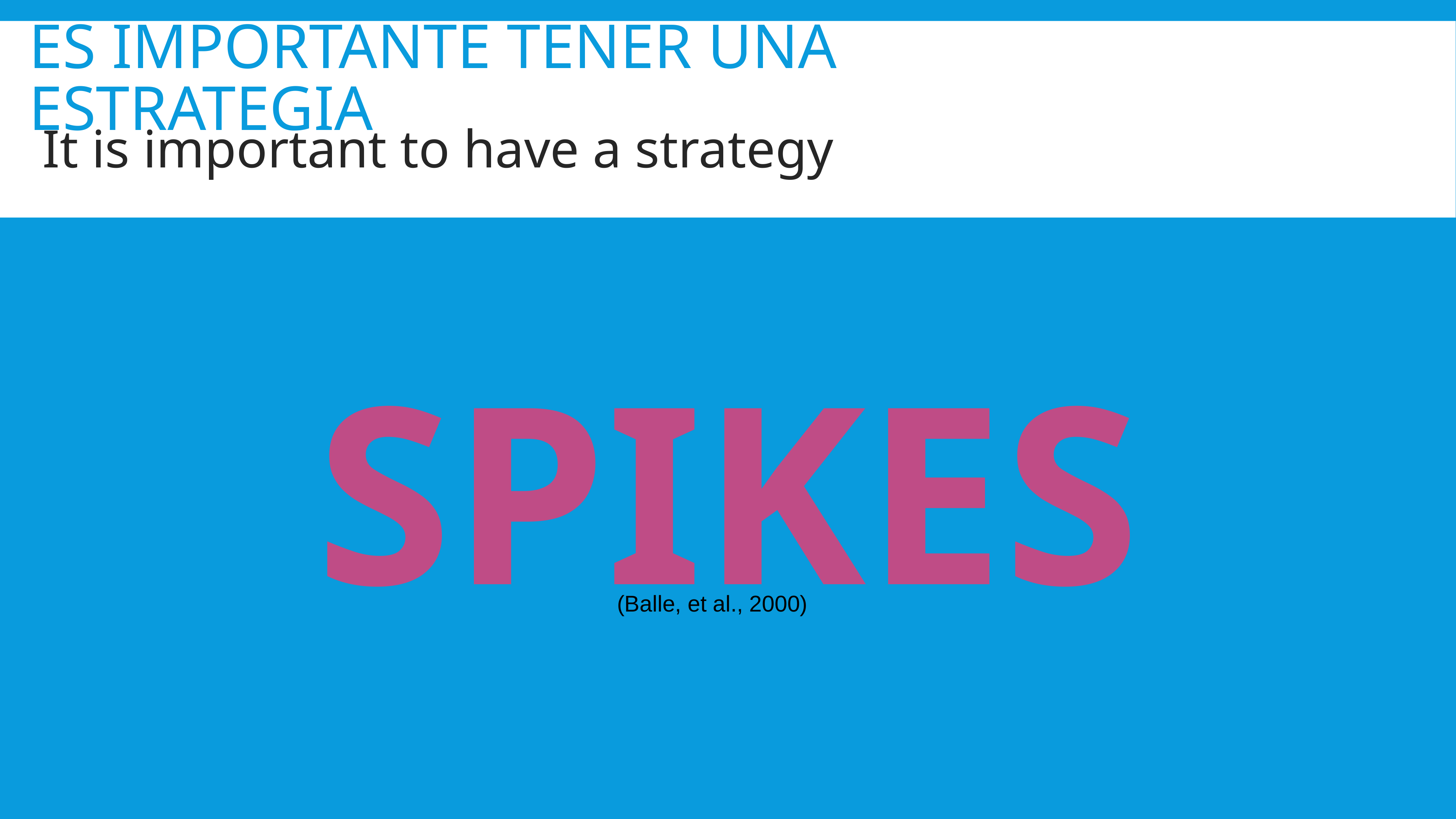

Es importante tener una estrategia
It is important to have a strategy
SPIKES
(Balle, et al., 2000)

## Slide 19
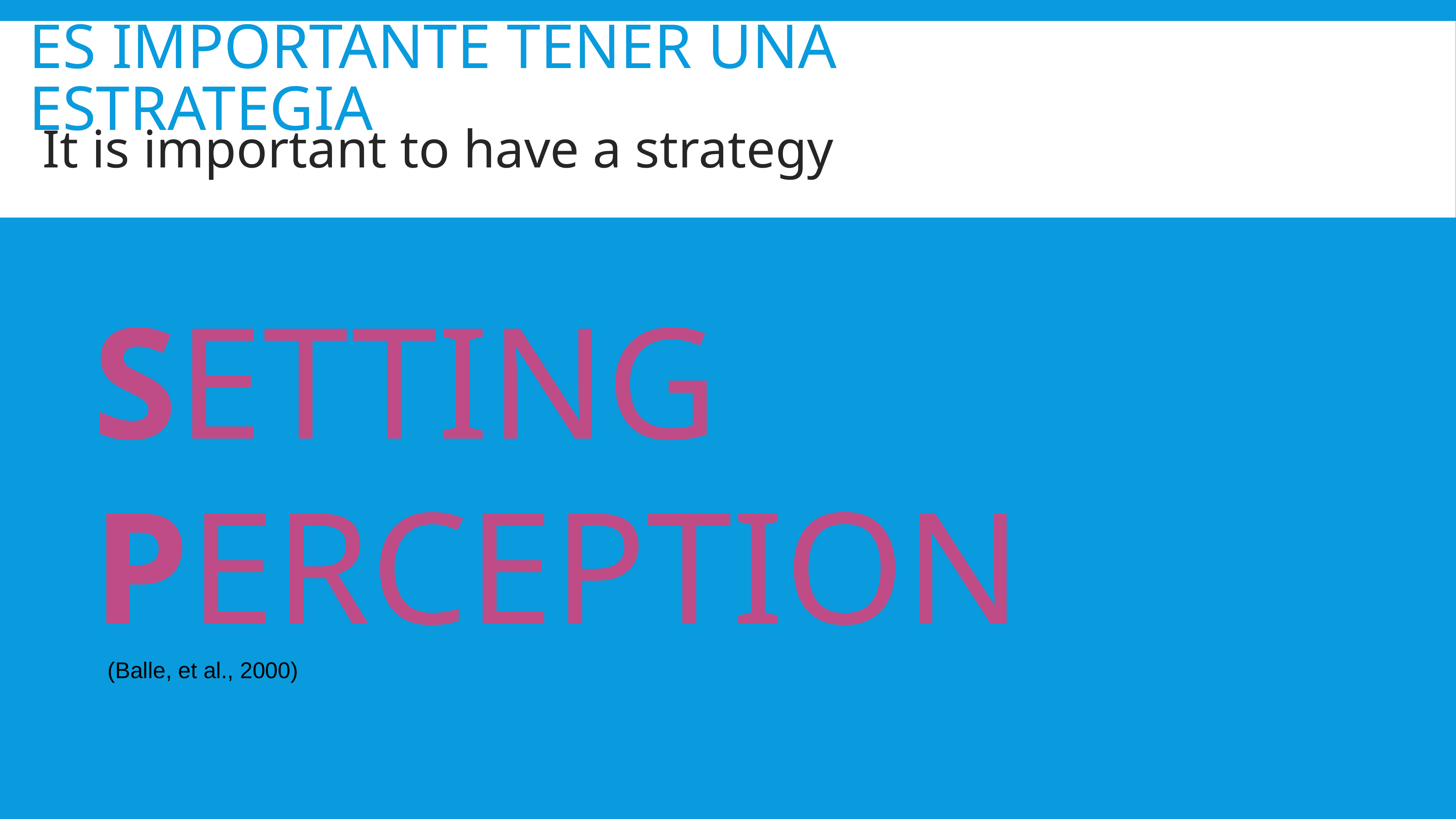

Es importante tener una estrategia
It is important to have a strategy
SETTING
PERCEPTION
(Balle, et al., 2000)

## Slide 20
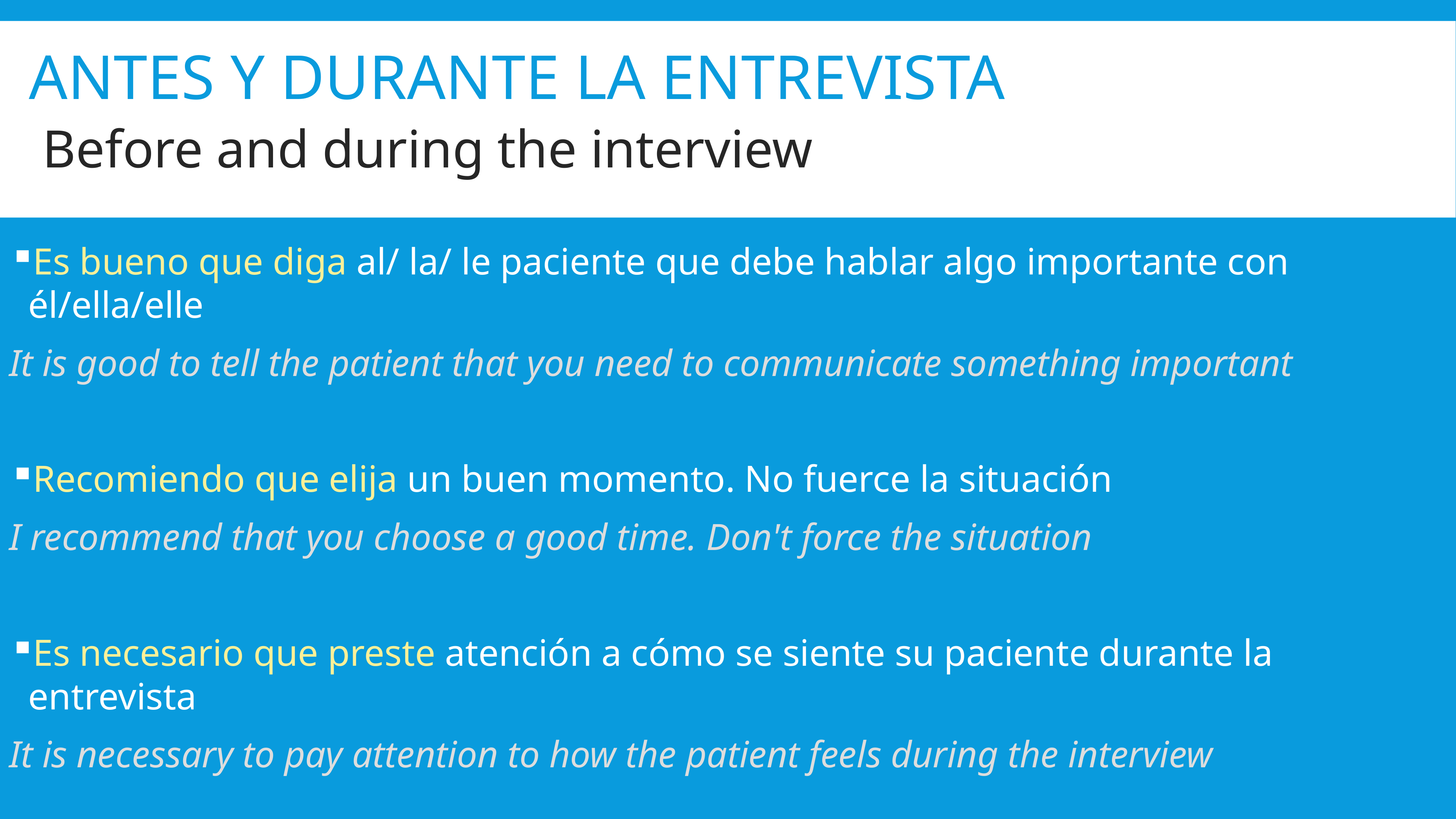

Antes y durante la entrevista
Before and during the interview
Es bueno que diga al/ la/ le paciente que debe hablar algo importante con él/ella/elle
It is good to tell the patient that you need to communicate something important
Recomiendo que elija un buen momento. No fuerce la situación
I recommend that you choose a good time. Don't force the situation
Es necesario que preste atención a cómo se siente su paciente durante la entrevista
It is necessary to pay attention to how the patient feels during the interview

## Slide 21
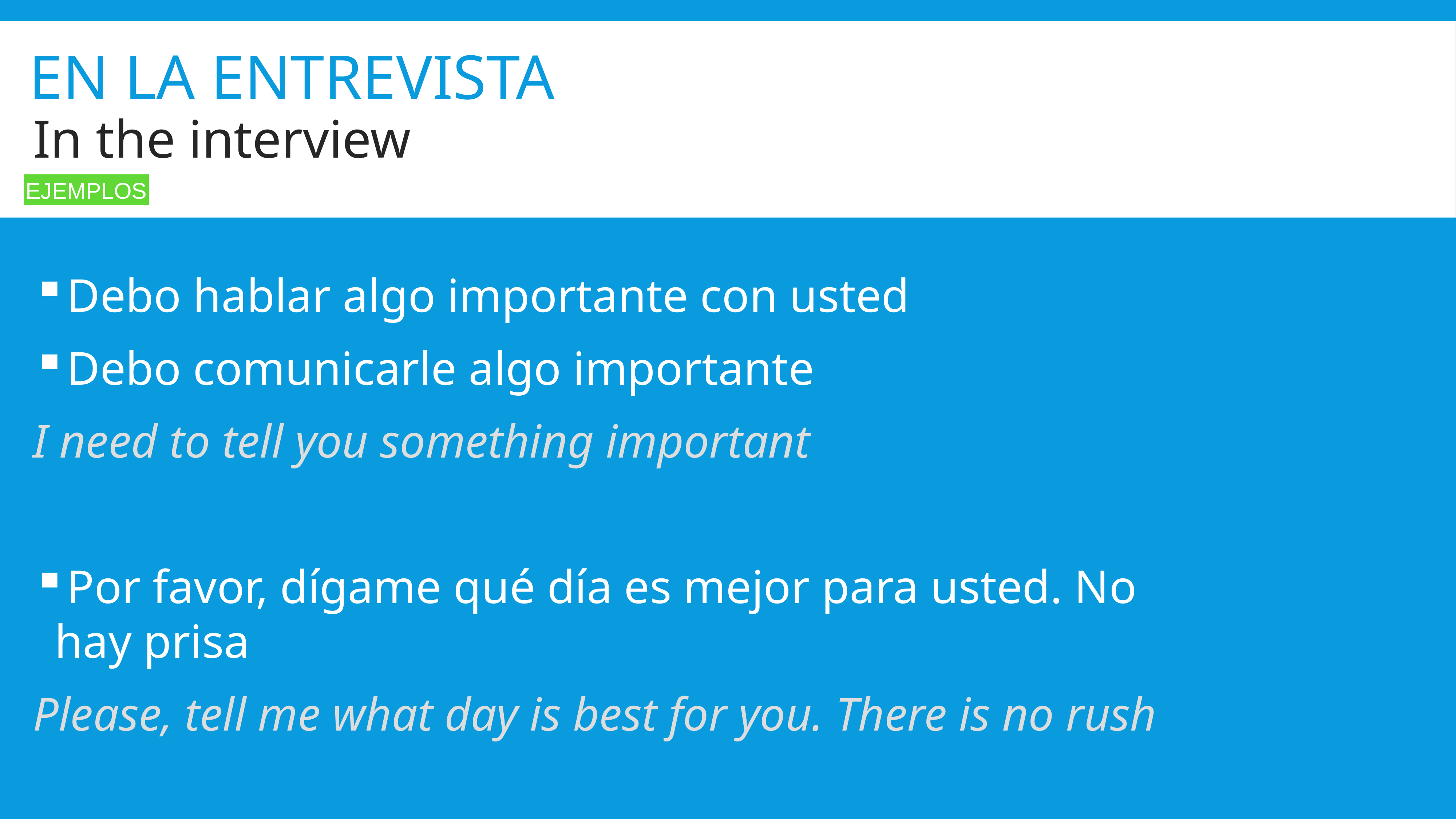

En la entrevista
In the interview
EJEMPLOS
Debo hablar algo importante con usted
Debo comunicarle algo importante
I need to tell you something important
Por favor, dígame qué día es mejor para usted. No hay prisa
Please, tell me what day is best for you. There is no rush

## Slide 22
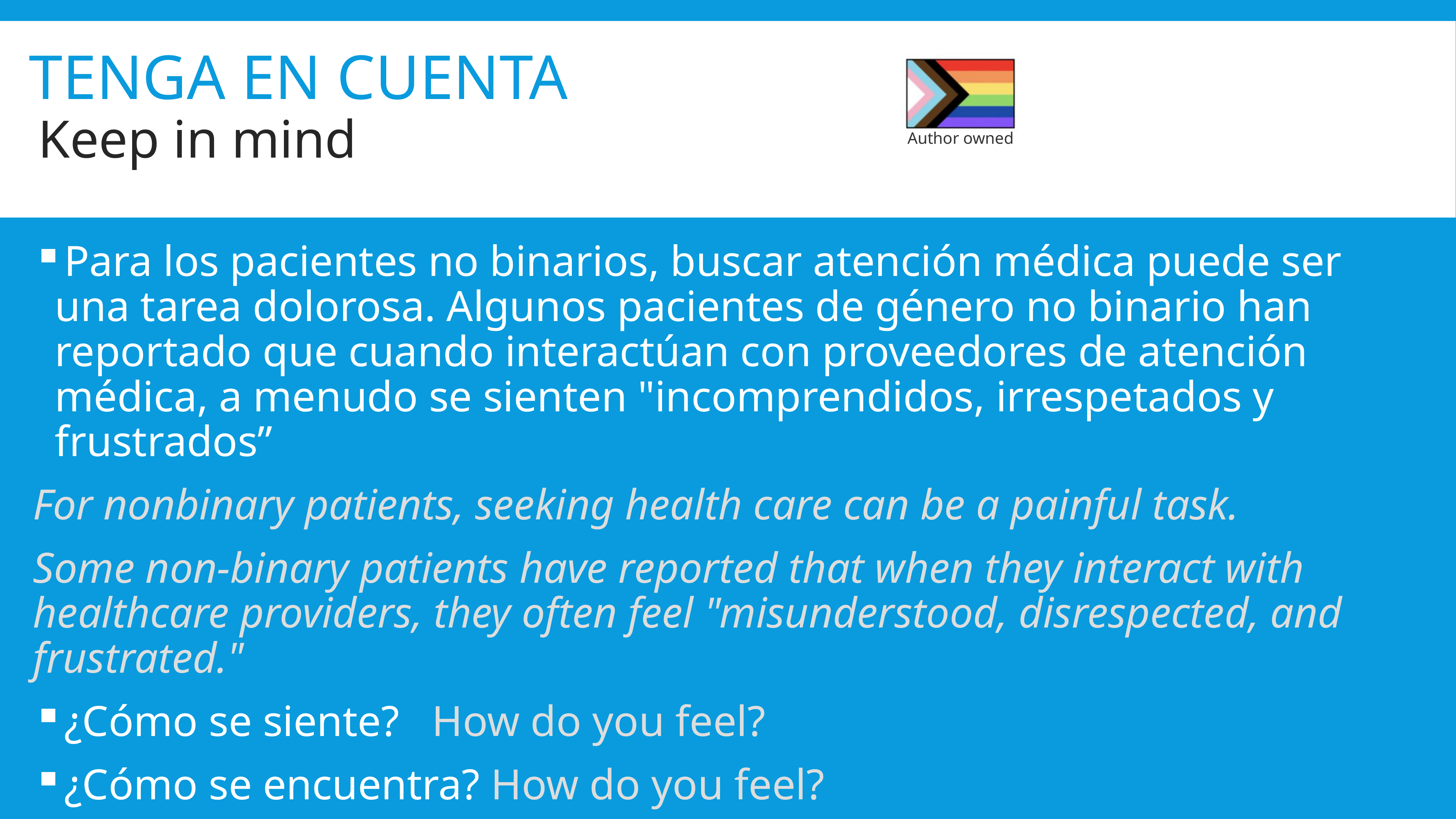

Tenga en cuenta
Keep in mind
Author owned
Para los pacientes no binarios, buscar atención médica puede ser una tarea dolorosa. Algunos pacientes de género no binario han reportado que cuando interactúan con proveedores de atención médica, a menudo se sienten "incomprendidos, irrespetados y frustrados”
For nonbinary patients, seeking health care can be a painful task.
Some non-binary patients have reported that when they interact with healthcare providers, they often feel "misunderstood, disrespected, and frustrated."
¿Cómo se siente? How do you feel?
¿Cómo se encuentra? How do you feel?

## Slide 23
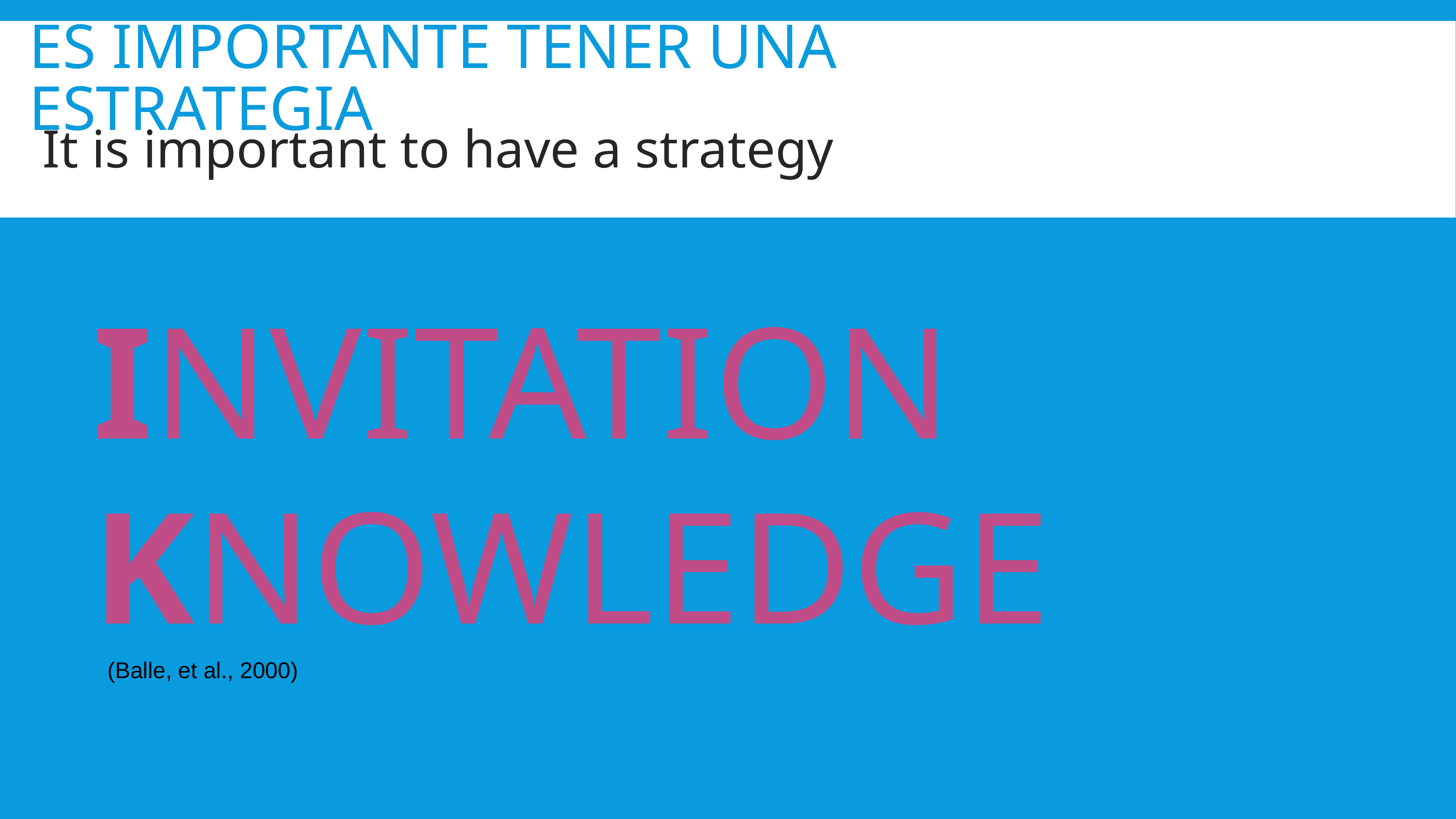

Es importante tener una estrategia
It is important to have a strategy
INVITATION
KNOWLEDGE
(Balle, et al., 2000)

## Slide 24
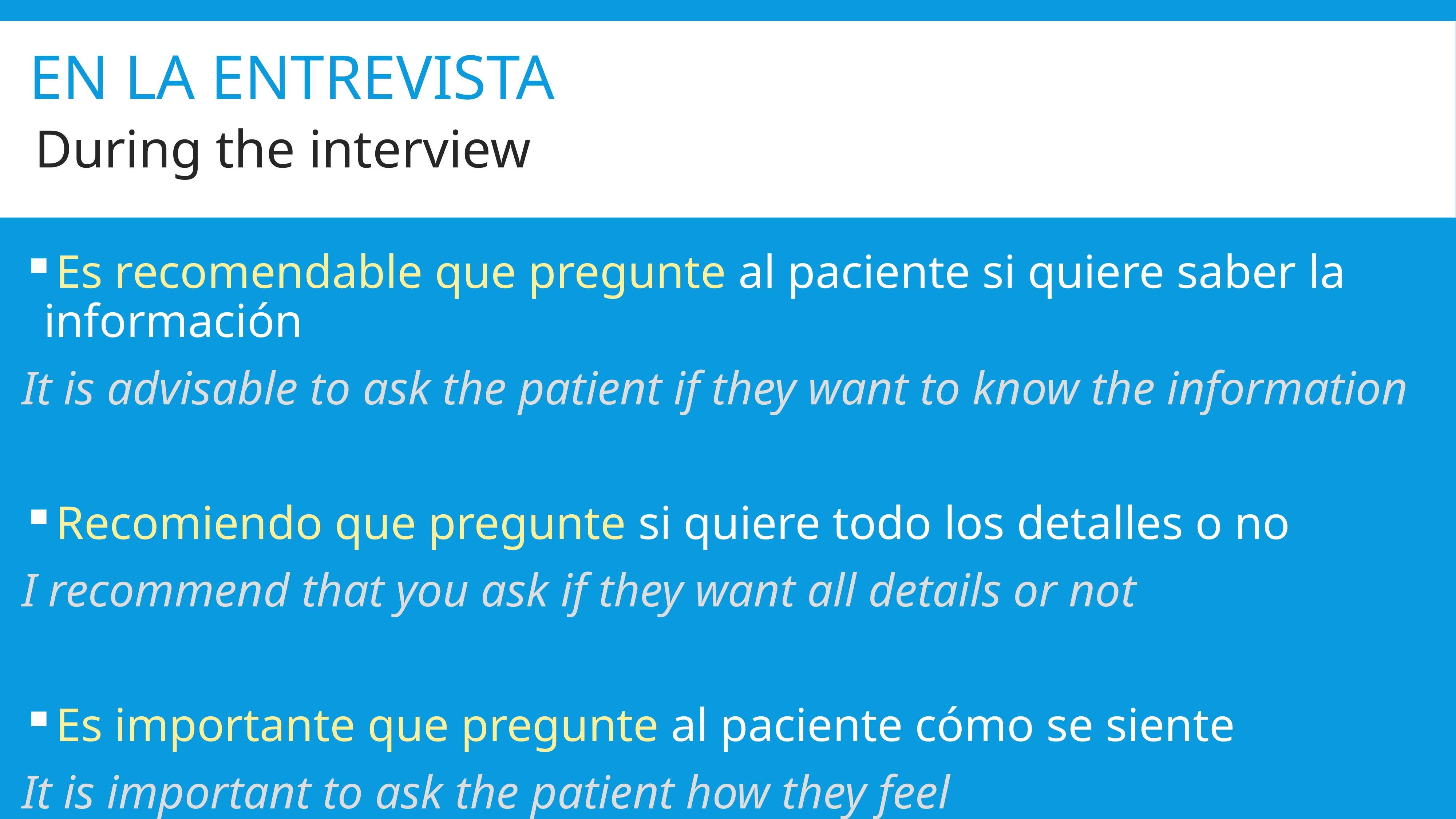

En la entrevista
During the interview
Es recomendable que pregunte al paciente si quiere saber la información
It is advisable to ask the patient if they want to know the information
Recomiendo que pregunte si quiere todo los detalles o no
I recommend that you ask if they want all details or not
Es importante que pregunte al paciente cómo se siente
It is important to ask the patient how they feel

## Slide 25
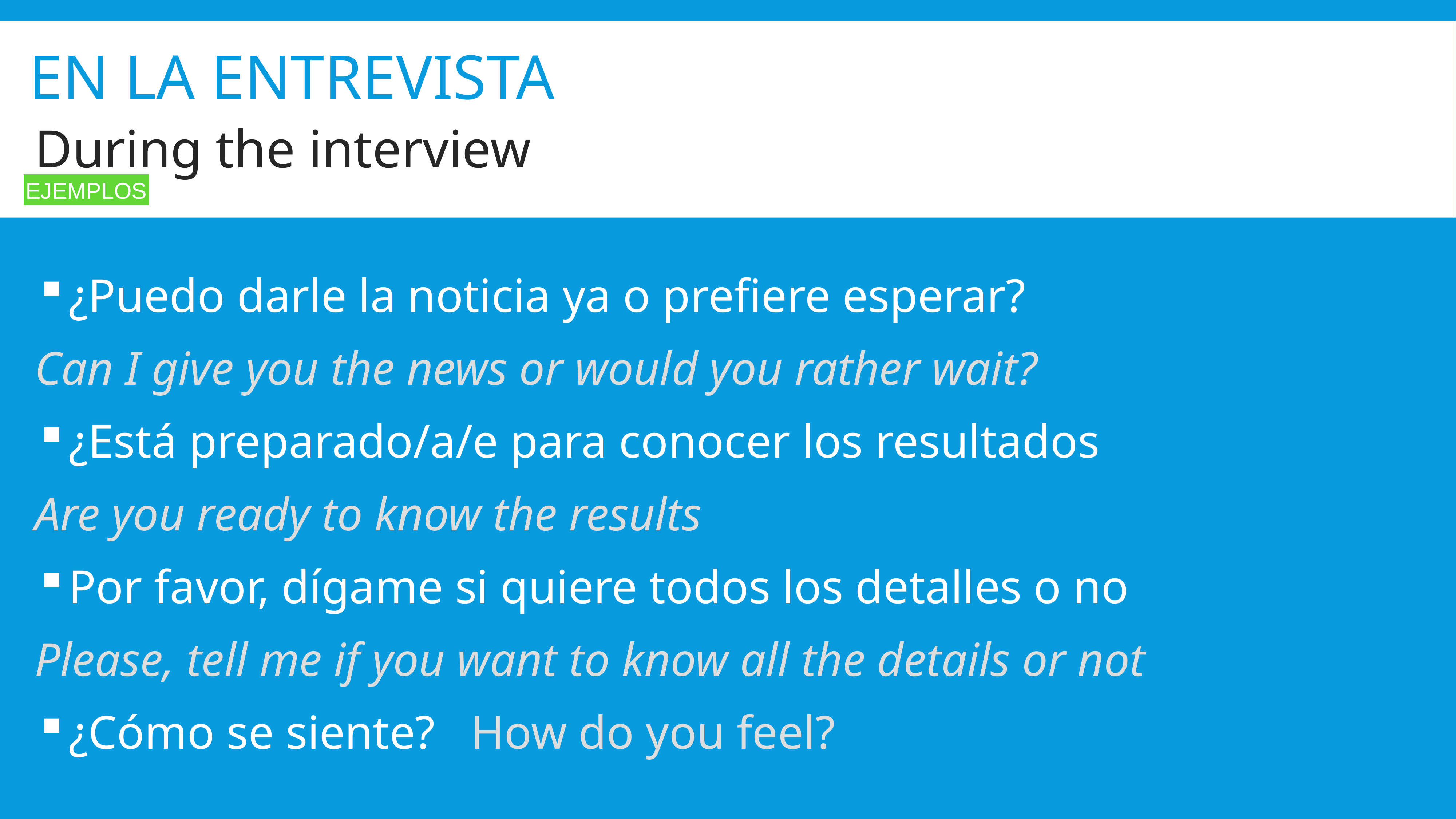

En la entrevista
During the interview
EJEMPLOS
¿Puedo darle la noticia ya o prefiere esperar?
Can I give you the news or would you rather wait?
¿Está preparado/a/e para conocer los resultados
Are you ready to know the results
Por favor, dígame si quiere todos los detalles o no
Please, tell me if you want to know all the details or not
¿Cómo se siente? How do you feel?

## Slide 26
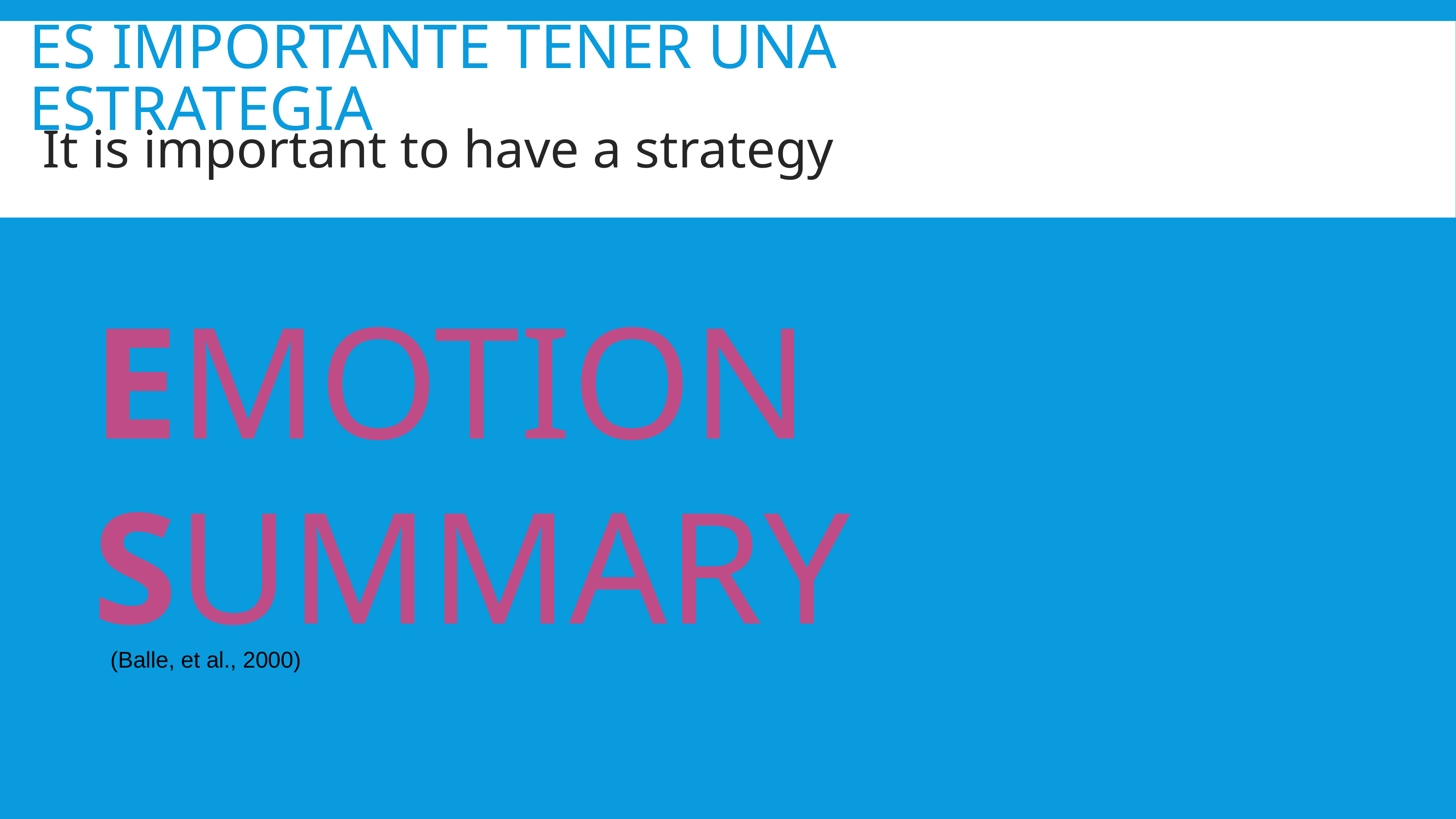

Es importante tener una estrategia
It is important to have a strategy
EMOTION
SUMMARY
(Balle, et al., 2000)

## Slide 27
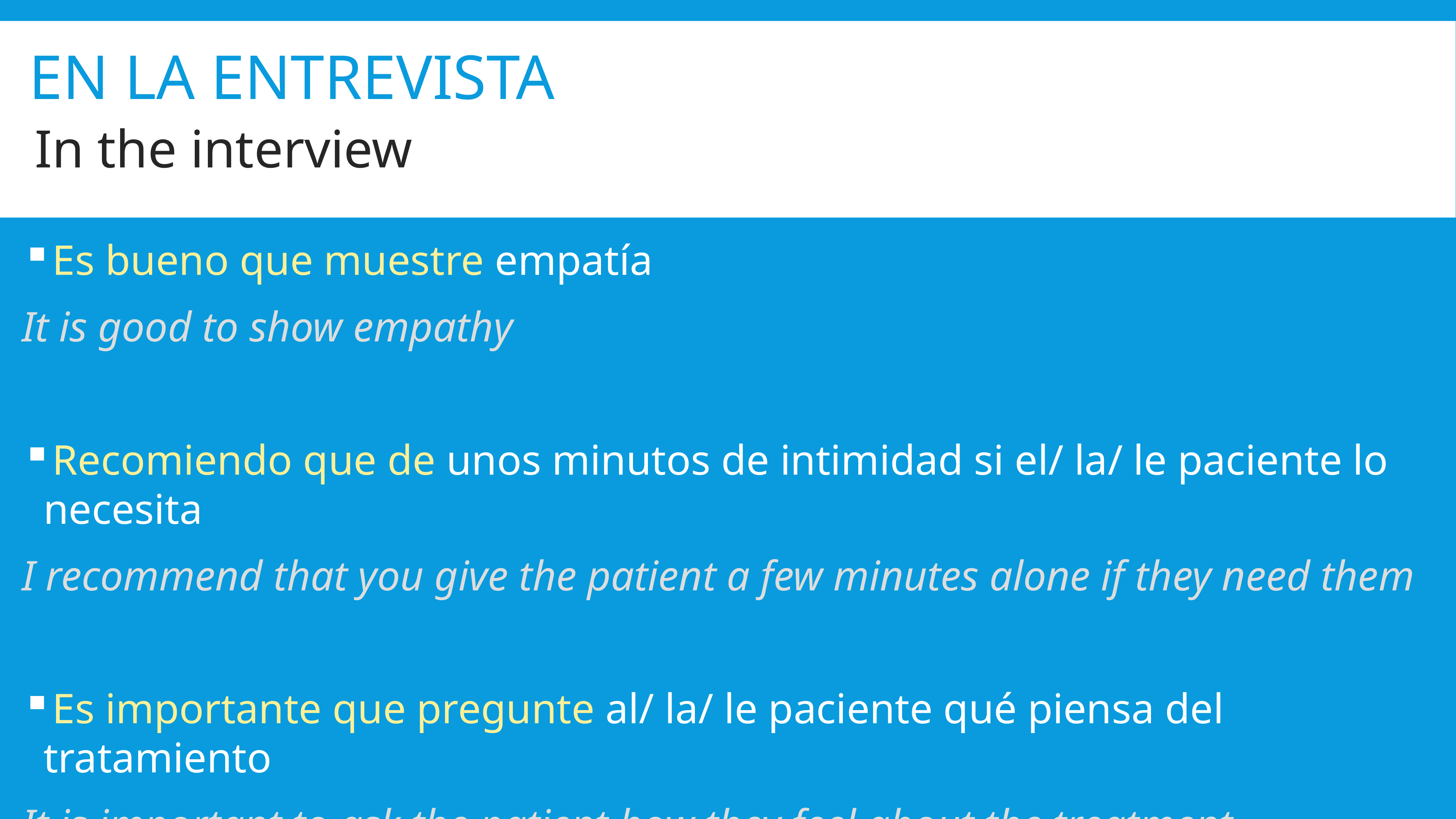

En la entrevista
In the interview
Es bueno que muestre empatía
It is good to show empathy
Recomiendo que de unos minutos de intimidad si el/ la/ le paciente lo necesita
I recommend that you give the patient a few minutes alone if they need them
Es importante que pregunte al/ la/ le paciente qué piensa del tratamiento
It is important to ask the patient how they feel about the treatment

## Slide 28
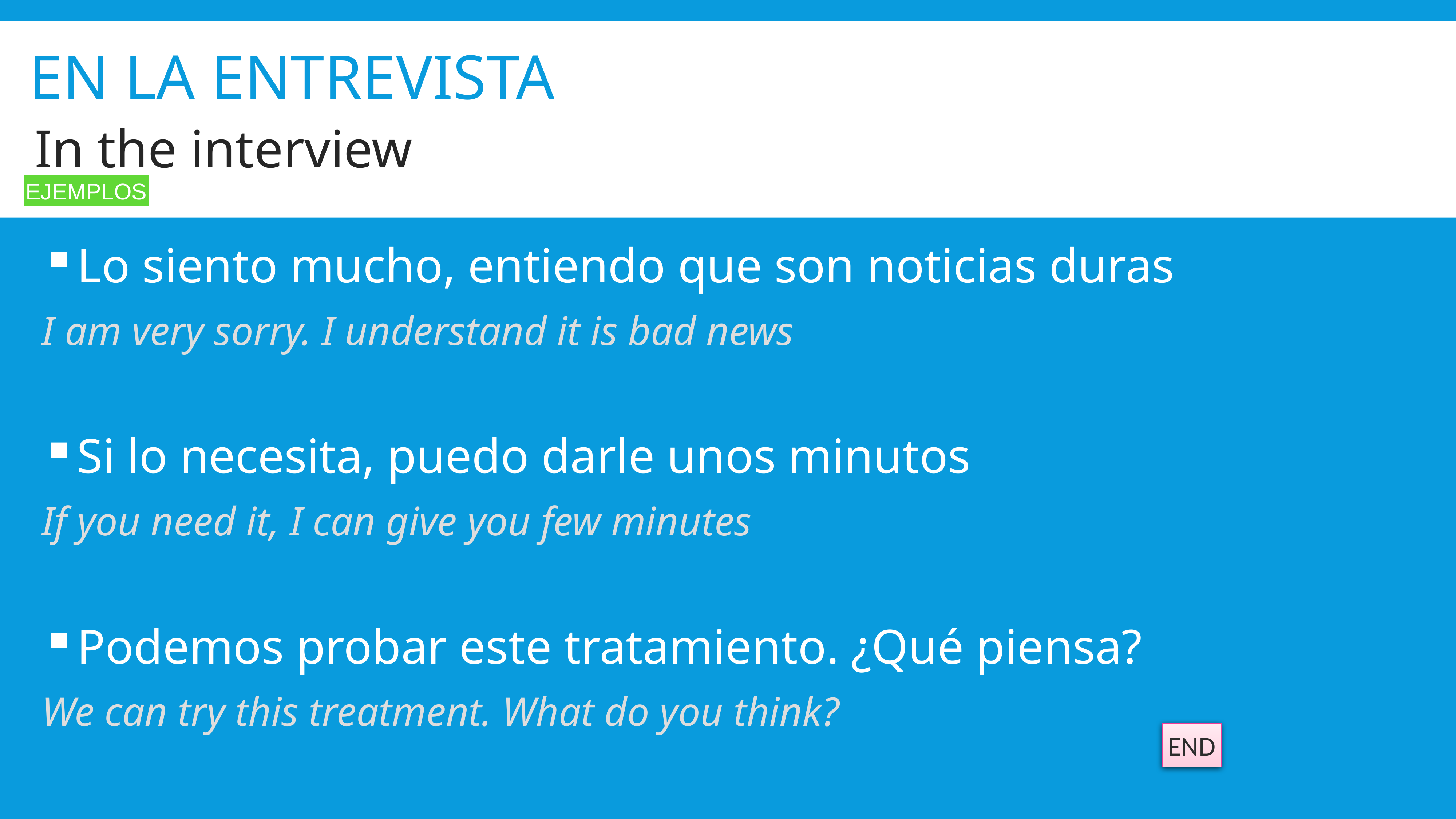

En la entrevista
In the interview
EJEMPLOS
Lo siento mucho, entiendo que son noticias duras
I am very sorry. I understand it is bad news
Si lo necesita, puedo darle unos minutos
If you need it, I can give you few minutes
Podemos probar este tratamiento. ¿Qué piensa?
We can try this treatment. What do you think?
END

## Slide 29
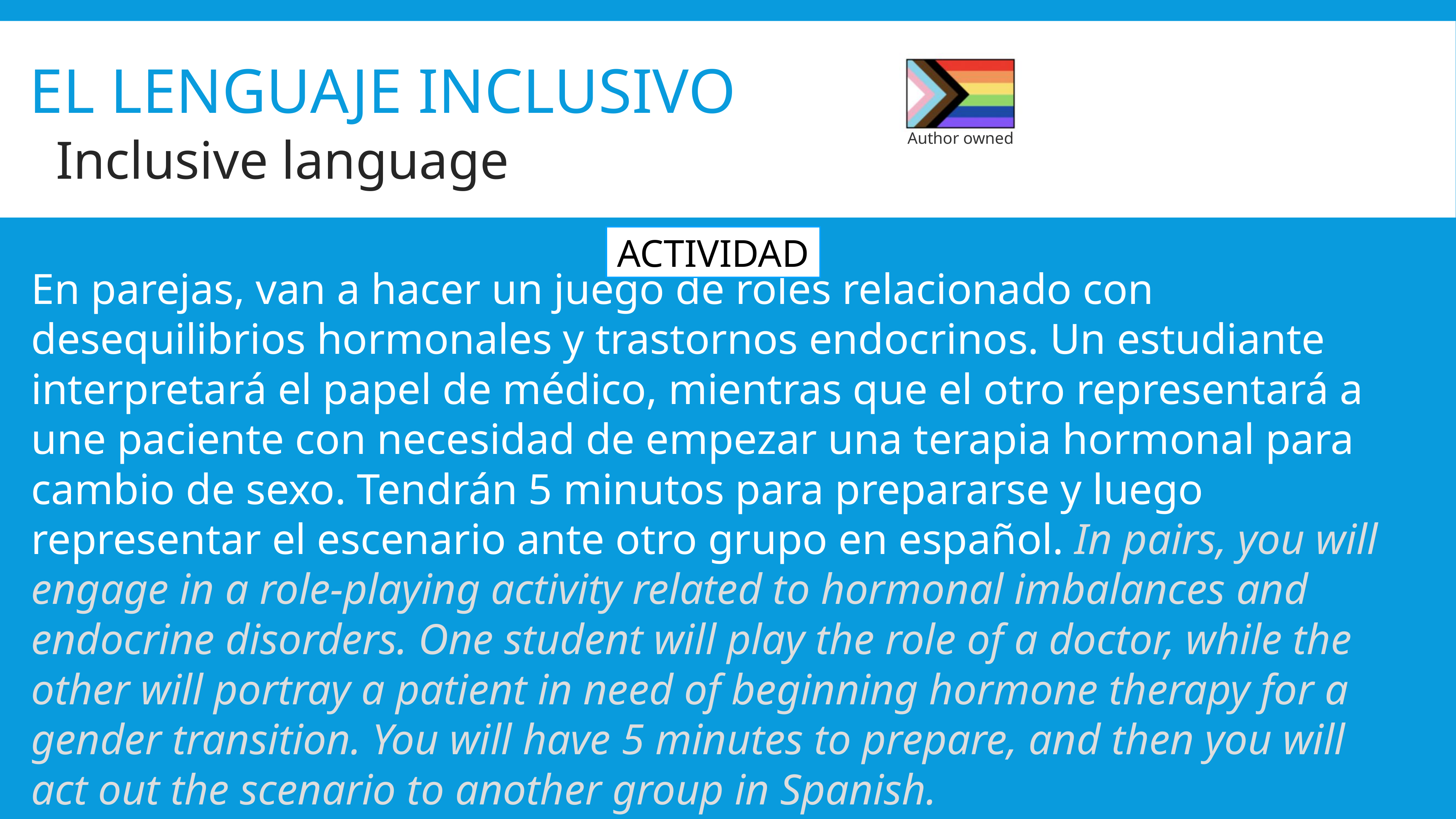

# El lenguaje inclusivo
Inclusive language
Author owned
ACTIVIDAD
En parejas, van a hacer un juego de roles relacionado con desequilibrios hormonales y trastornos endocrinos. Un estudiante interpretará el papel de médico, mientras que el otro representará a une paciente con necesidad de empezar una terapia hormonal para cambio de sexo. Tendrán 5 minutos para prepararse y luego representar el escenario ante otro grupo en español. In pairs, you will engage in a role-playing activity related to hormonal imbalances and endocrine disorders. One student will play the role of a doctor, while the other will portray a patient in need of beginning hormone therapy for a gender transition. You will have 5 minutes to prepare, and then you will act out the scenario to another group in Spanish.

## Slide 30
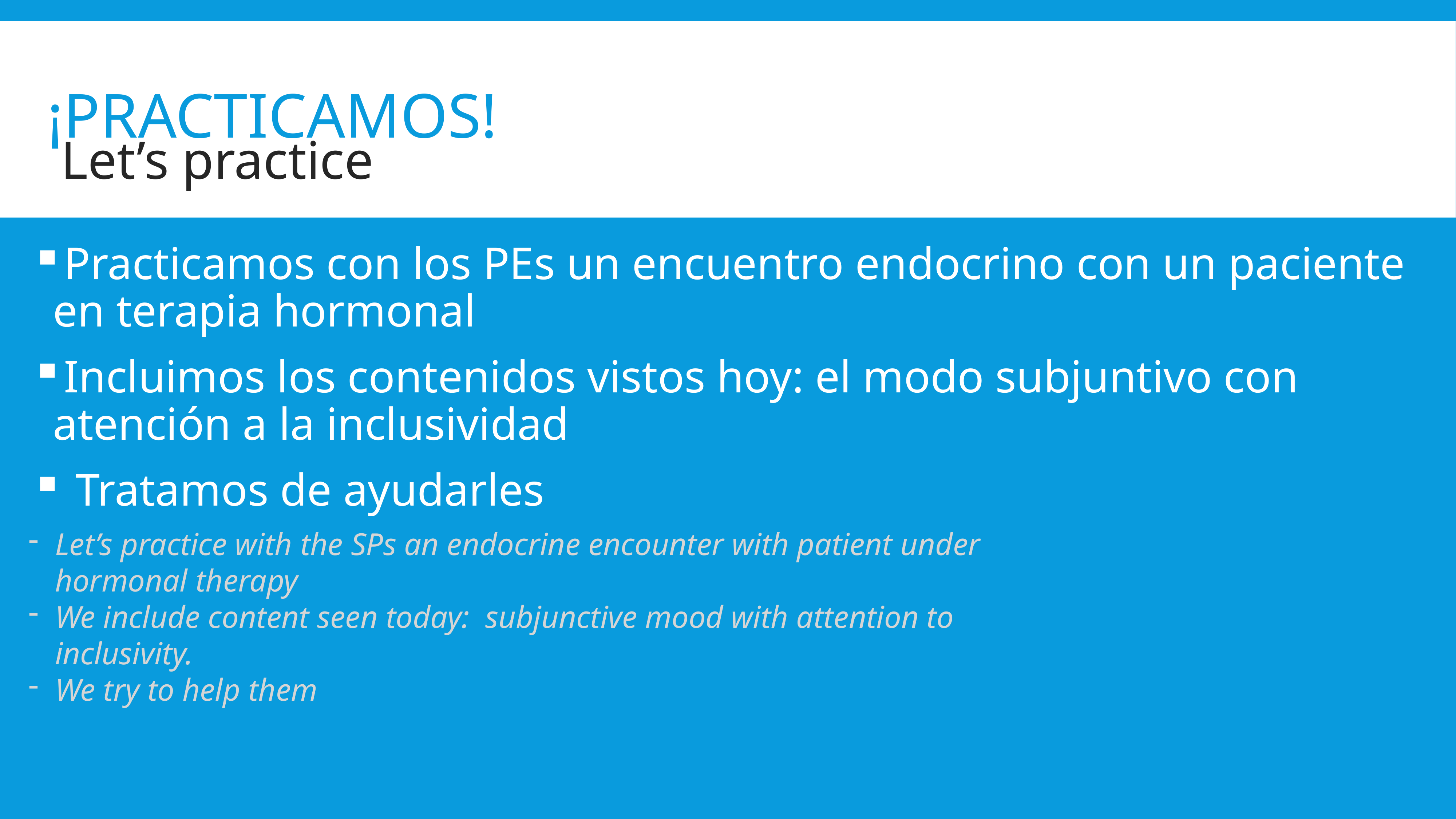

# ¡Practicamos!
Let’s practice
Practicamos con los PEs un encuentro endocrino con un paciente en terapia hormonal
Incluimos los contenidos vistos hoy: el modo subjuntivo con atención a la inclusividad
 Tratamos de ayudarles
Let’s practice with the SPs an endocrine encounter with patient under hormonal therapy
We include content seen today: subjunctive mood with attention to inclusivity.
We try to help them

## Slide 31
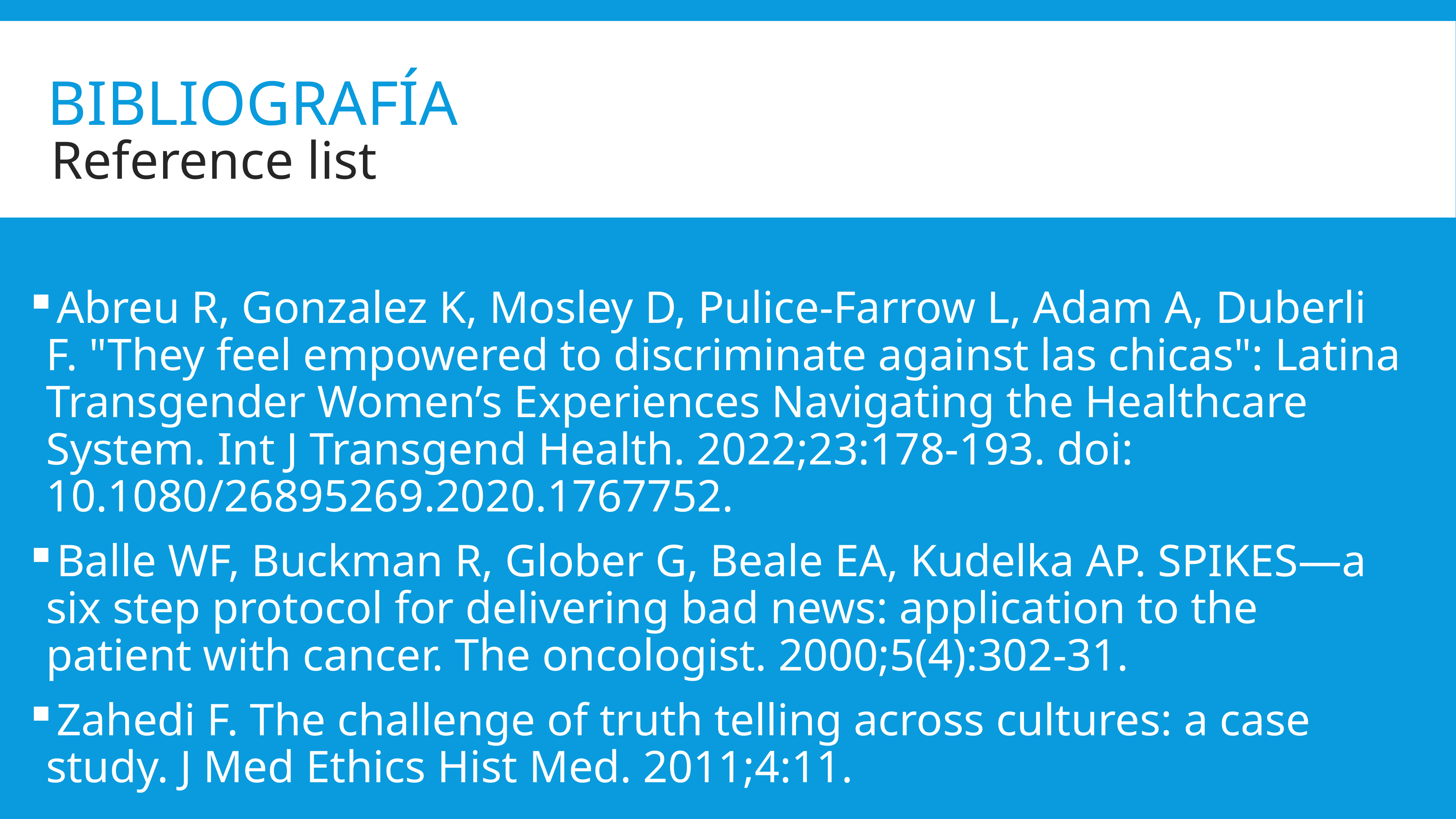

# BIBLIOGRAFía
Reference list
Abreu R, Gonzalez K, Mosley D, Pulice-Farrow L, Adam A, Duberli F. "They feel empowered to discriminate against las chicas": Latina Transgender Women’s Experiences Navigating the Healthcare System. Int J Transgend Health. 2022;23:178-193. doi: 10.1080/26895269.2020.1767752.
Balle WF, Buckman R, Glober G, Beale EA, Kudelka AP. SPIKES—a six step protocol for delivering bad news: application to the patient with cancer. The oncologist. 2000;5(4):302-31.
Zahedi F. The challenge of truth telling across cultures: a case study. J Med Ethics Hist Med. 2011;4:11.
