# Supplementary material for: Integrating Gender-Affirming Care in a Medical Spanish Endocrine System Curriculum
Source: MedEdPORTAL. 2024 Oct 23;20:11456. doi: 10.15766/mep_2374-8265.11456 (PMC11496385; doi:10.15766/mep_2374-8265.11456)
Supplement: Supplementary file 1 — Facilitator Guide.docxLesson 1 Presentation.pptxLesson 2 Presentation.pptxLesson 3 Presentation.pptxLesson 1 Clinical Endocrine Checklist.docxLesson 2 Clinical Endocrine Checklist.docxLesson 3 Clinical Endocrine Checklist.docxLesson 1 SP Case.docxLesson 2 SP Case.docxLesson 3 SP Case.docxPre-Post Confidence Survey.docxPre-Post Spanish Endocrine Test.docxOSCE SP Diabetic Case.docxOSCE Door Note.docxOSCE Clinical Checklist Diabetic Encounter.docxOSCE Language Rubric for Diabetic Encounter.docx [file mep_2374-8265.11456-s001.zip › E. Lesson 1 Clinical Endocrine Checklist.docx]

**Appendix E.** Clinical Endocrine Checklist Lesson 1

# Sistema Endocrino I: Diabetes

**Setting the Stage**

I. Greet the patient and introduce yourself, verify identifying data, including preferred name and pronouns, ensure privacy and comfort, set the agenda, and ask for permission to proceed.

# Inclusive Healthcare Considerations

1. Diverse Patient Preferences and Needs
   1. Are there any specific cultural or religious practices, beliefs, or traditions that are important for me to be aware of when discussing your healthcare?
      1. ¿Existen prácticas culturales o religiosas específicas, creencias o tradiciones que sean importantes que yo conozca cuando hablemos de su atención médica?
   2. Are there any particular concerns or considerations related to your gender-affirming care or any medications you are taking as part of your gender-affirming journey that we should take into account?
      1. ¿Tiene preocupaciones o consideraciones particulares relacionadas con su atención de afirmación de género o con los medicamentos que está tomando como parte de su proceso de afirmación de género que debamos tener en cuenta?
   3. Do you have any accessibility needs or requirements related to your disability, mobility, or communication that we should be aware of during your healthcare appointments?
      1. ¿Tiene necesidades o requisitos de accesibilidad relacionados con su discapacidad, movilidad o comunicación que debamos conocer durante sus citas médicas?
   4. Are there any specific ways you'd like me to address or refer to your body or health concerns that align with your gender identity and affirm your identity and experiences?
      1. ¿Hay alguna manera específica en la que le gustaría que me refiera o hable sobre su cuerpo o preocupaciones de salud que esté alineada con su identidad de género y afirme su identidad y experiencias?

# History

1. Elicit chief complaint and SOCRATES (S: Site - Where is the pain located?, O: Onset - When did the pain start, and was it sudden or gradual?, C: Character - What is the pain like? Is it stabbing, burning, throbbing, etc.?, R: Radiation - Does the pain radiate or spread to any other areas?, A: Associations - Are there any other signs or symptoms associated with the pain?, Time course - Does the pain follow any pattern? How long does it last? Does it come and go?, E: Exacerbating/Relieving factors - What makes the pain better or worse?, S: Severity - How bad is the pain on a scale of 1 to 10, or how does it impact the ability to carry out activities?)
   1. What brings you in today?
      1. ¿Qué le trae a la clínica hoy?
   2. Please tell me more.
      1. Por favor, dígame más.
   3. What symptoms do you have?
      1. ¿Qué síntomas presenta?
   4. When did they start?
      1. ¿Cuándo empezaron?
   5. Do you have discomfort?
      1. ¿Tiene incomodidad?
   6. Does the pain spread anywhere?
      1. ¿Se extiende el dolor a otras partes del cuerpo?
   7. Does anything else feel unusual? Do you have any other symptoms?
      1. ¿Se siente algo más anormal? ¿Tiene algún otro síntoma?
   8. Is it constant, or does it come and go?
      1. ¿Es constante, o va y viene?
   9. What makes the discomfort better? Worse?
      1. ¿Qué lo mejora? ¿Qué lo empeora?
   10. On a scale of 1 to 10, how much does it hurt?
       1. En una escala del uno al diez, ¿cuánto le duele?
2. Case-specific questions
   1. How long have you been experiencing these symptoms?
      1. ¿ Cuánto tiempo lleva sintiendo estos síntomas?
   2. Have you experienced any episodes of low blood sugar (hypoglycemia) or high blood sugar (hyperglycemia)?
      1. ¿Ha experimentado episodios de bajo nivel de azúcar en la sangre (hipoglucemia) o alto nivel de azúcar en la sangre (hiperglucemia)?
   3. Have you made any changes in your diet, exercise routine, or lifestyle to manage your diabetes?
      1. ¿Ha realizado cambios en su dieta, rutina de ejercicio o estilo de vida para manejar su diabetes?
   4. Have you had any recent infections, slow healing of wounds, or frequent infections, which can be related to diabetes?
      1. ¿Ha tenido infecciones recientes, cicatrización lenta de heridas o infecciones frecuentes, que puedan estar relacionadas con la diabetes?
   5. Have you noticed any changes in your vision or eye health?
      1. ¿Ha notado cambios en su visión o salud ocular?
   6. Have you been experiencing any symptoms such as increased thirst, frequent urination, blurred vision, or unexplained weight loss?
      1. ¿Ha experimentado síntomas como sed excesiva, micción frecuente, visión borrosa o pérdida de peso inexplicable?
   7. Have you noticed any new or worsening symptoms such as numbness or tingling in your extremities, slow healing wounds, or recurring infections?
      1. ¿Ha notado algún síntoma nuevo o empeoramiento de síntomas como entumecimiento o hormigueo en sus extremidades, heridas que sanan lentamente o infecciones recurrentes?
   8. Do you have a family history of diabetes or any other risk factors such as obesity, high blood pressure, or high cholesterol?
      1. ¿Tiene antecedentes familiares de diabetes u otros factores de riesgo como obesidad, hipertensión arterial o colesterol alto?
   9. Have you been following your healthcare provider's recommendations for managing your diabetes, including diet, exercise, medication, and regular check-ups?
      1. ¿Ha estado siguiendo las recomendaciones de su proveedor de atención médica para el manejo de su diabetes, incluyendo la dieta, el ejercicio, la medicación y las revisiones regulares?
3. **Lifestyle**
   1. Do you work out often?
      1. ¿Hace deporte a menudo?
   2. How is your diet normally?
      1. ¿Cómo es su dieta normalmente?
   3. Do you consider yourself physically active or sedentary?
      1. ¿Se considera una persona fisicamente activa o sedentaria?
4. **If pertinent to the condition**
   1. Do you have a partner?
      1. ¿Tiene pareja?
   2. Are you active sexually?
      1. ¿Está activo/a/e sexualmente?
   3. Do you have more than one sexual partners?
      1. ¿Tiene más de una pareja sexual?
   4. Does your partner have more sexual partners?
      1. ¿Tu pareja tiene más parejas sexuales?
