# Supplementary material for: Integrating Gender-Affirming Care in a Medical Spanish Endocrine System Curriculum
Source: MedEdPORTAL. 2024 Oct 23;20:11456. doi: 10.15766/mep_2374-8265.11456 (PMC11496385; doi:10.15766/mep_2374-8265.11456)
Supplement: Supplementary file 1 — Facilitator Guide.docxLesson 1 Presentation.pptxLesson 2 Presentation.pptxLesson 3 Presentation.pptxLesson 1 Clinical Endocrine Checklist.docxLesson 2 Clinical Endocrine Checklist.docxLesson 3 Clinical Endocrine Checklist.docxLesson 1 SP Case.docxLesson 2 SP Case.docxLesson 3 SP Case.docxPre-Post Confidence Survey.docxPre-Post Spanish Endocrine Test.docxOSCE SP Diabetic Case.docxOSCE Door Note.docxOSCE Clinical Checklist Diabetic Encounter.docxOSCE Language Rubric for Diabetic Encounter.docx [file mep_2374-8265.11456-s001.zip › F. Lesson 2 Clinical Endocrine Checklist.docx]

Sistema Endocrino II: Hipertiroidismo y Hipotiroidismo

**Appendix F.** Clinical Endocrine Checklist Lesson 2

##### Setting the Stage

I. Greet the patient and introduce yourself, verify identifying data, including preferred name and pronouns, ensure privacy and comfort, set the agenda, and ask for permission to proceed.

# **Inclusive Healthcare Considerations**

1. Diverse Patient Preferences and Needs
   1. Are there any specific cultural or religious practices, beliefs, or traditions that are important for me to be aware of when discussing your healthcare?
      1. ¿Existen prácticas culturales o religiosas específicas, creencias o tradiciones que sean importantes que yo conozca cuando hablemos de su atención médica?
   2. Are there any particular concerns or considerations related to your gender-affirming care or any medications you are taking as part of your gender-affirming journey that we should take into account?
      1. ¿Tiene preocupaciones o consideraciones particulares relacionadas con su atención de afirmación de género o con los medicamentos que está tomando como parte de su proceso de afirmación de género que debamos tener en cuenta?
   3. Do you have any accessibility needs or requirements related to your disability, mobility, or communication that we should be aware of during your healthcare appointments?
      1. ¿Tiene necesidades o requisitos de accesibilidad relacionados con su discapacidad, movilidad o comunicación que debamos conocer durante sus citas médicas?
   4. Are there any specific ways you'd like me to address or refer to your body or health concerns that align with your gender identity and affirm your identity and experiences?
      1. ¿Hay alguna manera específica en la que le gustaría que me refiera o hable sobre su cuerpo o preocupaciones de salud que esté alineada con su identidad de género y afirme su identidad y experiencias?

# **History**

1. Elicit chief complaint and SOCRATES (S: Site - Where is the pain located?, O: Onset - When did the pain start, and was it sudden or gradual?, C: Character - What is the pain like? Is it stabbing, burning, throbbing, etc.?, R: Radiation - Does the pain radiate or spread to any other areas?, A: Associations - Are there any other signs or symptoms associated with the pain?, Time course - Does the pain follow any pattern? How long does it last? Does it come and go?, E: Exacerbating/Relieving factors - What makes the pain better or worse?, S: Severity - How bad is the pain on a scale of 1 to 10, or how does it impact the ability to carry out activities?)
   1. What brings you in today?
      1. ¿Qué le trae a la clínica hoy?
   2. Please tell me more.
      1. Por favor, dígame más.
   3. What symptoms do you have?
      1. ¿Qué síntomas presenta?
   4. When did they start?
      1. ¿Cuándo empezaron?
   5. Do you have discomfort?
      1. ¿Tiene incomodidad?
   6. Does the pain spread anywhere?
      1. ¿Se extiende el dolor a otras partes del cuerpo?
   7. Does anything else feel unusual? Do you have any other symptoms?
      1. ¿Se siente algo más anormal? ¿Tiene algún otro síntoma?
   8. Is it constant, or does it come and go?
      1. ¿Es constante, o va y viene?
   9. What makes the discomfort better? Worse?
      1. ¿Qué lo mejora? ¿Qué lo empeora?
   10. On a scale of 1 to 10, how much does it hurt?
       1. En una escala del uno al diez, ¿cuánto le duele?
2. **General**
   1. How is your diet?
      1. ¿Cómo es su dieta?
   2. Are you fatigued?
      1. ¿Tiene fatiga?
   3. Do you have cold intolerance?
      1. ¿Sufre intolerancia al frío?
   4. Do you have heat intolerance?
      1. ¿Sufre intolerancia al calor?
   5. Please tell me more.
      1. Por favor, dígame más.
3. **Cardiovascular**
   1. Do you have palpitations?
      1. ¿Tiene palpitaciones?
   2. Do you suffer from pain chest?
      1. ¿Sufre del dolor en su pecho?
   3. Do you have difficulty breathing?
      1. ¿Tiene dificultad para respirar?
4. **Musculoesquelético y Piel**
   1. Do you have dry skin?
      1. ¿Tiene la piel seca?
   2. Are you having hair loss?
      1. ¿Se le está cayendo el cabello?
   3. Are your nails brittle?
      1. ¿Sus uñas son frágiles?
   4. Do you have oily skin
      1. ¿Tiene la piel grasa?
   5. Do you sweat excessively?
      1. ¿Suda en exceso?
   6. Do you have muscle weakness?
      1. ¿Tiene debilidad muscular?
   7. Do you have cramps?
      1. ¿Sufre de calambres?
   8. Have you noticed a lump in your neck?
      1. ¿Ha notado un bulto en el cuello?
5. **Ojos**
   1. Do you have swelling around your eyes?
      1. ¿Sufre hinchazón alrededor de sus ojos?
   2. Has your vision changed?
      1. ¿Ha cambiado su vista?
   3. Have you suffered blurry vision?
      1. ¿Ha sufrido visión borrosa?
   4. Are your eyes more sensitive to light?
      1. ¿Sus ojos son más sensibles a la luz?
   5. Do you have eye redness?
      1. ¿Tiene enrojecimiento en sus ojos?
   6. Have you noticed changes in your eyelids?
      1. ¿Ha notado cambios en sus párpados?
6. **Digestivo**
   1. Have you suffered constipation?
      1. ¿Ha sufrido estreñimiento?
   2. Have you had diarrhea?
      1. ¿Ha tenido diarrea?
7. **Psicológico**
   1. Have you had changes in mood?
      1. ¿Ha tenido cambios de humor?
   2. Are you depressed?
      1. ¿Está deprimido/a/e?
   3. Do you have anxiety?
      1. ¿Tiene ansiedad?
   4. Do you have difficulty sleeping?
      1. ¿Tiene dificultad para dormir?
8. **If pertinent to the condition**
   1. Do you have a partner?
      1. ¿Tiene pareja?
   2. Are you active sexually?
      1. ¿Está activo/a/e sexualmente?
   3. Do you have more than one sexual partners?
      1. ¿Tiene más de una pareja sexual?
   4. Does your partner have more sexual partners?
      1. ¿Tu pareja tiene más parejas sexuales?
