# Supplementary material for: Integrating Gender-Affirming Care in a Medical Spanish Endocrine System Curriculum
Source: MedEdPORTAL. 2024 Oct 23;20:11456. doi: 10.15766/mep_2374-8265.11456 (PMC11496385; doi:10.15766/mep_2374-8265.11456)
Supplement: Supplementary file 1 — Facilitator Guide.docxLesson 1 Presentation.pptxLesson 2 Presentation.pptxLesson 3 Presentation.pptxLesson 1 Clinical Endocrine Checklist.docxLesson 2 Clinical Endocrine Checklist.docxLesson 3 Clinical Endocrine Checklist.docxLesson 1 SP Case.docxLesson 2 SP Case.docxLesson 3 SP Case.docxPre-Post Confidence Survey.docxPre-Post Spanish Endocrine Test.docxOSCE SP Diabetic Case.docxOSCE Door Note.docxOSCE Clinical Checklist Diabetic Encounter.docxOSCE Language Rubric for Diabetic Encounter.docx [file mep_2374-8265.11456-s001.zip › K. Pre-Post Confidence Survey.docx]

**Appendix K.** Pre-Post Confidence Survey

Please rate your confidence on a scale of 1 to 5, with 1 being not confident at all and 5 being extremely confident, for the following tasks:

**Assisting a patient with an endocrine problem**

1 – Not confident at all

2 – Slightly confident

3 – Moderately confident

4 – Very confident

5 – Extremely confident

**Describing the functions of the endocrine system in Spanish to patients**

1 – Not confident at all

2 – Slightly confident

3 – Moderately confident

4 – Very confident

5 – Extremely confident

**Using specific Spanish vocabulary and grammar to conduct a comprehensive medical history of a patient with diabetes**

1 – Not confident at all

2 – Slightly confident

3 – Moderately confident

4 – Very confident

5 – Extremely confident

**Integrating inclusive language with patients who have endocrine issues**

1 – Not confident at all

2 – Slightly confident

3 – Moderately confident

4 – Very confident

5 – Extremely confident
