# Supplementary material for: Integrating Gender-Affirming Care in a Medical Spanish Endocrine System Curriculum
Source: MedEdPORTAL. 2024 Oct 23;20:11456. doi: 10.15766/mep_2374-8265.11456 (PMC11496385; doi:10.15766/mep_2374-8265.11456)
Supplement: Supplementary file 1 — Facilitator Guide.docxLesson 1 Presentation.pptxLesson 2 Presentation.pptxLesson 3 Presentation.pptxLesson 1 Clinical Endocrine Checklist.docxLesson 2 Clinical Endocrine Checklist.docxLesson 3 Clinical Endocrine Checklist.docxLesson 1 SP Case.docxLesson 2 SP Case.docxLesson 3 SP Case.docxPre-Post Confidence Survey.docxPre-Post Spanish Endocrine Test.docxOSCE SP Diabetic Case.docxOSCE Door Note.docxOSCE Clinical Checklist Diabetic Encounter.docxOSCE Language Rubric for Diabetic Encounter.docx [file mep_2374-8265.11456-s001.zip › L. Pre-Post Spanish Endocrine Test.docx]

**Appendix L.** Pre–post Spanish endocrine test

1. Las glándulas _____ son un grupo de glándulas en el cuerpo que secretan hormonas.

1. Sudoríparas
2. Salivales
3. Linfáticas
4. **Endócrinas**

2. Las hormonas son secretadas por las células de una glándula endocrina hacia el _______

- 1. Tracto urinario
  2. **Sangre**
  3. Ganglios linfáticos
  4. Tracto gastrointestinal

3. Un _____ es una molécula secretada por una célula que tiene un efecto sobre otra célula a cierta distancia.

1. Proteína
2. ADN
3. ARN
4. **Hormona**

4. ¿Sobre qué órgano se encuentra la glándula suprarrenal?

1. El páncreas
2. El hígado
3. **El riñón**
4. El cerebro

5. Las glándulas neuroendocrinas del páncreas secretan todas las hormonas siguientes, excepto:

1. Insulina
2. Glucagón
3. Somatostatina
4. **Adrenalina**

6. La ansiedad, la fatiga, el agrandamiento de la glándula tiroides, la caída del cabello, el temblor de las manos son síntomas de ____________

1. Hipotiroidismo
2. Diabetes
3. **Hipertiroidismo**
4. Insuficiencia cardiaca

7. La función de la glándula ______ es regular el metabolismo general del cuerpo.

1. Suprarrenal
2. **Tiroides**
3. Paratiroidea
4. Pituitaria

8. ¿Qué está elevado en la sangre de un paciente diabético?

- 1. **Glucosa/azúcar**
  2. Bilirrubina
  3. Dopamina
  4. Eosinófilos

9. ¿Qué hormona lleva la glucosa a las células?

- 1. Dopamina
  2. **Insulina**
  3. Acetilcolina
  4. Testosterona

10. Un bebé grande nace de una mujer con una enfermedad. ¿Qué enfermedad es más probable que tenga la madre?

- 1. Lupus
  2. **Diabetes materna**
  3. Cáncer
  4. Hepatitis

11. ¿Qué es una afección ocular que puede causar ceguera en personas con diabetes?

- 1. Cataratas
  2. **Retinopatía**
  3. Miopia
  4. Scleral Icterus

12. ¿La diabetes tipo 1 ocurre cuando qué órgano no produce insulina?

- 1. **Pancreas**
  2. Estómago
  3. Riñón
  4. Bazo

13. ¿Cuál de los siguientes NO es un síntoma de diabetes?

- 1. Aumento de la sed
  2. Fatiga
  3. La perdida de peso
  4. **Congestión**

14. ¿Qué hormona está asociada con el estrés?

- 1. Estrógeno
  2. Leucocito
  3. Oxitocina
  4. **Cortisol**

15. ¿Qué ataca el sistema inmunológico en la diabetes tipo 1?

- 1. **Células de beta**
  2. Mitochondrion
  3. Células de delta
  4. Todas lo anterior

16. Las hormonas del tiroides son más activas en personas que sufren de…

- 1. **Hipertiroidismo**
  2. Hipotiroidismo
  3. Hipercalcemia
  4. Hipofosfatemia

17. ¿Qué hormona controla el apetito?

- 1. **Ghrelina**
  2. Andrógeno
  3. Progesterona
  4. Inhibina

18. La corteza suprarrenal produce?

- 1. Aldosterona
  2. Cortisol
  3. Dehidroepiandrosterona
  4. **Todo lo anterior**

19. La obesidad es un riesgo para…

- 1. Diabetes
  2. Alto colesterol
  3. Bajo testosterona
  4. **Todo lo anterior**

20. La diabetes tiene riesgo de…

- 1. La gangrena
  2. La infección urinaria
  3. Infección fúngica
  4. **Todo lo anterior**

21. La pubertad acompaña…

- 1. Cambios de vos
  2. Crecimiento del vello púbico
  3. Cambios de apetito
  4. **Todo lo anterior**

22. Síntomas de hipoglucemia incluyen…

- 1. Confusión
  2. Sudor
  3. Taquicardia
  4. **Todo lo anterior**

23. ¿La hormona estimuladora folicular controla la actividad de cuál glándula endocrina?

- 1. Glándula suprarrenal
  2. Páncreas
  3. **Ovario**
  4. Tiroides

24. ¿Dónde se encuentra la glándula pituitaria?

- 1. **La base del cerebro**
  2. Arriba de los riñones
  3. Cerca de los tiroides
  4. La axila

25. ¿Qué hormona se libera cuando estás deshidratado?

- 1. Insulina
  2. Glucagón
  3. Somatostatina
  4. **Hormona antidiurética**

26. El páncreas se encuentra en

- 1. La pierna
  2. **El abdomen**
  3. El pecho
  4. La cabeza

27. ¿Qué hormona funciona para hacerte dormir?

- 1. Estrógeno
  2. Melanina
  3. **Melatonina**
  4. Cortisol

28. ¿Alguien que es bajo y pequeño en estatura podría tener niveles bajos de cuales hormonas?

- 1. **Hormona del crecimiento**
  2. Estrógeno
  3. Glucagón
  4. Hormona estimuladora folicular

29. ¿Qué hormona afecta la producción de glóbulos rojos?

- 1. Melatonina
  2. Adrenalina
  3. Aldosterona
  4. **Eritropoyetina**

30. ¿Qué hormona NO está asociada con el sistema nervioso simpático?

- 1. Adrenalina
  2. **Hormona del crecimiento**
  3. Norepinefrina
  4. Epinefrina

- English below -

1. The _____ glands are a group of glands in the body that secrete hormones.

1. Sudoriparous
2. Salivary
3. Lymphatic
4. **Endocrine**

2. Hormones are secreted by the cells of an endocrine gland into the _______.

1. Urinary tract
2. **Blood**
3. Lymph nodes
4. Gastrointestinal tract

3. A _____ is a molecule secreted by a cell that has an effect on another cell at a certain distance.

1. Protein
2. DNA
3. RNA
4. **Hormone**

4. On what organ is the adrenal gland located?

1. The pancreas
2. The liver
3. **The kidney**
4. The brain

5. The neuroendocrine glands of the pancreas secrete all of the following hormones, except:

1. Insulin
2. Glucagon
3. Somatostatin
4. **Adrenaline**

6. Anxiety, fatigue, enlargement of the thyroid gland, hair loss, and hand tremors are symptoms of _____________.

1. Hypothyroidism
2. Diabetes
3. **Hyperthyroidism**
4. Congestive heart failure

7. The function of the ______ gland is to regulate the body's overall metabolism.

1. Adrenal
2. **Thyroid**
3. Parathyroid
4. Pituitary

8. What is elevated in the blood of a diabetic patient?

1. **Glucose/sugar**
2. Bilirubin
3. Dopamine
4. Eosinophils

9. Which hormone carries glucose into cells?

1. Dopamine
2. **Insulin**
3. Acetylcholine
4. Testosterone

10. A large baby is born to a woman with a disease. What disease is she most likely to have?

1. Lupus
2. **Maternal diabetes**
3. Cancer
4. Hepatitis

11. What is an eye condition that can cause blindness in people with diabetes?

1. Cataracts
2. **Retinopathy**
3. Myopia
4. Scleral Icterus

12. Type 1 diabetes occurs when which organ does not produce insulin?

1. **Pancreas**
2. Stomach
3. Kidney
4. Spleen

13. Which of the following is NOT a symptom of diabetes?

1. Increased thirst
2. Fatigue
3. Weight loss
4. **Congestion**

14. Which hormone is associated with stress?

1. Estrogen
2. Leukocyte
3. Oxytocin
4. **Cortisol**

15. What does the immune system attack in type 1 diabetes?

1. **Beta cells**
2. Mitochondria
3. Delta cells
4. All of the above

16. Thyroid hormones are more active in people who suffer from...

1. **Hyperthyroidism**
2. Hypothyroidism
3. Hypercalcemia
4. Hypophosphatemia

17. Which hormone controls appetite?

1. **Ghrelin**
2. Androgen
3. Progesterone
4. Inhibin

18. The adrenal cortex produces?

1. Aldosterone
2. Cortisol
3. Dehydroepiandrosterone
4. **All of the above**

19. Obesity is a risk factor for...

1. Diabetes
2. High cholesterol
3. Low testosterone
4. **All of the above**

20. Diabetes has a risk of...

1. Gangrene
2. Urinary infection
3. Fungal infection
4. **All of the above**

21. Puberty is accompanied by...

1. Voice changes
2. Pubic hair growth
3. Appetite changes
4. **All of the above**

22. Symptoms of hypoglycemia include...

1. Confusion
2. Sweating
3. Rapid heartbeat
4. **All of the above**

23. The follicle-stimulating hormone controls the activity of which endocrine gland?

1. Adrenal gland
2. Pancreas
3. **Ovary**
4. Thyroid

24. Where is the pituitary gland located?

1. **The base of the brain**
2. Above the kidneys
3. Near the thyroid
4. The armpit

25. Which hormone is released when you are dehydrated?

1. Insulin
2. Glucagon
3. Somatostatin
4. **Antidiuretic hormone**

26. The pancreas is located in the...

1. Leg
2. **Abdomen**
3. Chest
4. Head

27. Which hormone works to make you sleep?

1. Estrogen
2. Melanin
3. **Melatonin**
4. Cortisol

28. Someone who is short in stature could have low levels of which hormones?

1. **Growth hormone**
2. Estrogen
3. Glucagon
4. Follicle-stimulating hormone

29. Which hormone affects the production of red blood cells?

1. Melatonin
2. Adrenaline
3. Aldosterone
4. **Erythropoietin**

30. Which hormone is NOT associated with the sympathetic nervous system?

1. Adrenaline
2. **Growth hormone**
3. Norepinephrine
4. Epinephrine
