# Supplementary material for: Integrating Gender-Affirming Care in a Medical Spanish Endocrine System Curriculum
Source: MedEdPORTAL. 2024 Oct 23;20:11456. doi: 10.15766/mep_2374-8265.11456 (PMC11496385; doi:10.15766/mep_2374-8265.11456)
Supplement: Supplementary file 1 — Facilitator Guide.docxLesson 1 Presentation.pptxLesson 2 Presentation.pptxLesson 3 Presentation.pptxLesson 1 Clinical Endocrine Checklist.docxLesson 2 Clinical Endocrine Checklist.docxLesson 3 Clinical Endocrine Checklist.docxLesson 1 SP Case.docxLesson 2 SP Case.docxLesson 3 SP Case.docxPre-Post Confidence Survey.docxPre-Post Spanish Endocrine Test.docxOSCE SP Diabetic Case.docxOSCE Door Note.docxOSCE Clinical Checklist Diabetic Encounter.docxOSCE Language Rubric for Diabetic Encounter.docx [file mep_2374-8265.11456-s001.zip › A. Facilitator Guide.docx]

**Appendix A.** Facilitator Guide

**Facilitator Guide**

**Lesson plan 1:** Gender-Affirming Medical Spanish Endocrine System I

**Target grammar structure and vocabulary:**

**‘**Alguna vez’, ‘pretérito perfecto compuesto’, and medical terms related to diabetes.

**Duration:** 75 minutes.

**Objectives:**

1. To develop students’ Spanish language proficiency for a clinical history taking, specifically for Hispanic patients with potential diabetes.
2. To gain an understanding of the relevant medical terms and vocabulary associated with diabetes and its complications in Spanish.
3. To recognize and differentiate the symptoms and complications of diabetes in Spanish.
4. To improve students' understanding of the increasing prevalence of type 2 diabetes among Hispanic populations in the United States.
5. To improve students' understanding of the impact of gender-affirming language in clinical practice.
6. To practice using the target grammar structure of "alguna vez" and the ‘pretérito perfecto compuesto’ together in Spanish in a clinical context.

**Materials:**

1. PowerPoint presentation 1.
2. Clinical endocrine checklist 1.

**Before the lesson:**

1. Students are expected to review the target grammar structure of "alguna vez" and the ‘present perfect’ (“pretérito perfecto compuesto”) in Spanish as well as related vocabulary such as terms related to diabetes in Spanish.
2. Students are also required to read the following article: Aguayo-Mazzucato C, Diaque P, Hernandez S, Rosas S, Kostic A, Caballero AE. Understanding the growing epidemic of type 2 diabetes in the Hispanic population living in the United States. Diabetes Metab Res Rev. 2019 Feb;35(2):e3097. doi: 10.1002/dmrr.3097. PMID: 30445663; PMCID: PMC6953173.

**Lesson Plan:**

**Warm-up** (5 minutes):

1. Begin the lesson having students divided in groups of 6.
2. Ask the students sit in a circle and ask the first student to say a sentence in Spanish that uses the present perfect tense. For example: "He visitado a mi médico esta semana" (I have visited my doctor this week). The next student in the circle must then repeat the first student's sentence and add their own sentence using the present perfect tense. For example: "Él me ha recetado un medicamento nuevo" (He has prescribed a new medication for me).
3. Follow up and add more examples from different Spanish-speaking countries.

**Presentation** (45 minutes):

1. Start the lesson by introducing the relevant medical terms and vocabulary associated with diabetes and its complications in Spanish, using visual aids to help students better comprehend the concepts (5 minutes).
2. Have students practice pronouncing and repeating the terms, as well as identifying the Spanish words for different symptoms and complications of diabetes (5 minutes).
3. Provide a brief overview of the history taking process for patients with potential diabetes, including relevant questions and areas of focus and briefly offer a few examples using “alguna vez” (10 minutes).
4. Divide students into small groups and assign each group a role play scenario related to diabetes. Each group will have 5 minutes to prepare and then act out the scenario in Spanish, using the vocabulary and medical terms covered in the lesson (5 minutes).
5. Present some questions related to the endocrine system for patients. Students should discuss the questions in pairs and decide whether they are well formulated in terms of inclusivity. If not, they should offer an alternative. Afterwards, ask some students to share their questions and offer feedback with examples (10 minutes).
6. Students will create an inclusive diabetes awareness poster in Spanish, incorporating key vocabulary and concepts covered in the lesson. Students will present their posters to another group and briefly explain their design choices and the message they are trying to convey (5 minutes).
7. Discuss the assigned reading and the topics mentioned in it, such as the diabetes among Hispanics living in the US and the potential strategies to mitigate its impact. Discuss the importance of validating culture (including food) (5 minutes).

* If necessary, provide students with additional resources such as readings or online exercises to reinforce the objectives covered in the lesson.

**Practice with SPs** (25 minutes):

Students will be divided into groups of three and assigned a standardized patient that presents symptoms of diabetes. Each student will take turns asking questions about the patient's history and any relevant symptoms or complications of diabetes. A peer tutor will be assigned to the table to assist with any linguistic issues, and the instructor will also rotate among the tables.

**Lesson plan 2:** Gender-Affirming Medical Spanish Endocrine System II

**Target Grammar Structure:** Formal Commands.

**Vocabulary:** Medical terms related to the endocrine system, gender-affirming language in Spanish and communicating bad news to patients.

**Duration:** 75 minutes.

**Objectives:**

1. To develop students’ Spanish language proficiency for a clinical history taking, specifically for patients with potential endocrine system issues.
2. To recognize and differentiate some of the symptoms and complications of the endocrine system disorders in Spanish.
3. To learn how to be sensitive when giving bad news to Hispanic patients.
4. To improve students' understanding of the importance of using gender-affirming language when communicating with patients who are transgender or with non-binary gender.
5. To practice using formal commands in Spanish in a clinical context.

**Materials:**

1. PowerPoint presentation 2.
2. Clinical endocrine checklist 2.

**Before the lesson:**

1. Students are expected to review the use of formal commands in Spanish as well as the medical terms related to the endocrine system in Spanish.
2. Students are required to read the following article: "Providing Affirmative Care for Patients with Non-binary Gender Identities." LGBTQIA Health Education. National LGBT Health Education Center, a program of the Fenway Institute.”n.d.. <https://www.lgbtqiahealtheducation.org/wp-content/uploads/2017/02/Providing-Affirmative-Care-for-People-with-Non-Binary-Gender-Identities.pdf>.

**Lesson Plan:**

**Warm-up** (5 minutes):

1. Begin the lesson by dividing the students into pairs and having them taking turns asking and answering questions in Spanish related to endocrine scenarios. For example, one student can ask, "¿Qué síntomas tiene usted?" (What symptoms do you have?) and the other student can answer, "Tengo heridas en los pies y visión borrosa" (I have a skin injuries and blurred vision). Then, the other student can say a formal command, such as “Por favor, siéntese aquí” (Please, sit down here) or “No toque sus heridas” (Do not touch your injuries).
2. To make the activity more challenging, set a time limit for each question and answer exchange, such as 30 seconds, and encourage students to use formal commands and medical terminology in their questions and responses.

**Presentation** (45 minutes):

1. Start the lesson by introducing relevant medical terms and vocabulary associated with the endocrine system in Spanish, using visual aids to help students better comprehend the concepts (5 minutes).
2. Have students practice pronouncing and repeating the terms, as well as identifying the Spanish words for different symptoms and complications of endocrine system disorders (5 minutes).
3. Provide some suggestions for patients with potential endocrine system issues, focus on diet, moods, and history taking and focus on formal commands (10 minutes).
4. Introduce the importance of using gender-affirming language in medical practice, including regarding the endocrine system, particularly when communicating with transgender or non-binary patients. Provide examples of gender-affirming language in Spanish and discuss the benefits of using such language (5 minutes).
5. Present some examples related to giving bad news to Hispanic patients, and discuss strategies for doing so in a culturally sensitive manner as well as in a gender affirming way (5 minutes).
6. Incidentally show some formal commands that invite the patient to feel understood and supported in the context of receiving bad news (5 minutes).
7. Pair up students and assign each group a role play scenario related to the endocrine system. One student will play the role of a physician, while the other will portray a non-binary patient. Students will have 5 minutes to prepare and then act out the scenario to another group in Spanish, using the vocabulary and medical terms covered in the lesson and incorporating gender-affirming language (10 minutes).

* If necessary, provide students with additional resources such as readings or online exercises to reinforce the objectives covered in the lesson, and encourage further exploration of the endocrine system and gender affirming language.

**Practice with SPs** (25 minutes):

Students will be divided into groups of three and assigned a standardized patient that presents symptoms of an endocrine system disorder. Each student will take turns asking questions about the patient's history and any relevant symptoms or complications of endocrine system disorders. A peer tutor will be assigned to the table to assist with any linguistic issues, and the instructor will l also rotate among the tables.

**Lesson plan 3:** Gender-Affirming Medical Spanish Endocrine System I

**Target Grammar Structure:** The subjunctive mood.

**Vocabulary:** Medical terms related to the endocrine system with a focus on hormones, gender-affirming language in Spanish and communicating with angry patients.

**Duration:** 75 minutes.

**Objectives:**

1. To develop students’ Spanish language proficiency for a clinical history taking, specifically for patients with potential endocrine system issues.
2. To learn about hormones related to the endocrine system in Spanish.
3. To learn how to be culturally sensitive when dealing with angry patients.
4. To understand the importance of using gender-affirming language when communicating with Hispanic patients who are transgender or non-binary in the context of endocrine disorders and hormone therapy.
5. To practice using the subjunctive mood in Spanish when giving instructions to patients regarding endocrine disorder management and treatment.

**Materials:**

1. PowerPoint presentation 3.
2. Clinical endocrine checklist 3.

**Before the lesson:**

1. Students are expected to review the use of the subjunctive mood in Spanish as well as the medical terms related to the endocrine system in Spanish.
2. Students are required to read the following article: Abreu R, Gonzalez K, Mosley D, Pulice-Farrow L, Adam A, Duberli F. "They feel empowered to discriminate against las chicas": Latina Transgender Women’s Experiences Navigating the Healthcare System. Int J Transgend Health. 2022;23:178-193. doi: 10.1080/26895269.2020.1767752.

**Lesson Plan:**

**Warm-up** (5 minutes):

1. Begin the lesson by dividing the students into pairs and giving them a medical scenario related to the endocrine system. For example, a scenario could be "un paciente tiene niveles altos de azúcar en la sangre" (A patient has high blood sugar levels). Each pair of students will take turns creating hypothetical statements related to the scenarios using the subjunctive mood in Spanish. For example, one student can say, "Es importante que el paciente siga una dieta balanceada" (It's important that the patient follows a balanced diet) and the other student can respond with a hypothetical statement using the subjunctive mood, such as "Ojalá que el paciente pueda controlar sus niveles de azúcar" (I hope the patient can control their blood sugar levels).

**Presentation** (45 minutes):

1. Start the lesson by introducing relevant medical terms and vocabulary associated with hormones and the endocrine system in Spanish, using visual aids to help students better comprehend the concepts (5 minutes).
2. Have students practice pronouncing and repeating the terms, as well as identifying the Spanish words for different hormones and endocrine disorders (5 minutes).
3. Provide a brief overview of the endocrine system and how hormones regulate various bodily functions, as well as the different glands that produce hormones (5 minutes).
4. Present some scenarios where the subjunctive mood is used in Spanish and provide examples related to the use of hormones in gender-affirming medical care, and discuss the benefits and potential risks of hormone therapy for transgender and non-binary patients (5 minutes).
5. Teach the SPIKES strategies to communicate bad news to patients using the subjunctive mood for the examples and also include gender-affirming examples (10 minutes).
6. Discuss strategies for providing culturally sensitive and gender-affirming care to patients with endocrine disorders, and how to use formal commands to create a supportive and respectful environment (5 minutes).
7. Pair up students and assign each group a role play scenario related to hormone imbalances and endocrine disorders. One student will play the role of a physician, while the other will portray a patient who needs to start taking hormones for a gender transition. Students will have 5 minutes to prepare and then act out the scenario to another group in Spanish, using the vocabulary and medical terms covered in the lesson and incorporating gender-affirming language (10 minutes).

* If necessary, provide students with additional resources such as readings or online exercises to reinforce the objectives covered in the lesson, and encourage further exploration of hormones and the endocrine system, as well as gender-affirming language in medical practice.

**Practice with SPs** (25 minutes):

Students will be grouped into threes and given a standardized patient who exhibits symptoms of an endocrine imbalance disorder. Each student will take turns asking questions about the patient's history and any relevant symptoms or complications of endocrine system disorders. A peer tutor will be assigned to the table to assist with any linguistic issues, and the instructor will l also rotate among the tables.
