# Supplementary material for: Integrating Gender-Affirming Care in a Medical Spanish Endocrine System Curriculum
Source: MedEdPORTAL. 2024 Oct 23;20:11456. doi: 10.15766/mep_2374-8265.11456 (PMC11496385; doi:10.15766/mep_2374-8265.11456)
Supplement: Supplementary file 1 — Facilitator Guide.docxLesson 1 Presentation.pptxLesson 2 Presentation.pptxLesson 3 Presentation.pptxLesson 1 Clinical Endocrine Checklist.docxLesson 2 Clinical Endocrine Checklist.docxLesson 3 Clinical Endocrine Checklist.docxLesson 1 SP Case.docxLesson 2 SP Case.docxLesson 3 SP Case.docxPre-Post Confidence Survey.docxPre-Post Spanish Endocrine Test.docxOSCE SP Diabetic Case.docxOSCE Door Note.docxOSCE Clinical Checklist Diabetic Encounter.docxOSCE Language Rubric for Diabetic Encounter.docx [file mep_2374-8265.11456-s001.zip › I. Lesson 2 SP Case.docx]

**Appendix I.** Lesson 2 - SP Case

**^Nombre del caso:^** ^Paciente No Binario con Síntomas Gastrointestinales^

**^Actividad Educacional Formativa^**

**^Métodos^**

^Los Pacientes Estandarizados (PEs) recibieron el siguiente guion detallado del perfil del paciente, así como información sobre las características personales y el historial médico relevante para familiarizarse con el caso antes de la simulación.^

^La actividad se llevó a cabo en el aula donde ocurren las clases teóricas. El entorno del encuentro fue una clase con diferentes mesas para 4-5 estudiantes. No hay materiales requeridos aparte de la computadora.^

^Cada grupo de aproximadamente 4 estudiantes contará con un paciente estandarizado y un tutor en su mesa. La duración de cada encuentro será de 20 a 25 minutos.^

^Los PEs fueron seleccionados según su capacidad para representar a diversos pacientes, incluidos los pacientes no binarios hispane/latinx de cualquier edad. Los métodos de entrenamiento incluyen sesiones de ensayo para familiarizarse con el perfil del paciente y su historial médico, así como la manera de expresar los síntomas y preocupaciones, implementadas por una educadora de PEs.^

^Los materiales de capacitación incluyen el guion detallado.^

| ESCENARIO: Ambulatorio, hospitalizado, urgencias, hogar, residencia de ancianos, rehabilitación, grupo, etc. | Ambulatorio |
| --- | --- |
| PERFIL DEL PACIENTE: Información sobre el “paciente” que ayuda a seleccionar un SP y ayuda al aprendiz a comprenderlos como persona. El SP conocerá más información sobre el paciente de la que el aprendiz preguntará, pero permite que el SP represente una personalidad de paciente completamente desarrollada. Si ninguno de los elementos a continuación es particular para el caso, escriba “se pueden usar todos”. | |
| Nombre del paciente | Goya / Noah Martín |
| Pronombre | Elle |
| Rango de edad | 50-60 años |
| Religión/ antecedentes espirituales | Cristiane |
| Sexo (masculino, femenino, intersexual, transgénero…) | Dependiendo del PE, hombre o mujer |
| Orientación sexual (por ejemplo, heterosexual, lesbiana, gay, bisexual, pansexual, queer, asexual) | Queer |
| Expresión de género (por ejemplo, hombre, mujer, género no binario) | No binario |
| Raza y/o etnia | Hispane/Latinx |
| Descripción física (por ejemplo, IMC, rango de altura) | 172 cm de altura y 81 kg de peso |
| Limitaciones físicas | N/A |
| Apariencia del paciente (por ejemplo, desaliñado, bata de hospital, informal de negocios) | Informal |
| Simulación + ubicación (por ejemplo, ninguna, moretones, cicatrices, piercings, tatuajes) | Ninguna |
| Afecto (por ejemplo, agradable, cooperativo) | Agradabele y cooperadore |
| Grupo familiar (por ejemplo, quiénes son familiares, con quién viven) | Vive con su espose |
| Educación | Educación universitaria |
| Nivel de alfabetización en salud | Moderado; entiende instrucciones básicas de salud |
| Empleo, si lo hay: presente y pasado, señalando cualquier estrés actual | Actualmente trabaja como auxiliar de veterinaria. Estrés actual debido a la incapacidad de trabajar por sus síntomas |
| Hogar/sin hogar - tipo de vivienda, número de pisos, propiedad o alquilada | Vive en su propia casa |
| Situación financiera - cualquier estrés actual | Sin estrés financiero |
| Estado del seguro (por ejemplo, no asegurado/infrasegurado/asegurado, público/privado, HMO/PPO) | Asegurade, seguro privado, PPO |
| Hábitos (es decir, dieta, ejercicio, cafeína, fumar, alcohol, drogas) | Dieta variada, ejercicio ocasional (sale a correr de vez en cuando), no consume cafeína, no fuma, consume alcohol de vez en cuando, no usa drogas |
| Actividades (es decir, pasatiempos, deportes, clubes, amigos) | Disfruta de comer y pasar tiempo con su espose |
| Día típico - cuál es la rutina diaria habitual | Se levanta temprano, va a trabajar como auxiliar de veterinaria, vuelve a casa, pasa tiempo con su espose, come, y de vez en cuando sale a correr |

| CASE INFORMATION | |
| --- | --- |
| Queja principal: Lo que el paciente dirá cuando sea recibido por el estudiante. La razón principal del paciente para buscar atención médica, a menudo expresada en sus propias palabras | “Todo lo que como lo vomito y su medicina no funcionó” |
| Preocupaciones adicionales: Otras preocupaciones que el paciente tenga hoy (es decir, síntomas, solicitudes, expectativas, etc.) que se incluirán en la agenda establecida | "Pérdida de peso, diarrea, angustia, picor en la piel, enfado” |
| HISTORIA DE LA ENFERMEDAD ACTUAL: Aunque parte de la historia de la enfermedad actual se dará en el relato de los síntomas del paciente, los aprendices ampliarán la historia durante la sección de preguntas directas. A continuación, describa la historia detallada, generalmente sobre la queja principal, que el estudiante debe desarrollar para hacer una evaluación útil del problema: | |
| Inicio (cuándo; gradual o repentino) | Hace treinta días, gradual |
| Contexto (qué estaba pasando o dónde estaba el paciente cuando se notaron los síntomas por primera vez) | No está claro |
| Duración (cuánto tiempo) | Constante desde hace treinta días |
| Relaciones de tiempo (frecuencia, constante o intermitente) | Constante |
| Ubicación | Estómago |
| Radiación | No |
| Calidad | Vómitos y dolor abdominal |
| Cantidad | N/A |
| Empeorado por qué | Nada |
| Aliviado por qué | Nada |
| Asociado con qué | Pérdida de peso, diarrea, angustia, picor en la piel, enfado |
| Historial médico pasado |  |
| Alergias a medicamentos (nombre y reacción) | Ninguna conocida |
| Alergias ambientales (nombre y reacción) | Ninguna conocida |
| Enfermedades | Ninguna en la infancia, ninguna como adulto |
| Medicaciones | Ninguna |
| Vacunas | Al día |
| Cirugias | Ninguna |
| Accidentes/lesiones/traumas | Ninguno significativo |
| Hospitalicación | Ninguna |
| Historial sexual y reproductivo inclusivo | |
| Prácticas sexuales  Parejas sexuales  Protección: uso de prácticas sexuales más seguras  Uso de anticonceptivos si corresponde  Riesgo de violencia por parte de la pareja íntima | Con su espose  1  No usa  No  No |
| Dieta (describa) | De todo |
| Ejercicio (describa) | De vez en cuando sale a correr |
| Enumere cualquier otro historial social importante o información relevante para este caso | Ninguna otra información relevante mencionada |
| Historial Familiar |  |
| Madre, padre, hermanos, abuelos y otros hallazgos significativos | Madre: Hipotiroidismo. Padre: Colesterol alto. Hermana menor: Jaquecas |

- - English -

**^Name of Case:^** ^Genderqueer Patient with Gastrointestinal Symptoms^

^Formative Educational Activity^

**^Methods^**

^The Standardized Patients (SPs) received the following detailed script of the patient profile, as well as information about personal characteristics and relevant medical history to become familiar with the case before the simulation.^

^The activity took place in the classroom where theoretical classes occur. The encounter setting was a class with different tables for 4-5 students. No materials are required apart from the computer.^

^Each group of approximately 4 students will have a standardized patient and a tutor at their table. The duration of each encounter will be 20 to 25 minutes.^

^The SPs were selected based on their ability to represent diverse patients, including non-binary Hispanic/Latinx patients of any age. The training methods include rehearsal sessions to become familiar with the patient profile and medical history, as well as how to express symptoms and concerns, implemented by an SP educator.^

^The training materials include the detailed script.^

| SETTING: outpatient, in patient, ED, home, nursing home, rehab, group, etc. | Outpatient |
| --- | --- |
| PATIENT PROFILE: Information about the “patient” that helps select an SP and helps the learner get an understanding of them as a person. SP will know more information about the patient than learner will ever ask but allows SP to portray a fully developed patient personality. If none of the items below are particulars for the case, please write “all may be used.” | |
| Patient’s name | Goya / Noah Martín |
| Pronouns | They/Theirs/Them |
| Age range | 50-60 years old |
| Religious/spiritual background | Christian |
| Sex (e.g., male, female, intersex, transwoman, transman) | Depending on the SP. man or woman |
| Sexual orientation (e.g., heterosexual, lesbian, gay, bisexual, pansexual, queer, asexual) | Queer |
| Gender expression (e.g., man, woman, genderqueer) | Genderqueer |
| Race and/or ethnicity | Hispanic/Latinx |
| Physical description (e.g., BMI, height range) | 175 cm in height and 84 kg in weight |
| Physical limitations | N/A |
| Patient appearance (e.g., disheveled, hospital gown, business casual, casual) | Informal |
| Moulage + location (e.g., none, bruises, scars, body piercing, tattoos) | None |
| Affect (e.g., pleasant, cooperative) | Pleasant and cooperative |
| Family group (e.g., who is family, who they live with) | Lives with their spouse |
| Education | University education |
| Level of health literacy | Moderate; understands basic health instructions |
| Employment, if any - present and past, noting any current stresses | Currently works as a veterinary assistant. Current stress due to inability to work because of symptoms |
| Home/homeless - type of dwelling, number of stories, owned or rented | Lives in their own house |
| Financial situation - any current stresses | No financial stress |
| Insurance status (e.g., un/under/insured, public/private, HMO/PPO) | Insured, private insurance, PPO |
| Habits (i.e., diet, exercise, caffeine, smoking, alcohol, drugs) | Varied diet, occasional exercise (goes running occasionally), no caffeine, non-smoker, occasional alcohol (drinks alcohol occasionally), no drug use |
| Activities (i.e., hobbies, sports, clubs, friends) | Enjoys eating and spending time with their spouse |
| Typical day - what is the usual daily routine | Gets up early, goes to work as a veterinary assistant, comes home, spends time with their spouse, eats, and occasionally goes for a run |

| CASE INFORMATION | |
| --- | --- |
| Chief Concern: What the patient will say when greeted by the student. The patient’s primary reason for seeking medical care often stated in their own words. | “Everything I eat, I vomit, and the medicine didn't work” |
| Additional Concerns: Other, if any, concerns the patient has today (i.e., symptoms, requests, expectations, etc.) that will become part of set agenda. | "Weight loss, diarrhea, anxiety, skin itching, anger" |
| HISTORY OF PRESENT ILLNESS: Although some of the HPI will be given in the patient’s symptom story, the learners will expand the story during the direct question section. Below, describe the detailed history, usually about the chief concern, which the student must develop in order to make a useful assessment of the problem: | |
| Onset (when; gradual or sudden) | Thirty days ago, gradual |
| Setting (what was going on or where was patient when symptoms first noticed?) | Not clear |
| Duration (how long) | Constant since thirty days ago |
| Time relationships (frequency, constant or intermittent) | Constant |
| Location | Stomach |
| Radiation | No |
| Quality | Vomiting and abdominal pain |
| Amount | N/A |
| Aggravated by what | Nothing |
| Relieved by what | Nothing |
| Associated with what | Weight loss, diarrhea, anxiety, skin itching, anger |
| Past medical history |  |
| Medication allergies (name and reaction) | None known |
| Environmental allergies (name and reaction) | None known |
| Illnesses | None in childhood, none as an adult |
| Medications | None |
| Vaccinations | Up to date |
| Surgeries | None |
| Accidents/injuries/trauma | None significant |
| Hospitalization | None recent |
| Inclusive sexual and reproductive history | |
| Sexual practices  Sexual partners  Protection: Use of safer sex practices  Use of birth control if appropriate  Risk of intimate partner violence | With their spouse  1 sexual partner  Does not use protection  No  No |
| Diet (describe) | Everything |
| Exercise (describe) | Occasionally goes for a run |
| List any other important social history or information important to this case | None mentioned |
| Family history |  |
| Mother, father, siblings, grandparents, and other significant findings | Mother: Hypothyroidism. Father: High cholesterol. Younger sister: Migraines |
