# Supplementary material for: Integrating Gender-Affirming Care in a Medical Spanish Endocrine System Curriculum
Source: MedEdPORTAL. 2024 Oct 23;20:11456. doi: 10.15766/mep_2374-8265.11456 (PMC11496385; doi:10.15766/mep_2374-8265.11456)
Supplement: Supplementary file 1 — Facilitator Guide.docxLesson 1 Presentation.pptxLesson 2 Presentation.pptxLesson 3 Presentation.pptxLesson 1 Clinical Endocrine Checklist.docxLesson 2 Clinical Endocrine Checklist.docxLesson 3 Clinical Endocrine Checklist.docxLesson 1 SP Case.docxLesson 2 SP Case.docxLesson 3 SP Case.docxPre-Post Confidence Survey.docxPre-Post Spanish Endocrine Test.docxOSCE SP Diabetic Case.docxOSCE Door Note.docxOSCE Clinical Checklist Diabetic Encounter.docxOSCE Language Rubric for Diabetic Encounter.docx [file mep_2374-8265.11456-s001.zip › J. Lesson 3 SP Case.docx]

**Appendix J.** Lesson 3 - SP Case

**^Nombre del caso:^** ^Paciente Transgénero con Bulto en el Pecho^

**^Actividad Educacional Formativa^**

**^Métodos^**

^Los Pacientes Estandarizados (PEs) recibieron el siguiente guion detallado del perfil del paciente, así como información sobre las características personales y el historial médico relevante para familiarizarse con el caso antes de la simulación.^

^La actividad se llevó a cabo en el aula donde ocurren las clases teóricas. El entorno del encuentro fue una clase con diferentes mesas para 4-5 estudiantes. No hay materiales requeridos aparte de la computadora.^

^Cada grupo de aproximadamente 4 estudiantes contará con un paciente estandarizado y un tutor en su mesa. La duración de cada encuentro será de 20 a 25 minutos.^

^Los PEs fueron seleccionados según su capacidad para representar a diversos pacientes, incluidos los pacientes no binarios hispane/latinx de cualquier edad. Los métodos de entrenamiento incluyen sesiones de ensayo para familiarizarse con el perfil del paciente y su historial médico, así como la manera de expresar los síntomas y preocupaciones, implementadas por una educadora de PEs.^

^Los materiales de capacitación incluyen el guion detallado.^

| ESCENARIO: Ambulatorio, hospitalizado, urgencias, hogar, residencia de ancianos, rehabilitación, grupo, etc. | Ambulatorio |
| --- | --- |
| PERFIL DEL PACIENTE: Información sobre el “paciente” que ayuda a seleccionar un SP y ayuda al aprendiz a comprenderlos como persona. El SP conocerá más información sobre el paciente de la que el aprendiz preguntará, pero permite que el SP represente una personalidad de paciente completamente desarrollada. Si ninguno de los elementos a continuación es particular para el caso, escriba “se pueden usar todos”. | |
| Nombre del paciente | Raúl/Laura López |
| Pronombre | Elle |
| Rango de edad | 30-40 años |
| Religión/ antecedentes espirituales | Cristiane |
| Sexo (masculino, femenino, intersexual, transgénero…) | Mujer transgénero |
| Orientación sexual (por ejemplo, heterosexual, lesbiana, gay, bisexual, pansexual, queer, asexual) | Pansexual |
| Expresión de género (por ejemplo, hombre, mujer, género no binario) | No binario |
| Raza y/o etnia | Latinx |
| Descripción física (por ejemplo, IMC, rango de altura) | 165 cm de altura y 96 kg de peso, IMC elevado |
| Limitaciones físicas | Ninguna |
| Apariencia del paciente (por ejemplo, desaliñado, bata de hospital, informal de negocios) | Informal de negocios |
| Simulación + ubicación (por ejemplo, ninguna, moretones, cicatrices, piercings, tatuajes) | Ninguna visible |
| Afecto (por ejemplo, agradable, cooperativo) | Agradabele y cooperadore |
| Grupo familiar (por ejemplo, quiénes son familiares, con quién viven) | Vive sole, soltere |
| Educación | Educación universitaria |
| Nivel de alfabetización en salud | Moderado; entiende instrucciones básicas de salud |
| Empleo, si lo hay: presente y pasado, señalando cualquier estrés actual | Secretarie, sin estrés actual significativo |
| Hogar/sin hogar - tipo de vivienda, número de pisos, propiedad o alquilada | Vive en un apartamento alquilado |
| Situación financiera - cualquier estrés actual | Estable, sin estrés financiero |
| Estado del seguro (por ejemplo, no asegurado/infrasegurado/asegurado, público/privado, HMO/PPO) | Asegurade, PPO |
| Hábitos (es decir, dieta, ejercicio, cafeína, fumar, alcohol, drogas) | Variada, come de todo. Sale a correr de vez en cuando. Fuma, pero no consume alcohol. |
| Actividades (es decir, pasatiempos, deportes, clubes, amigos) | Comer, salir a correr |
| Día típico - cuál es la rutina diaria habitual | Rutina diaria regular, trabajo de oficina y tiempo libre dedicado a comer y correr ocasionalmente correr |

| CASE INFORMATION | |
| --- | --- |
| Queja principal: Lo que el paciente dirá cuando sea recibido por el estudiante. La razón principal del paciente para buscar atención médica, a menudo expresada en sus propias palabras | "He ganado mucho peso recientemente y me noto un bulto en el pecho" |
| Preocupaciones adicionales: Otras preocupaciones que el paciente tenga hoy (es decir, síntomas, solicitudes, expectativas, etc.) que se incluirán en la agenda establecida | “Sed y cansancio general” |
| HISTORIA DE LA ENFERMEDAD ACTUAL: Aunque parte de la historia de la enfermedad actual se dará en el relato de los síntomas del paciente, los aprendices ampliarán la historia durante la sección de preguntas directas. A continuación, describa la historia detallada, generalmente sobre la queja principal, que el estudiante debe desarrollar para hacer una evaluación útil del problema: | |
| Inicio (cuándo; gradual o repentino) | Hace cuatro semanas, de manera gradual |
| Contexto (qué estaba pasando o dónde estaba el paciente cuando se notaron los síntomas por primera vez) | Los síntomas se notaron mientras Raúl/Laura estaba en su rutina diaria, sin ningún evento desencadenante específico |
| Duración (cuánto tiempo) | Cuatro semanas |
| Relaciones de tiempo (frecuencia, constante o intermitente) | Los síntomas son constantes |
| Ubicación | Todo el cuerpo, con un bulto localizado en el pecho |
| Radiación | No hay radiación del dolor |
| Calidad | Sensación de aumento de peso, sed y cansancio |
| Cantidad | El bulto en el pecho y el aumento de peso son notables; la sed y el cansancio son constantes |
| Empeorado por qué | Nada empeora los síntomas |
| Aliviado por qué | Nada alivia los síntomas |
| Asociado con qué | Aumento de peso, sed, cansancio y bulto en el pecho |
| Historial médico pasado |  |
| Alergias a medicamentos (nombre y reacción) | Ninguna |
| Alergias ambientales (nombre y reacción) | Ninguna |
| Enfermedades | Ninguna enfermedad crónica |
| Medicaciones | Terapia hormonal para la transición de género |
| Vacunas | Al día |
| Cirugias | Cirugía de reasignación de género |
| Accidentes/lesiones/traumas | Ninguno |
| Hospitalicación | No ha sido hospitalizade |
| Historial sexual y reproductivo inclusivo | |
| Prácticas sexuales  Parejas sexuales  Protección: uso de prácticas sexuales más seguras  Uso de anticonceptivos si corresponde  Riesgo de violencia por parte de la pareja íntima | Prácticas sexuales seguras  Varias  Preservativo  N/A  No |
| Dieta (describa) | Variedad en la dieta, come de todo |
| Ejercicio (describa) | Sale a correr de vez en cuando |
| Enumere cualquier otro historial social importante o información relevante para este caso | Ninguno adicional |
| Historial Familiar |  |
| Madre, padre, hermanos, abuelos y otros hallazgos significativos | Madre: Saludable. Padre: Saludable. No hermanos. |

- - English -

**^Name of Case:^** ^Transgender Patient with Lump in their breast^

^Formative Educational Activity^

**^Methods^**

^The Standardized Patients (SPs) received the following detailed script of the patient profile, as well as information about personal characteristics and relevant medical history to become familiar with the case before the simulation.^

^The activity took place in the classroom where theoretical classes occur. The encounter setting was a class with different tables for 4-5 students. No materials are required apart from the computer.^

^Each group of approximately 4 students will have a standardized patient and a tutor at their table. The duration of each encounter will be 20 to 25 minutes.^

^The SPs were selected based on their ability to represent diverse patients, including non-binary Hispanic/Latinx patients of any age. The training methods include rehearsal sessions to become familiar with the patient profile and medical history, as well as how to express symptoms and concerns, implemented by an SP educator.^

^The training materials include the detailed script.^

| SETTING: outpatient, in patient, ED, home, nursing home, rehab, group, etc. | Outpatient |
| --- | --- |
| PATIENT PROFILE: Information about the “patient” that helps select an SP and helps the learner get an understanding of them as a person. SP will know more information about the patient than learner will ever ask but allows SP to portray a fully developed patient personality. If none of the items below are particulars for the case, please write “all may be used.” | |
| Patient’s name | Raúl/Laura López |
| Pronouns | They/Theirs/Them |
| Age range | 30-40 years old |
| Religious/spiritual background | Christian |
| Sex (e.g., male, female, intersex, transwoman, transman) | Transgender woman |
| Sexual orientation (e.g., heterosexual, lesbian, gay, bisexual, pansexual, queer, asexual) | Pansexual |
| Gender expression (e.g., man, woman, genderqueer) | Genderqueer |
| Race and/or ethnicity | Latinx |
| Physical description (e.g., BMI, height range) | 165 cm in height and 96 kg in weight, elevated BMI |
| Physical limitations | None |
| Patient appearance (e.g., disheveled, hospital gown, business casual, casual) | Business casual |
| Moulage + location (e.g., none, bruises, scars, body piercing, tattoos) | None visible |
| Affect (e.g., pleasant, cooperative) | Pleasant and cooperative |
| Family group (e.g., who is family, who they live with) | Lives alone, single |
| Education | University graduate |
| Level of health literacy | Moderate; understands basic health instructions |
| Employment, if any - present and past, noting any current stresses | Secretary, no significant current stress |
| Home/homeless - type of dwelling, number of stories, owned or rented | Lives in a rented one-floor apartment |
| Financial situation - any current stresses | Stable, no current financial stress |
| Insurance status (e.g., un/under/insured, public/private, HMO/PPO) | Insured, PPO |
| Habits (i.e., diet, exercise, caffeine, smoking, alcohol, drugs) | Varied, they eat everything. Goes running occasionally. They smoke, but do not drink alcohol. |
| Activities (i.e., hobbies, sports, clubs, friends) | Eating, running |
| Typical day - what is the usual daily routine | Regular daily routine, office work, and free time spent eating and occasionally running |

| CASE INFORMATION | |
| --- | --- |
| Chief Concern: What the patient will say when greeted by the student. The patient’s primary reason for seeking medical care often stated in their own words. | "I have gained a lot of weight recently and I noticed a lump in my chest” |
| Additional Concerns: Other, if any, concerns the patient has today (i.e., symptoms, requests, expectations, etc.) that will become part of set agenda. | "Thirst and general fatigue" |
| HISTORY OF PRESENT ILLNESS: Although some of the HPI will be given in the patient’s symptom story, the learners will expand the story during the direct question section. Below, describe the detailed history, usually about the chief concern, which the student must develop in order to make a useful assessment of the problem: | |
| Onset (when; gradual or sudden) | Four weeks ago, gradual |
| Setting (what was going on or where was patient when symptoms first noticed?) | The symptoms were noticed while Raúl/Laura was going about their daily routine, without any specific triggering event |
| Duration (how long) | Four weeks |
| Time relationships (frequency, constant or intermittent) | The symptoms are constant |
| Location | All over the body, with a lump localized in the chest |
| Radiation | No radiation of the pain |
| Quality | Feeling of weight gain, thirst, and fatigue |
| Amount | The lump in the chest and the weight gain are noticeable; the thirst and fatigue are constant |
| Aggravated by what | Nothing worsens the symptoms |
| Relieved by what | Nothing relieves the symptoms |
| Associated with what | Weight gain, thirst, fatigue, and lump in the chest |
| Past medical history |  |
| Medication allergies (name and reaction) | None |
| Environmental allergies (name and reaction) | None |
| Illnesses | No chronic diseases |
| Medications | None |
| Vaccinations | Up to date |
| Surgeries | Hormone therapy for gender transition |
| Accidents/injuries/trauma | None |
| Hospitalization | They have not been hospitalized |
| Inclusive sexual and reproductive history | |
| Sexual practices  Sexual partners  Protection: Use of safer sex practices  Use of birth control if appropriate  Risk of intimate partner violence | Safe sexual practices  Several sexual partners  Condom  N/A  No |
| Diet (describe) | Varied diet, eats everything |
| Exercise (describe) | Goes running occasionally |
| List any other important social history or information important to this case | None mentioned |
| Family history |  |
| Mother, father, siblings, grandparents, and other significant findings | Mother: healthy. Father: healthy. No siblings |
