# Supplementary material for: Integrating Gender-Affirming Care in a Medical Spanish Endocrine System Curriculum
Source: MedEdPORTAL. 2024 Oct 23;20:11456. doi: 10.15766/mep_2374-8265.11456 (PMC11496385; doi:10.15766/mep_2374-8265.11456)
Supplement: Supplementary file 1 — Facilitator Guide.docxLesson 1 Presentation.pptxLesson 2 Presentation.pptxLesson 3 Presentation.pptxLesson 1 Clinical Endocrine Checklist.docxLesson 2 Clinical Endocrine Checklist.docxLesson 3 Clinical Endocrine Checklist.docxLesson 1 SP Case.docxLesson 2 SP Case.docxLesson 3 SP Case.docxPre-Post Confidence Survey.docxPre-Post Spanish Endocrine Test.docxOSCE SP Diabetic Case.docxOSCE Door Note.docxOSCE Clinical Checklist Diabetic Encounter.docxOSCE Language Rubric for Diabetic Encounter.docx [file mep_2374-8265.11456-s001.zip › M. OSCE SP Diabetic Case.docx]

**Appendix M.** OSCE - SP Diabetic Case

**Nombre del caso:** Caso de Paciente Estandarizado Diabético

**Actividad Educacional Formativa**

**Métodos**

Los Pacientes Estandarizados (PEs) recibieron el siguiente guion detallado del perfil del paciente, así como información sobre las características personales y el historial médico relevante para familiarizarse con el caso antes de la simulación.

La actividad se llevó a cabo en el área de habilidades clínicas. El entorno del encuentro fue una habitación clínica que simula una sala médica convencional con una silla reclinable para el paciente y un taburete para el personal sanitario. No hay materiales requeridos aparte de una libreta y un bolígrafo.

Cada estudiante contará con un paciente estandarizado y un evaluador en la sala. La duración de cada encuentro será de 15 minutos.

Los PEs fueron seleccionados según su capacidad para representar a diversos pacientes, incluidos los pacientes no binarios hispane/latinx de cualquier edad. Los métodos de entrenamiento incluyen sesiones de ensayo para familiarizarse con el perfil del paciente y su historial médico, así como la manera de expresar los síntomas y preocupaciones, implementadas por una educadora de PEs.

Los materiales de capacitación incluyen el guion detallado.

| ESCENARIO: Ambulatorio, hospitalizado, urgencias, hogar, residencia de ancianos, rehabilitación, grupo, etc. | Ambulatorio |
| --- | --- |
| PERFIL DEL PACIENTE: Información sobre el “paciente” que ayuda a seleccionar un SP y ayuda al aprendiz a comprenderlos como persona. El SP conocerá más información sobre el paciente de la que el aprendiz preguntará, pero permite que el SP represente una personalidad de paciente completamente desarrollada. Si ninguno de los elementos a continuación es particular para el caso, escriba “se pueden usar todos”. | |
| Nombre del paciente | Juan/ Juana Hernández |
| Pronombre | Elle |
| Rango de edad | 62 años |
| Religión/ antecedentes espirituales | Cristiane |
| Sexo (masculino, femenino, intersexual, transgénero…) | Dependiendo del PE, hombre o mujer |
| Orientación sexual (por ejemplo, heterosexual, lesbiana, gay, bisexual, pansexual, queer, asexual) | Queer |
| Expresión de género (por ejemplo, hombre, mujer, género no binario) | No binario |
| Raza y/o etnia | Hispane/Latinx |
| Descripción física (por ejemplo, IMC, rango de altura) | 178 cm de altura y 94 kg de peso |
| Limitaciones físicas | N/A |
| Apariencia del paciente (por ejemplo, desaliñado, bata de hospital, informal de negocios) | Informal |
| Simulación + ubicación (por ejemplo, ninguna, moretones, cicatrices, piercings, tatuajes) | Grandes heridas en los pies y entumecimiento en pies y manos |
| Afecto (por ejemplo, agradable, cooperativo) | Agradabele y cooperadore |
| Grupo familiar (por ejemplo, quiénes son familiares, con quién viven) | Vive con su pareja y su hije adulte |
| Educación | Educación secundaria completa |
| Nivel de alfabetización en salud | Moderado; entiende instrucciones básicas de salud |
| Empleo, si lo hay: presente y pasado, señalando cualquier estrés actual | Actualmente trabaja de agente inmobiliario |
| Hogar/sin hogar - tipo de vivienda, número de pisos, propiedad o alquilada | Vive en una casa alquilada |
| Situación financiera - cualquier estrés actual | No presenta estrés |
| Estado del seguro (por ejemplo, no asegurado/infrasegurado/asegurado, público/privado, HMO/PPO) | Asegurade, seguro privado, PPO |
| Hábitos (es decir, dieta, ejercicio, cafeína, fumar, alcohol, drogas) | Dieta alta en carbohidratos, hace deporte en el gimnasio tres veces por semana, no consume cafeína, no fuma, consume alcohol ocasionalmente (vino con la cena), no usa drogas |
| Actividades (es decir, pasatiempos, deportes, clubes, amigos) | Disfruta de jugar en el casino |
| Día típico - cuál es la rutina diaria habitual | Se levanta temprano, trabaja hasta las cinco de la tarde, pasa tiempo con su familia por la noche |

| CASE INFORMATION | |
| --- | --- |
| Queja principal: Lo que el paciente dirá cuando sea recibido por el estudiante. La razón principal del paciente para buscar atención médica, a menudo expresada en sus propias palabras | "Tengo grandes heridas en los pies, diarrea y malestar general” |
| Preocupaciones adicionales: Otras preocupaciones que el paciente tenga hoy (es decir, síntomas, solicitudes, expectativas, etc.) que se incluirán en la agenda establecida | "También tengo entumecimiento en las manos y los pies” |
| HISTORIA DE LA ENFERMEDAD ACTUAL: Aunque parte de la historia de la enfermedad actual se dará en el relato de los síntomas del paciente, los aprendices ampliarán la historia durante la sección de preguntas directas. A continuación, describa la historia detallada, generalmente sobre la queja principal, que el estudiante debe desarrollar para hacer una evaluación útil del problema: | |
| Inicio (cuándo; gradual o repentino) | Las heridas comenzaron hace una o dos semanas. Inicialmente eran pequeñas, pero han crecido y ahora son muy dolorosas |
| Contexto (qué estaba pasando o dónde estaba el paciente cuando se notaron los síntomas por primera vez) | “Me desperté un día con ellas, pero han ido creciendo” |
| Duración (cuánto tiempo) | Constante desde entonces |
| Relaciones de tiempo (frecuencia, constante o intermitente) | Constante, sin alivio |
| Ubicación | Pies y manos |
| Radiación | N/A |
| Calidad | Dolorosa y enrojecida |
| Cantidad | Grandes heridas abiertas |
| Empeorado por qué | Caminar y estar de pie |
| Aliviado por qué | No ha encontrado alivio efectivo |
| Asociado con qué | Entumecimiento en manos y pies |
| Historial médico pasado |  |
| Alergias a medicamentos (nombre y reacción) | Ninguna conocida |
| Alergias ambientales (nombre y reacción) | Ninguna conocida |
| Enfermedades | Diabetes Mellitus Tipo 2 |
| Medicaciones | Metformina |
| Vacunas | Al día |
| Cirugias | Apendicectomía a los 25 años |
| Accidentes/lesiones/traumas | Ninguno significativo |
| Hospitalización | Ninguna reciente |
| Historial sexual y reproductivo inclusivo | |
| Prácticas sexuales  Parejas sexuales  Protección: uso de prácticas sexuales más seguras  Uso de anticonceptivos si corresponde  Riesgo de violencia por parte de la pareja íntima | Sin sexo  0  N/A  No  No |
| Dieta (describa) | Dieta balanceada pero alta en carbohidratos |
| Ejercicio (describa) | Gimnasio tres veces a la semana |
| Enumere cualquier otro historial social importante o información relevante para este caso | Ninguna otra información relevante mencionada |
| Historial Familiar |  |
| Madre, padre, hermanos, abuelos y otros hallazgos significativos | Madre: Diabetes Mellitus Tipo 2. Padre: Hipertensión. Hermanos: Saludables |

- *- English -*

**Case Name:** Caso de Paciente Estandarizado Diabético

**Formative Educational Activity**

**Methods**

The Standardized Patients (SPs) received the following detailed script of the patient profile, as well as information about personal characteristics and relevant medical history to familiarize themselves with the case before the simulation.

The activity was conducted in the clinical skills area. The encounter environment was a clinical room simulating a conventional medical room with a recliner chair for the patient and a stool for the healthcare staff. No materials are required apart from a notebook and a pen.

Each student will have a standardized patient and an evaluator in the room. The duration of each encounter will be 15 minutes.

The SPs were selected based on their ability to represent diverse patients, including non-binary Hispanic/Latinx patients of any age. The training methods include rehearsal sessions to familiarize themselves with the patient profile and medical history, as well as how to express symptoms and concerns, implemented by an SP educator.

The training materials include the detailed script.

| SETTING: outpatient, in patient, ED, home, nursing home, rehab, group, etc. | Outpatient |
| --- | --- |
| PATIENT PROFILE: Information about the “patient” that helps select an SP and helps the learner get an understanding of them as a person. SP will know more information about the patient than learner will ever ask but allows SP to portray a fully developed patient personality. If none of the items below are particulars for the case, please write “all may be used.” | |
| Patient’s name | Juan/ Juana Hernández |
| Pronouns | They/Theirs/Them |
| Age range | 62 years old |
| Religious/spiritual background | Christian |
| Sex (e.g., male, female, intersex, transwoman, transman) | Depending on the SP. man or woman |
| Sexual orientation (e.g., heterosexual, lesbian, gay, bisexual, pansexual, queer, asexual) | Queer |
| Gender expression (e.g., man, woman, genderqueer) | Genderqueer |
| Race and/or ethnicity | Hispanic/Latinx |
| Physical description (e.g., BMI, height range) | 178 cm in height and 94 kg in weight |
| Physical limitations | N/A |
| Patient appearance (e.g., disheveled, hospital gown, business casual, casual) | Informal |
| Moulage + location (e.g., none, bruises, scars, body piercing, tattoos) | Big injuries in feet and numbness in feet and hands |
| Affect (e.g., pleasant, cooperative) | Pleasant and cooperative |
| Family group (e.g., who is family, who they live with) | Lives with their spouse and adult child |
| Education | Highschool education |
| Level of health literacy | Moderate; understands basic health instructions |
| Employment, if any - present and past, noting any current stresses | Currently works as a realtor |
| Home/homeless - type of dwelling, number of stories, owned or rented | Rents a house |
| Financial situation - any current stresses | No stress |
| Insurance status (e.g., un/under/insured, public/private, HMO/PPO) | Insured, private insurance, PPO |
| Habits (i.e., diet, exercise, caffeine, smoking, alcohol, drugs) | Diet high in carbs, goes to the gym three times a week, no caffeine, non-smoker, occasional alcohol (wine during dinners), no drug use |
| Activities (i.e., hobbies, sports, clubs, friends) | Enjoys the casino |
| Typical day - what is the usual daily routine | Gets up early, works until 5 pm, spends time with their family at night |

| CASE INFORMATION | |
| --- | --- |
| Chief Concern: What the patient will say when greeted by the student. The patient’s primary reason for seeking medical care often stated in their own words. | "I have large wounds on my feet, diarrhea, and general discomfort" |
| Additional Concerns: Other, if any, concerns the patient has today (i.e., symptoms, requests, expectations, etc.) that will become part of set agenda. | "I also have numbness in my hands and feet” |
| HISTORY OF PRESENT ILLNESS: Although some of the HPI will be given in the patient’s symptom story, the learners will expand the story during the direct question section. Below, describe the detailed history, usually about the chief concern, which the student must develop in order to make a useful assessment of the problem: | |
| Onset (when; gradual or sudden) | The wounds started a week or two weeks ago. Initially, they were small, but they have grown and are now very painful |
| Setting (what was going on or where was patient when symptoms first noticed?) | “I woke up with them, but they have grown” |
| Duration (how long) | Constant since then |
| Time relationships (frequency, constant or intermittent) | Constant, no relief |
| Location | Feet and hands |
| Radiation | N/A |
| Quality | Painful and reddened |
| Amount | Large open wounds |
| Aggravated by what | Walking and standing |
| Relieved by what | Hasn't found effective relief |
| Associated with what | Numbness in hands and feet |
| Past medical history |  |
| Medication allergies (name and reaction) | None known |
| Environmental allergies (name and reaction) | None known |
| Illnesses | Type 2 Diabetes Mellitus |
| Medications | Metformin |
| Vaccinations | Up to date |
| Surgeries | Appendectomy at age 25 |
| Accidents/injuries/trauma | None significant |
| Hospitalization | None recent |
| Inclusive sexual and reproductive history | |
| Sexual practices  Sexual partners  Protection: Use of safer sex practices  Use of birth control if appropriate  Risk of intimate partner violence | Inactive  0  N/A  No  No |
| Diet (describe) | Balanced diet but high in carbohydrates |
| Exercise (describe) | Gym three times a week |
| List any other important social history or information important to this case | No other relevant information mentioned |
| Family history |  |
| Mother, father, siblings, grandparents, and other significant findings | Mother: Type 2 Diabetes Mellitus. Father: Hypertension. Siblings: Healthy |
