# Supplementary material for: Integrating Gender-Affirming Care in a Medical Spanish Endocrine System Curriculum
Source: MedEdPORTAL. 2024 Oct 23;20:11456. doi: 10.15766/mep_2374-8265.11456 (PMC11496385; doi:10.15766/mep_2374-8265.11456)
Supplement: Supplementary file 1 — Facilitator Guide.docxLesson 1 Presentation.pptxLesson 2 Presentation.pptxLesson 3 Presentation.pptxLesson 1 Clinical Endocrine Checklist.docxLesson 2 Clinical Endocrine Checklist.docxLesson 3 Clinical Endocrine Checklist.docxLesson 1 SP Case.docxLesson 2 SP Case.docxLesson 3 SP Case.docxPre-Post Confidence Survey.docxPre-Post Spanish Endocrine Test.docxOSCE SP Diabetic Case.docxOSCE Door Note.docxOSCE Clinical Checklist Diabetic Encounter.docxOSCE Language Rubric for Diabetic Encounter.docx [file mep_2374-8265.11456-s001.zip › N. OSCE Door Note.docx]

# Appendix N. Door Note for Endocrine Medical Spanish OSCE

# Door note used for the Medical Spanish Objective Structured Clinical Examination (OSCE) station focused on endocrine-related case.

You have 25 minutes for this encounter, *including reading the door note.*

**Patient Name:** Juana/Juan Hernández

**Date of Birth:** April 15, 1962

**Chief Complaint:** Large wounds on feet, diarrhea, general discomfort, numbness in hands and feet.

**Medical History:** Type 2 diabetes, managed with medication and dietary changes.

**Presenting Symptoms:**

**Large Wounds on Feet:** Painful wounds on soles, worsening over weeks.

**Diarrhea:** Frequent watery episodes with abdominal discomfort.

**General** Discomfort: Malaise without focal symptoms.

**Numbness in Hands and Feet:** Progressive tingling and numbness.

**Additional Information:**

**Pronouns:** Preferred use of neutral pronoun "elle".

**Gender Identity:** Non-binary
