# Supplementary material for: Integrating Gender-Affirming Care in a Medical Spanish Endocrine System Curriculum
Source: MedEdPORTAL. 2024 Oct 23;20:11456. doi: 10.15766/mep_2374-8265.11456 (PMC11496385; doi:10.15766/mep_2374-8265.11456)
Supplement: Supplementary file 1 — Facilitator Guide.docxLesson 1 Presentation.pptxLesson 2 Presentation.pptxLesson 3 Presentation.pptxLesson 1 Clinical Endocrine Checklist.docxLesson 2 Clinical Endocrine Checklist.docxLesson 3 Clinical Endocrine Checklist.docxLesson 1 SP Case.docxLesson 2 SP Case.docxLesson 3 SP Case.docxPre-Post Confidence Survey.docxPre-Post Spanish Endocrine Test.docxOSCE SP Diabetic Case.docxOSCE Door Note.docxOSCE Clinical Checklist Diabetic Encounter.docxOSCE Language Rubric for Diabetic Encounter.docx [file mep_2374-8265.11456-s001.zip › O. OSCE Clinical Checklist Diabetic Encounter.docx]

**Appendix O**. Clinical Checklist Diabetic Encounter OSCE

Name of Student ______________________________ Date Completed:

| ***Setting the stage of the encounter*** | **NOT Performed** | **Incompletely Performed** | **Performed** |
| --- | --- | --- | --- |
| 1. Greetings and introduction (introduces self with the name and as a medical student) | ❑ | ❑ | ❑ |
| 2. Verifies name and Age / DOB | ❑ | ❑ | ❑ |

| ***Pertinent questions on diabetic complications*** | **Did not perform** | **Incompletely Performed** | **Performed Moderately Well** | **Performed Exceptionally** |
| --- | --- | --- | --- | --- |
| 1. HPI (questions that rule out other causes of presenting symptoms) ❑ 2 questions minimum | ❑ | ❑ | ❑ | ❑ |
| 1. Coronary heart disease ❑ hx of MI, chest pain | ❑ | ❑ | ❑ | ❑ |
| 1. Cerebrovascular disease ❑ hx of stroke | ❑ | ❑ | ❑ | ❑ |
| 1. Peripheral artery disease ❑ claudication | ❑ | ❑ | ❑ | ❑ |
| 1. Nephropathy ❑ urinary frequency, changes | ❑ | ❑ | ❑ | ❑ |
| 1. Retinopathy ❑ visual impairment/changes | ❑ | ❑ | ❑ | ❑ |
| 1. Neuropathy ❑ stocking-glove loss of sensation | ❑ | ❑ | ❑ | ❑ |
| 1. Diabetic foot ❑ unnoticed wounds on feet | ❑ | ❑ | ❑ | ❑ |
| 1. Summarizes and checks for accuracy of information provided | ❑ | ❑ | ❑ | ❑ |

| ***Discuss diagnosis*** | **Did not perform** | **Incompletely Performed** | **Performed Moderately Well** | **Performed Exceptionally** |
| --- | --- | --- | --- | --- |
| 1. Tell patient diagnosis | ❑ | ❑ | ❑ | ❑ |
| 1. Ask about what patient understands about diagnosis | ❑ | ❑ | ❑ | ❑ |
| 1. Describes Diabetes, checks for understanding | ❑ | ❑ | ❑ | ❑ |
| ***Counsel on treatment*** | **Did not perform** | **Incompletely Performed** | **Performed Moderately Well** | **Performed Exceptionally** |
| 1. Diet ❑ illicit current diet ❑ discuss diabetes-friendly diet (e.g. food type, frequency, portion control, etc) | ❑ | ❑ | ❑ | ❑ |
| 1. Exercise ❑ illicit current exercise regimen ❑ inquire about patient’s preferred method of exercise (e.g. sports, walking, dancing) ❑ agree on plan of action with patient | ❑ | ❑ | ❑ | ❑ |
| 1. Medications ❑ Prescribe metformin ❑ provides additional info (e.g. risks, benefits, costs, moa, etc) | ❑ | ❑ | ❑ | ❑ |
| 1. Counsel patient to lower risk factors ❑ smoking cessation ❑ Blood Pressure ❑ weight loss | ❑ | ❑ | ❑ | ❑ |
| 1. Describe follow up recommendations for management/screening | ❑ | ❑ | ❑ | ❑ |

| ***Closing the encounter and professionalism*** | **Did not perform** | **Incompletely**  **Performed** | **Performed** |
| --- | --- | --- | --- |
| 1. Informs about what happens next | ❑ | ❑ | ❑ |
| 1. Asked the patient “Does he/she has any questions? Concerns?” | ❑ | ❑ | ❑ |
| 1. Demonstrated attentiveness via non-verbal body language | ❑ | ❑ | ❑ |
| 1. Used appropriate language and avoided medical jargon | ❑ | ❑ | ❑ |
| 1. Demonstrated professional behavior | ❑ | ❑ | ❑ |
| 1. Makes a logical differential diagnosis | ❑ | ❑ | ❑ |

| **Evaluator and Patient assessment of Spanish Ability** | **Language is a barrier to encounter** | **Significant effect on encounter** | **Moderate effect on encounter** | **Minor**  **effect on encounter** | **No negative effect on encounter** |
| --- | --- | --- | --- | --- | --- |
| 1. Rate the student’s speaking ability (rate, fluidity, pronunciation) | ❑ | ❑ | ❑ | ❑ | ❑ |
| 1. Rate the student’s use of medical vocabulary (quantity of words and use of follow-up questions/comments) | ❑ | ❑ | ❑ | ❑ | ❑ |
| 1. Rate the student’s ability to use grammar correctly | ❑ | ❑ | ❑ | ❑ | ❑ |
| 1. **Rate the student’s ability to understand the patient** | ❑ | ❑ | ❑ | ❑ | ❑ |
| 1. (Patient interpretation) Did the patient understand the student? | ❑ | ❑ | ❑ | ❑ | ❑ |
| 1. (Patient interpretation) Did the student demonstrate attentiveness and respect through verbal and non-verbal communication? | ❑ | ❑ | ❑ | ❑ | ❑ |

|  | **Check only one box below** |
| --- | --- |
| Using the scale below, rate the student’s ability to interact with a patient: |  |
| 0 – Doesn’t speak any Spanish | ❑ |
| 1 – Is limited to greetings and goodbyes. | ❑ |
| 2 – Can understand very common medical terminology but wouldn’t feel comfortable responding or initiating conversation in Spanish. | ❑ |
| 3 – With difficulty, can speak to patients about very common topics and common anatomy. | ❑ |
| 4 – With relative ease, can speak to patients about very common topics and common anatomy. | ❑ |
| 5 – With difficulty, can speak to patients about more intricate medical and nonmedical terminology. | ❑ |
| 6 – With relative ease, can speak to patients about more intricate medical and nonmedical terminology. | ❑ |
| 7 – With very limited help or while making clinically insignificant mistakes, can conduct an entire patient interaction (history & physical). | ❑ |
| 8 – Can conduct an entire patient interaction without the aid of a translator. | ❑ |
| 9 – I consider this student a fluent Spanish-speaker. | ❑ |

| ***Narrative Feedback (Comment on the overall performance – communication skills, examination skills, technique)*** |
| --- |
|  |
