# Supplementary material for: Integrating Gender-Affirming Care in a Medical Spanish Endocrine System Curriculum
Source: MedEdPORTAL. 2024 Oct 23;20:11456. doi: 10.15766/mep_2374-8265.11456 (PMC11496385; doi:10.15766/mep_2374-8265.11456)
Supplement: Supplementary file 1 — Facilitator Guide.docxLesson 1 Presentation.pptxLesson 2 Presentation.pptxLesson 3 Presentation.pptxLesson 1 Clinical Endocrine Checklist.docxLesson 2 Clinical Endocrine Checklist.docxLesson 3 Clinical Endocrine Checklist.docxLesson 1 SP Case.docxLesson 2 SP Case.docxLesson 3 SP Case.docxPre-Post Confidence Survey.docxPre-Post Spanish Endocrine Test.docxOSCE SP Diabetic Case.docxOSCE Door Note.docxOSCE Clinical Checklist Diabetic Encounter.docxOSCE Language Rubric for Diabetic Encounter.docx [file mep_2374-8265.11456-s001.zip › P. OSCE Language Rubric for Diabetic Encounter.docx]

**Appendix P.** Language Rubric for Diabetic Encounter OSCE

| **Student Name** |  |  |  |  |  |
| --- | --- | --- | --- | --- | --- |
|  | **Understanding (0-100%)** *Ability to comprehend patient*’*s histories, instructions, and questions* | **Expressiveness (0-100%)** *Capability to communicate medical advice, diagnoses, and convey empathy* | **Medical Terminology (0-100%)** *Appropriate and accurate use of medical vocabulary in context* | **Grammar & Structure (0-100%)** *Use of correct language structure and syntax in communication* |  |
| 10% | The student doctor has extremely limited understanding. Recognizes only a few isolated words or phrases, with no grasp of the overall meaning. | The student doctor can barely communicate basic medical advice or feelings. Expressions are limited to isolated words or phrases, showing almost no ability to convey empathy. | The student doctor uses medical terms incorrectly or inappropriately in every instance, leading to complete miscommunication | The student doctor consistently constructs sentences incorrectly, with severe grammatical errors that lead to widespread misunderstanding and ineffective communication. |  |
| 20% | The student doctor demonstrates a clearly insufficient, level of understanding and is occasionally able to identify some basic ideas or instructions with significant errors. | The student doctor struggles to form basic sentences. Communication of medical advice and empathy is extremely limited and often misunderstood. | The student doctor occasionally uses basic medical terms correctly but often inaccurately, leading to confusion. | The student doctor frequently makes fundamental grammatical mistakes, significantly impeding clear communication and often leading to confusion in medical discussions. |  |
| 30% | The student doctor shows a fragmented understanding, with only occasional and vague recognition of key details. Clarity is rare, and understanding is inconsistent. | The student doctor communicates simple medical advice with difficulty. Attempts at expressing empathy are present but often fail to convey the intended emotion or support. | The student doctor uses some medical terms correctly but struggles with accuracy and appropriateness in context. | The student doctor struggles with basic grammar and sentence structure, leading to frequent errors that obstruct clear communication in medical contexts. |  |
| 40% | The student doctor demonstrates understanding intermittently, with a sporadic grasp of key details. There are moments of clarity, but understanding is not consistent or reliable. | The student doctor can express basic medical advice and diagnoses. Shows attempts at empathy, but often lacks clarity and effectiveness in emotional expression. | The student doctor correctly uses basic medical terms intermittently; errors in terminology are common and affect communication. | The student doctor demonstrates some grasp of grammar and structure, but frequent errors and incorrect sentence formations hinder effective communication in medical scenarios. |  |
| **50%** | The student doctor exhibits a basic level of understanding, frequently grasping key details. While there are still misunderstandings, moments of clarity and accurate comprehension occur regularly. | The student doctor expresses medical advice and diagnoses in simple terms. Demonstrates a basic level of empathy, with some ability to connect emotionally with patients | The student doctor generally uses basic medical terms correctly; some inaccuracies in complex terminology or context. | The student doctor shows basic competence in grammar and sentence structure, with consistent but less severe errors that occasionally affect clarity in medical exchanges. |  |
| **60%** | The student doctor has a moderate understanding, regularly grasping key details and concepts. Misunderstandings are less frequent, and there is a consistent level of clarity in comprehension. | The student doctor communicates medical advice and diagnoses clearly. Shows a fair level of empathy, able to convey understanding and concern to the patient. | The student doctor uses medical terms accurately in familiar contexts; occasional errors in complex situations. | The student doctor generally uses correct grammar and structure, with occasional errors that do not significantly impair communication in medical settings. |  |
| **70%** | The student doctor demonstrates a good level of understanding, with occasional misunderstandings. Key details are usually well grasped. | The student doctor effectively communicates detailed medical advice and diagnoses. Demonstrates good empathetic engagement, often connecting well with patients. | The student doctor accurately uses a wide range of medical vocabulary; rarely makes errors, even in complex medical contexts. | The student doctor demonstrates good grammatical and structural control, with minor errors that rarely affect the overall effectiveness of communication in medical encounters. |  |
| 8**0%** | The student doctor shows a strong, with misunderstandings being rare. Key details and concepts are consistently grasped, and there is a high level of clarity and reliability in comprehension. | The student doctor conveys medical advice and diagnoses fluently and accurately. Shows strong empathy, consistently demonstrating understanding and support to patients. | The student doctor consistently uses medical terms accurately and appropriately, with very few errors, even in advanced medical discussions. | The student doctor exhibits a strong command of grammatical and sentence structure, making few errors and effectively communicating complex medical information clearly and accurately. |  |
| **90%** | The student doctor exhibits near-complete understanding, with only minimal misunderstandings. Almost all key details and concepts are grasped with exceptional clarity, showing a deep and consistent understanding. | The student doctor communicates complex medical advice and diagnoses with ease. Exhibits a high level of empathy, skillfully conveying compassion and understanding. | The student doctor exhibits near-perfect use of medical vocabulary; errors are extremely rare and minor, with excellent contextual accuracy. | The student doctor uses grammar and structure very effectively, with rare minor errors that do not impede understanding, facilitating smooth and clear communication in medical contexts. |  |
| **100%** | The student doctor exhibits complete and consistent understanding. Every aspect of patient dialogue, from symptoms to treatment discussions, is understood accurately, with no misinterpretations. | The student doctor exhibits exceptional ability to communicate comprehensive medical advice and diagnoses. Demonstrates profound empathy, establishing deep and meaningful connections with patients, ensuring clear understanding and emotional support. | The student doctor uses medical vocabulary with complete accuracy and appropriateness across all contexts, demonstrating mastery in medical terminology. | The student doctor demonstrates masterful control of grammar and sentence structure, communicating complex medical information seamlessly and flawlessly, with no discernible errors, ensuring precise and clear understanding in all medical encounters. |  |
|  |  |  |  |  | **TOTAL** *Average of each category* |
