# Supplementary material for: Integrating Gender-Affirming Care in a Medical Spanish Endocrine System Curriculum
Source: MedEdPORTAL. 2024 Oct 23;20:11456. doi: 10.15766/mep_2374-8265.11456 (PMC11496385; doi:10.15766/mep_2374-8265.11456)
Supplement: Supplementary file 1 — Facilitator Guide.docxLesson 1 Presentation.pptxLesson 2 Presentation.pptxLesson 3 Presentation.pptxLesson 1 Clinical Endocrine Checklist.docxLesson 2 Clinical Endocrine Checklist.docxLesson 3 Clinical Endocrine Checklist.docxLesson 1 SP Case.docxLesson 2 SP Case.docxLesson 3 SP Case.docxPre-Post Confidence Survey.docxPre-Post Spanish Endocrine Test.docxOSCE SP Diabetic Case.docxOSCE Door Note.docxOSCE Clinical Checklist Diabetic Encounter.docxOSCE Language Rubric for Diabetic Encounter.docx [file mep_2374-8265.11456-s001.zip › _Educational Summary Report_11456.pdf]

MedEdPORTAL® The AAMC Journal of Teaching and Learning Resources

Original Publication Open Access

Integrating Gender-Affirming Care in a Medical Spanish Endocrine System Curriculum

Alexandra Lopez Vera, PhD, MPH*, Joshua Ahmad, Catania Ramos, Katrina Kao, Alexander Flores

*Corresponding author: alexandra.lopezvera@cusm.edu

Abstract

Introduction: With a growing Hispanic population in the United States, medical education is adapting to provide the necessary language skills and cultural competence for effective health care. However, the incorporation of gender-affirming care in the context of medical education for Hispanic populations requires further emphasis. **Methods:** This curriculum presented a 3-week medical Spanish endocrine system module designed for first-year medical students. The module aimed to enhance students' ability to communicate effectively with Spanish-speaking patients about endocrine health while integrating principles of gender-affirming care. It included classroom sessions, standardized patient practice, and clinical practice with peer tutors. Pre- and postmodule surveys and assessments were conducted to evaluate the module's effectiveness. **Results:** Out of 76 participants, 72 completed the postmodule evaluation. Survey results indicated significant increases in confidence levels across various aspects of patient interaction in Spanish, with statistically significant gains observed in all assessed areas. Knowledge test outcomes revealed enhanced proficiency in Spanish terminology related to the endocrine system, with scores increasing from an average of 22.3 premodule to 25.7 postmodule ($p = .002$), as measured by the paired t test. Additionally, students performed well in the diabetic consultation objective structured clinical examination station, with a high mean score of 86%, surpassing the satisfactory threshold. **Discussion:** This curriculum highlights the success of a comprehensive educational approach in expanding medical students' language proficiency and ability to provide gender-affirming care to address health care disparities and improve patient outcomes among diverse populations.

Keywords
Gender-Affirming Language, Case-Based Learning, Endocrine System, Medical Spanish, Standardized Patient, Language-Appropriate Health Care

Educational Objectives

By the end of this module, medical students will be able to:

1. Demonstrate the application of gender-affirming care principles in Spanish when managing endocrine disorders for Spanish-speaking patients, showcasing sensitivity to their unique health needs through role-play scenarios and standardized patient interactions.
2. Comprehend and explain the functions of the endocrine system in Spanish, as demonstrated through written assessments.
3. Utilize specific Spanish medical vocabulary and grammar effectively to conduct comprehensive medical histories for patients with diabetes, recognizing and addressing the intersection of endocrine health and gender identity in clinical practice during standardized patient encounters and assessments.

Citation:
Lopez Vera A, Ahmad J, Ramos C, Kao K, Flores A. Integrating gender-affirming care in a medical Spanish endocrine system curriculum. *MedEdPORTAL.* 2024;20:11456.
https://doi.org/10.15766/mep_2374-8265.11456

Introduction

With a growing Hispanic population estimated to reach 24% in the United States by 2050, the demand for Spanish-speaking physicians is steadily increasing.[1] Although patients who speak languages different from English can access language assistance through interpreters in their native tongue, this approach is not preferred when patients need to communicate sensitive information to their health care providers.[2] To address this need, many medical schools now offer medical Spanish education programs aimed at equipping future physicians with essential communication skills for effective patient–physician

Copyright © 2024 Lopez Vera et al. This is an open-access publication distributed under the terms of the Creative Commons Attribution license.

interactions.[3] However, due to the relatively recent development of such curricula, there is significant variation in their delivery among institutions. These programs employ diverse teaching modalities, including didactic instruction, role-play, standardized patients (SPs), and immersion, and use various evaluation methods, such as oral exams, written exams, objective structured clinical examinations (OSCEs), attendance and participation assessments, and self-assessment tools.[4] The most common barriers to the effectiveness of medical Spanish curricula include a lack of time, varied student language proficiency levels, the cost of running the course, and lack of student retention.[1] Using a systems-based approach allows for focused Spanish language learning and augmented medical systems reinforcement. One study demonstrated that learners benefited from studying one subject concurrently in both languages—English first, then Spanish—and showed improvement in vocabulary, grammar, comprehension, and self-confidence in a Spanish system-based musculoskeletal and dermatological module.[5] Furthermore, assessments conducted before and after the course focusing on vocabulary, grammar, and comprehension related to musculoskeletal and dermatologic health revealed that all 47 participants increased their scores across all categories, with students reporting higher confidence in patient interviews and examinations concerning musculoskeletal and dermatologic diseases.[5] Another study, which aimed to gauge students' acceptance of medical Spanish training, found that following the course, more than half the students said clinical Spanish should be mandatory and that a significant majority indicated the content they had learned was valuable and important and enhanced their clinical experiences.[6]

While the expansion of medical training in the United States to include Spanish language training is essential, there is a pressing need to integrate endocrine-specific content, particularly as it relates to the care of transgender patients, into the medical Spanish curriculum.[7] Transgender patients often encounter unique health challenges, including hormonal treatment complexities, which are integral aspects of endocrine care.[8] While gender-affirming care remains a critical principle, addressing the endocrine system's role is essential for providing comprehensive care to transgender patients. Health care providers must be equipped with not only the knowledge of inclusive care practices and terminology but also a deep understanding of the endocrine system to effectively treat and support the transgender population.[9] Recent studies indicate that medical students feel unprepared to manage the specific health needs of transgender patients, highlighting a gap in current medical education.[8] Although advancements in medical Spanish programs have facilitated improved communication with Spanish-speaking patients, the incorporation of endocrine-related content, specifically tailored for transgender patient care, is often overlooked.[10]

Meeting the needs of the LGBTQ+ community in health care requires substantial efforts from medical training institutions to educate students on providing compassionate, inclusive care.[11] A systematic review of literature from 2000 to 2020 focusing on gender-affirming care training screened 27,090 articles and identified only 36 that concentrated on educational interventions for providing health care to LGBTQ+ patients.[12] Importantly, many of these articles originated from medical schools rather than residency or other training institutions,[12] underscoring the significant gap in medical education at various levels concerning the preparation of future health care providers to address the needs of their patients. Although substantial curriculum enhancements should be the ultimate goal, the incorporation of small lessons on correct pronoun use is a critical initial step. Research has shown that gender affirmation through the appropriate use of pronouns has been associated with improved patient affect, empowerment, and hormone use, particularly in the context of HIV treatment for transgender women.[13] The absence of metrics to quantify pronoun importance to transgender women was addressed by a study that created the Transgender Women's Importance of Pronouns scale, which demonstrated promise as a helpful measure in clinical and research settings.[13]

Furthermore, given the high prevalence of diabetes and other endocrine disorders in the Hispanic population, there is a pressing need for medical professionals who can navigate these complex conditions in Spanish, ensuring culturally and linguistically competent care. In fact, the endocrine system disorders, including diabetes, hold unique significance for transgender and gender-nonconforming individuals. Studies have documented that transgender individuals, especially those undergoing hormone therapy, may have an altered risk profile for endocrine-related conditions such as diabetes. This relationship emphasizes the importance of medical professionals being adept in both endocrinology and gender-affirming care to address potential health disparities effectively.[14]

Methods

Context

Building on the success of the medical Spanish endocrinology educational module at the University of Illinois College of Medicine,[15] we developed a 3-week medical Spanish endocrine

Copyright © 2024 Lopez Vera et al. This is an open-access publication distributed under the terms of the Creative Commons Attribution license.

system module for first-year medical students at the California University of Science and Medicine (CUSM). This module was designed with the objective of providing students with the essential understanding and practical abilities required to effectively communicate with Spanish-speaking patients regarding endocrine health and related conditions. A total of 76 first-year medical students participated in the initiative, providing a substantial dataset for analyzing the module's effectiveness. Out of 76 participants who began the medical Spanish endocrine system module, 72 completed the postmodule evaluation, yielding a response rate of 95%. Expert input from medical Spanish educators, endocrinologists, and LGBTQ+ health specialists shaped our module, integrating gender-affirming care, transgender hormone therapy, and inclusive language.

Each week, students attended a 50-minute lecture presentation, followed by a 25-minute practice session with SPs and a 60-minute session with peer tutors. Additionally, the module required students to engage in self-study to enhance their language skills and deepen their understanding of the endocrine system. SPs were hired on a rolling basis and trained by an SP educator before each session to ensure they were well prepared for their roles in the module.

To enroll in this module, students needed to be participants in the medical Spanish program at CUSM, known as Vida.[10] The endocrine-related topics covered in the module were integral components of the core system–based curriculum within the MD degree program at CUSM. The facilitators for this module, including the director of the medical Spanish program and peer tutors, were required to either be native Spanish speakers or possess an advanced Spanish proficiency level according to the American Council on the Teaching of Foreign Languages.[16] Furthermore, the course director had to have linguistic or medical training at the PhD or MD level to meet the essential standards for medical Spanish education.

Learning Environment Setup

To create an ideal educational environment that promoted student engagement and active learning, we conducted large-group sessions in a well-equipped classroom. This classroom featured a computer connected to multiple screens, facilitating the display of presentations throughout the room. In addition, microphones were provided to ensure clear communication between the instructor and students. Attendees were encouraged to use the microphones to ask questions or respond during the session.

Module Breakdown

The 3-week module comprised three key components: (1) Each week commenced with a 3-hour self-study session assigned the previous week. (2) This was followed by a 75-minute classroom session where students received instruction from medical Spanish faculty members for 50 minutes and engaged in practice with SPs for 25 minutes. (3) Additionally, a 1-hour weekly clinical practice session was conducted with peer tutors. To ensure the smooth implementation of the module, we provided a comprehensive set of module materials, including a facilitator guide (Appendix A), PowerPoint presentations for the three instructor-led lessons (Appendices B-D), clinical endocrine checklists for the three lessons (Appendices E-G), and scripts for the SPs (Appendices H-J).

The module aimed to enhance learners' cultural competence and oral communication skills in the field of endocrinology. Before each week's class, students were assigned readings on gender-affirming cultural information, thoughtfully selected to provide a comprehensive understanding of the topic.[17-19] Additionally, students were directed to review the target grammar structure for the week in their provided Spanish textbook during this preclass self-study period, totaling 3 hours. This preparation was considered essential for preparing students adequately for the upcoming week's content. However, it was voluntary, and there was no formal documentation of students completing their assigned homework.

Each classroom session was conducted in Spanish by the program director. These sessions covered endocrine system topics, including vocabulary and general medical information. The aim was to reinforce clinical case studies and clinical skills sessions by introducing endocrine-related content in Spanish a week after it had been covered in the core system–based curriculum. The classroom sessions also included an implicit review of the weekly target grammar structure, reinforcing the concepts introduced in the assigned readings and facilitating language use in a medical context. Gender-affirming activities were integrated into these sessions to engage students with the cultural aspects of the topic. To ensure comprehensive coverage of endocrine system–related scenarios, our educational module was structured to include specific weeks dedicated to different topics. Diabetes-related scenarios were introduced and discussed during the first week, while scenarios involving nonbinary patients were addressed in the second and third weeks.

During the classroom sessions, discussions on relevant anatomy and guided questions addressing chief complaints related to the

Copyright © 2024 Lopez Vera et al. This is an open-access publication distributed under the terms of the Creative Commons Attribution license.

endocrine organ system occurred. These discussions provided a platform for students to practice their oral communication skills in Spanish, with English translation support available in the presentations.

Following the classroom sessions, students separated into small groups of four to six individuals. Each group worked with an SP who had been provided with a script in advance. These scripts covered various endocrine system–related chief complaints, including scenarios involving nonbinary patients. SPs had undergone training provided by an SP educator to ensure their effectiveness in fulfilling their roles. The primary focus of these encounters was to develop students' real-time oral communication skills by simulating patient interactions and emphasizing effective verbal information conveyance. Customized scripts allowed learners to apply their knowledge and skills to real-world clinical situations, enhancing their preparedness and confidence. Learners typically had 20 minutes for SP practice. A peer tutor was present at every SP encounter to provide verbal feedback at the end, ensuring continuous improvement and skill development.

Our SP pool included members of the LGBTQ community who had provided insights into their experiences to both the medical Spanish program director and the SP educator. Moreover, the program director had received training in Spanish language affirming practices. This ensured that the SP encounters were conducted in a sensitive and respectful manner, incorporating principles of inclusive care into the interactions.

Evaluation

To assess students' confidence in using gender-affirming language in Spanish, all participants in this curriculum were asked to complete a presurvey and postsurvey (Appendix K). Additionally, students were required to complete a preassessment and postassessment test to evaluate their proficiency in the Spanish endocrine system domain (Appendix L). The test featured 20 questions in various formats, including multiple choice, free response, and fill in the blank, aimed at assessing comprehension and the ability to communicate effectively in the field of endocrinology. The presurvey and preassessment were administered the week before the start of the endocrine module, whereas the postsurvey and postassessment occurred a week after the module's completion.

Finally, at the end of the preclerkship curriculum, out of the 72 students who completed the module, 61 also completed a medical Spanish OSCE. This OSCE was specifically crafted to evaluate the integration of Spanish language skills in clinical interactions, aligning with the structure of the clinical skills courses at CUSM, and entailed interactions with trained SPs. The assessment comprised three stations: neurological examination, comprehensive history taking, and diabetes counseling, each lasting 25 minutes. Notably, the diabetes counseling station featured a patient who identified with nonbinary pronouns (Appendix M). The door note for this encounter was provided to students in English (Appendix N). Results from this station were analyzed using a two-level approach. Initially, bilingual clinical student doctors were the graders and used standardized checklists derived from *Bates' Guide to Physical Examination and History Taking* (Appendix O).[20] The checklist's scoring system was validated by clinical skills and medical Spanish faculty and awarded 0 points for actions not performed, 1 point for actions executed with errors, and 2 points for actions completed with minimal errors.[20] To ensure consistency in grading, an interrater reliability assessment was carried out. The course director of the medical Spanish program reviewed each encounter and utilized a language rubric created for the program (Appendix P) to demonstrate the effectiveness of our medical Spanish endocrine system module. Final grades were determined by calculating the average of both assessments. For the purpose of this publication, we have focused on the single endocrine station to highlight specific results related to the module. However, it was not the sole assessment method of the course and was never intended to singularly assess the effectiveness of the entire course. This approach was designed to show targeted outcomes related to the endocrine system component within a broader multistation assessment framework (Figure 1).

The project was approved by the institutional review board of the California University of Science and Medicine, under protocol # HS-2023-21, dated April 27, 2023.

Results

Survey Findings

The survey results are presented in Figure 2, where the *y*-axis represents the average confidence level and the *x*-axis represents the selected questions. Four survey questions were asked before and after the module, and participants rated their confidence on a 5-point scale (1 = *not confident at all*, 5 = *extremely confident*). For the analysis, we used Microsoft Excel version 16.84, and a *t* test was employed to determine statistical significance. Out of 76 participants, 72 completed the postmodule evaluation (presurvey: $n = 76$, postsurvey: $n = 72$). For question 1, which assessed students' confidence in their ability to assist patients with endocrine problems, there

Copyright © 2024 Lopez Vera et al. This is an open-access publication distributed under the terms of the Creative Commons Attribution license.

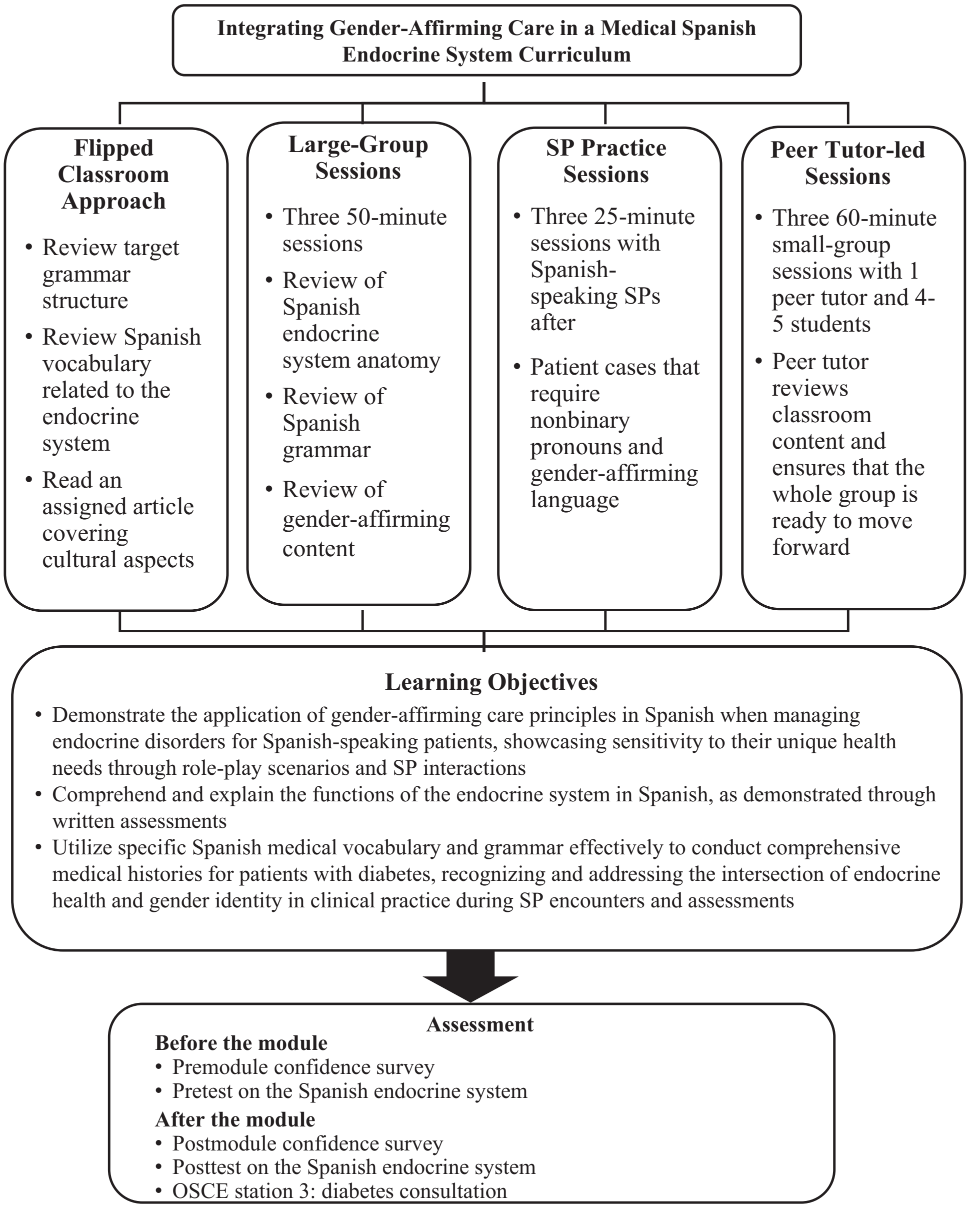

Figure 1. Integrating gender-affirming care in a medical Spanish endocrine system curriculum. The figure illustrates the components of the curriculum designed to incorporate gender-affirming care principles into the teaching of medical Spanish for endocrine health. Abbreviations: OSCE, objective structured clinical examination; SP, standardized patient.

was a significant increase in self-reported confidence levels, rising from an average of 2.1 before the module to 3.5 after completing it. For question 2, which evaluated students' ability to explain the endocrine system in Spanish, average scores rose from 1.9 before the module to 3.3 afterward, indicating enhanced confidence in their descriptive communication. On question 3, focusing on the use of specific Spanish medical vocabulary and grammar for taking histories of diabetes patients, there was an increase in average confidence scores from 2.2 to 3.4. Furthermore, question 4's results, addressing the integration of inclusive language in interactions with endocrine patients, showed an improvement from 2.3 to 3.7. All survey questions demonstrated statistically significant gains in confidence levels ($p < .05$), underlining the effectiveness of the module in boosting students' ability to communicate effectively and empathetically with Spanish-speaking endocrine patients.

Copyright © 2024 Lopez Vera et al. This is an open-access publication distributed under the terms of the Creative Commons Attribution license.

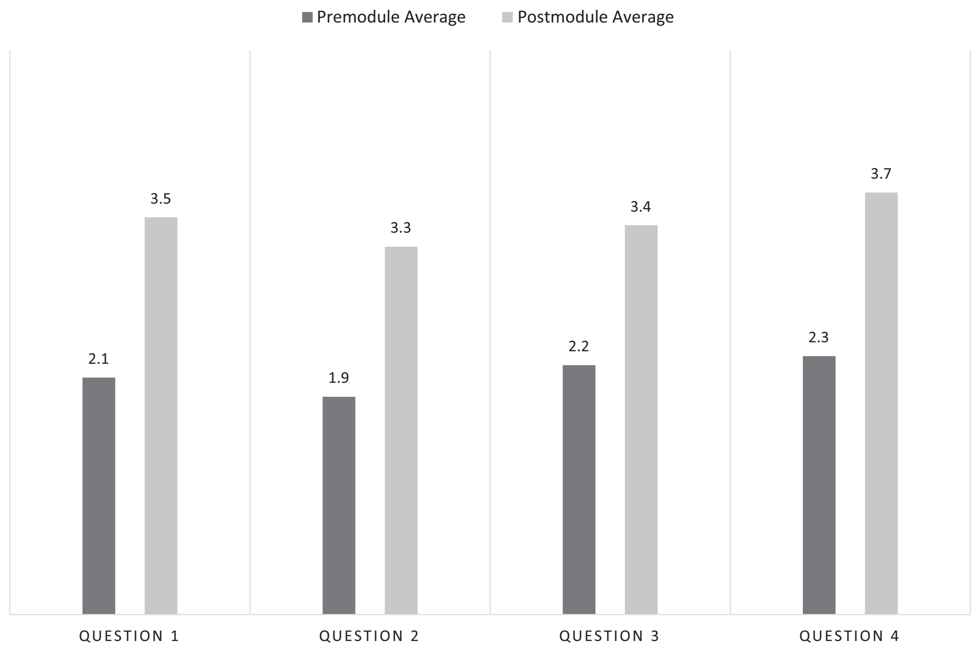

Figure 2. Confidence survey. Results indicate significant increases in confidence levels across various aspects of patient interaction in Spanish, with statistically significant gains observed in all assessed areas.

Knowledge Test Outcomes

The test, administered in Spanish, consisted of 30 questions and assessed students' knowledge of Spanish terminology related to the endocrine system. Students had 20 minutes to complete the test, which was conducted using Microsoft Forms with real-time data collection. As depicted in Figure 3, knowledge test outcomes revealed enhanced proficiency in Spanish terminology related to the endocrine system, with scores increasing from an average of 22.3 premodule to 25.7 postmodule ($p = .002$), as measured by the paired t test (presurvey: $n = 76$, postsurvey: $n = 72$). Students took an average of 10 minutes and 21 seconds to complete the survey.

Diabetic Consultation OSCE Station Results

The OSCE results, depicted in Figure 4, reveal a high mean score of 86%, indicative of students' competency above the satisfactory threshold (70%). Out of the 72 students who completed the module, 61 also completed the medical Spanish OSCE. With a low standard deviation (10%), consistency in performance across the cohort suggests effective attainment of learning outcomes. The high mean score and low standard deviation indicate consistent performance across the cohort, with few low performers, suggesting widespread comprehension, while a left-skewed grade distribution highlights most students achieving higher grades (80%-100%).

Discussion

In the development and implementation of our medical Spanish curriculum focusing on the endocrine system, we proved the effectiveness of targeted medical language training, aligning with existing literature.[14] Several articles have provided evidence of the effectiveness of teaching medical Spanish vocabulary to medical students.[5,12] Our curriculum's strengths stem from the collaborative efforts in its design and the administration of surveys and tests to all participants at two points: before and after the 3-week module. This tightly controlled time frame ensured that improvements in self-rated confidence surveys and test scores were solely attributable to the education provided and not influenced by external factors. In addition, the increased time taken for the postmodule test suggests deeper engagement and a more comprehensive understanding of the material, as students may have taken the additional time to carefully consider their responses or review their understanding of certain concepts before completing the postmodule test. Finally, the OSCE results indicate widespread competency among students, with a high average score of 86% and a low standard deviation of 10%, highlighting the effectiveness of the module in imparting essential knowledge and skills.

However, we encountered limitations in controlling for participants' preexisting knowledge of the endocrine system and

Copyright © 2024 Lopez Vera et al. This is an open-access publication distributed under the terms of the Creative Commons Attribution license.

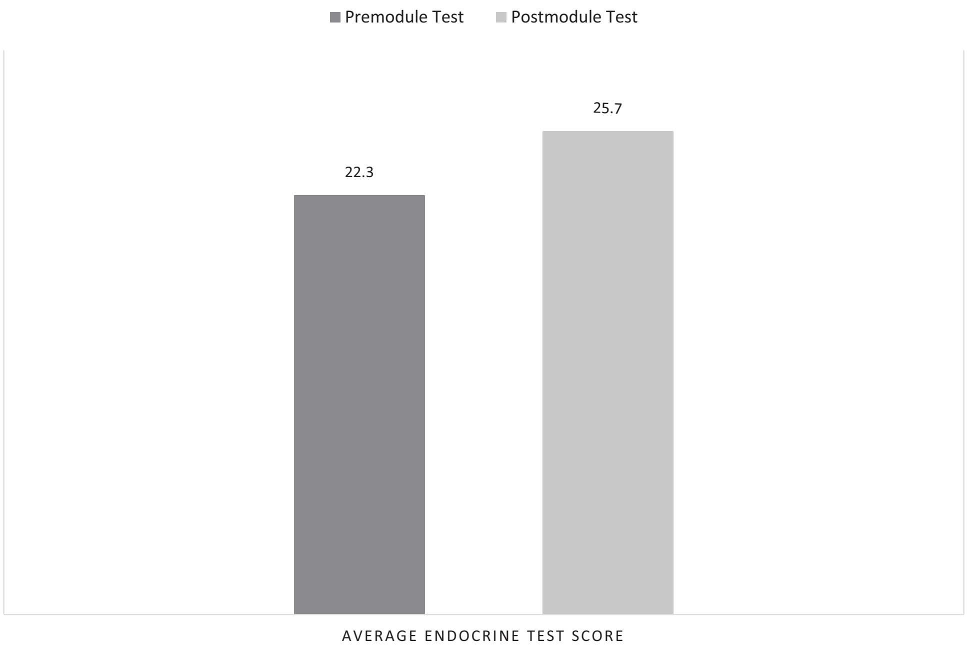

Figure 3. Endocrine test. Pre- and postmodule test outcomes reveal enhanced proficiency in Spanish terminology related to the endocrine system, with a substantial increase in average scores postmodule.

phonetic similarities between endocrine vocabulary in English and Spanish that could have affected test results. Additionally, because attendance at large- and small-group sessions was not mandatory, we could not guarantee consistent attendance, potentially affecting the sample size and composition for pre- and posttests. Potential approaches for students to document this assigned activity could include offering extra credit points to encourage participation.

Our results demonstrate significant improvement in students' knowledge of medical Spanish terms related to the human endocrine system after repeated exposure and comprehensive

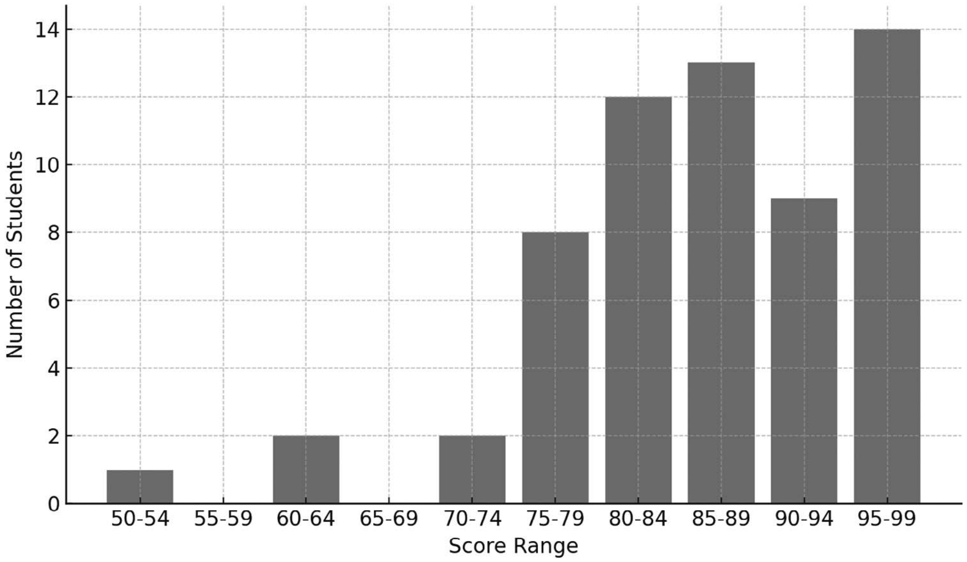

Figure 4. Medical Spanish objective structured clinical examination (OSCE) results—endocrine station. The outcomes of the OSCE for the medical Spanish module, focusing on the endocrine station, showcase students' competency above the satisfactory threshold with a high mean score.

Copyright © 2024 Lopez Vera et al. This is an open-access publication distributed under the terms of the Creative Commons Attribution license.

study in both large and small groups and through individual effort. These findings affirm the effectiveness of our educational module in expanding medical students' Spanish vocabulary for treating endocrine system disorders and in promoting inclusive communication with marginalized communities. Equipped with these skills, the participants students are better prepared to provide effective health care.

Additionally, our experience highlighted the complexities of integrating academic schedules with the demanding and diverse commitments of medical students, prompting a reevaluation of how we encourage and facilitate participation in educational sessions. The positive feedback and measurable learning gains from the module demonstrate its value and effectiveness, yet these insights have been essential for planning future curriculum enhancements. The lessons learned from implementing this module will inform our efforts to develop and refine subsequent modules for other body systems, aiming for a curriculum that is not only academically rigorous but also adaptable and accessible to a diverse student body.

As we move forward, our focus will be on expanding the curriculum to encompass a wider range of medical specialties and integrating comprehensive care principles, including LGBTQ+ education. This initiative will require continuous evaluation and adaptation to ensure that we are meeting the educational needs of our students while preparing them for the linguistic and cultural challenges of health care delivery. By sharing our experiences and the nuanced challenges we have faced, we aim to contribute to the broader dialogue on medical Spanish education and provide insights and practical guidance for institutions seeking to implement or enhance their own programs.

Appendices

A. Facilitator Guide.docx
B. Lesson 1 Presentation.pptx
C. Lesson 2 Presentation.pptx
D. Lesson 3 Presentation.pptx
E. Lesson 1 Clinical Endocrine Checklist.docx
F. Lesson 2 Clinical Endocrine Checklist.docx
G. Lesson 3 Clinical Endocrine Checklist.docx
H. Lesson 1 SP Case.docx
I. Lesson 2 SP Case.docx
J. Lesson 3 SP Case.docx
K. Pre-Post Confidence Survey.docx
L. Pre-Post Spanish Endocrine Test.docx
M. OSCE SP Diabetic Case.docx
N. OSCE Door Note.docx
O. OSCE Clinical Checklist Diabetic Encounter.docx
P. OSCE Language Rubric for Diabetic Encounter.docx

All appendices are peer reviewed as integral parts of the Original Publication.

Alexandra Lopez Vera, PhD, MPH: Assistant Professor, Department of Medical Education, California University of Science and Medicine; ORCID: https://orcid.org/0000-0001-7659-3672

Joshua Ahmad: Third-Year Medical Student, California University of Science and Medicine

Catania Ramos: Third-Year Medical Student, California University of Science and Medicine

Katrina Kao: Third-Year Medical Student, California University of Science and Medicine

Alexander Flores: Third-Year Medical Student, California University of Science and Medicine

Disclosures
None to report.

Funding/Support
None to report.

Ethical Approval
The California University of Science and Medicine Institutional Review Board deemed further review of this project not necessary.

References

1. Morales R, Rodriguez L, Singh A, et al. National survey of medical Spanish curriculum in U.S. medical schools. *J Gen Intern Med*. 2015;30(10):1434-1439. https://doi.org/10.1007/s11606-015-3309-3
2. Lopez Vera A, Thomas K, Trinh C, Nausheen F. A case study of the impact of language concordance on patient care, satisfaction, and comfort with sharing sensitive information during medical care. *J Immigr Minor Health*. 2023;25(6):1261-1269. https://doi.org/10.1007/s10903-023-01463-8
3. Hardin KJ, Hardin DM Jr. Medical Spanish programs in the United States: a critical review of published studies and a proposal of best practices. *Teach Learn Med*. 2013;25(4):306-311. https://doi.org/10.1080/10401334.2013.827974

Copyright © 2024 Lopez Vera et al. This is an open-access publication distributed under the terms of the Creative Commons Attribution license.

4. Ortega P, Pérez N, Robles B, Turmelle Y, Acosta D. Teaching medical Spanish to improve population health: evidence for incorporating language education and assessment in U.S. medical schools. *Health Equity*. 2019;3(1):557-566. https://doi.org/10.1089/heq.2019.0028

5. Ortega P, López-Hinojosa I, Park YS, Girotti JA. Medical Spanish musculoskeletal and dermatologic educational module. *MedEdPORTAL*. 2021;17:11071. https://doi.org/10.15766/mep_2374-8265.11071

6. Pérez NA. Student acceptance of clinical conversational Spanish in medical curriculum. *Cogent Med*. 2018;5(1):1475691. https://doi.org/10.1080/2331205X.2018.1475691

7. Hana T, Butler K, Young LT, Zamora G, Lam JSH. Transgender health in medical education. *Bull World Health Organ*. 2021;99(4):296-303. https://doi.org/10.2471/BLT.19.249086

8. Bhatt N, Cannella J, Gentile JP. Gender-affirming care for transgender patients. *Innov Clin Neurosci*. 2022;19(4-6):23-32.

9. Lee JY, Rosenthal SM. Gender-affirming care of transgender and gender-diverse youth: current concepts. *Annu Rev Med*. 2023;74:107-116. https://doi.org/10.1146/annurev-med-043021-032007

10. Chang KE, Lewis J, Lopez Vera A. A comprehensive medical Spanish curriculum model: the Vida Medical Spanish Curriculum. *BMC Med Educ*. 2023;23:488. https://doi.org/10.1186/s12909-023-04473-0

11. Bass B. Cultural competence in the care of LGBTQ patients. *StatPearls*. Updated November 13, 2023. Accessed October 2, 2024. https://www.statpearls.com/point-of-care/125326

12. Juarez PD, Ramesh A, Reuben JS, et al. Transforming medical education to provide gender-affirming care for transgender and gender-diverse patients: a policy brief. *Ann Fam Med*. 2023;21(suppl 2):S92-S94. https://doi.org/10.1370/afm.2926

13. Sevelius JM, Chakravarty D, Dilworth SE, Rebchook G, Neilands TB. Gender affirmation through correct pronoun usage: development and validation of the Transgender Women's Importance of Pronouns (TW-IP) scale. *Int J Environ Res Public Health*. 2020;17(24):9525. https://doi.org/10.3390/ijerph17249525

14. T'Sjoen G, Arcelus J, Gooren L, Klink DT, Tangpricha V. Endocrinology of transgender medicine. *Endocr Rev*. 2019;40(1):97-117. https://doi.org/10.1210/er.2018-00011

15. Ortega P, González C, López-Hinojosa I, Park YS, Girotti JA. Medical Spanish endocrinology educational module. *MedEdPORTAL*. 2022;18:11226. https://doi.org/10.15766/mep_2374-8265.11226

16. *ACTFL Proficiency Guidelines 2012*. American Council on the Teaching of Foreign Languages; 2012. Accessed October 2, 2024. https://www.actfl.org/uploads/files/general/ACTFLProficiencyGuidelines2012.pdf

17. Aguayo-Mazzucato C, Diaque P, Hernandez S, Rosas S, Kostic A, Caballero AE. Understanding the growing epidemic of type 2 diabetes in the Hispanic population living in the United States. *Diabetes Metab Res Rev*. 2019;35(2):e3097. https://doi.org/10.1002/dmrr.3097

18. *Providing Affirmative Care for Patients With Non-binary Gender Identities*. National LGBT Health Education Center; date unknown. Accessed October 2, 2024. https://www.lgbtqiahealtheducation.org/wp-content/uploads/2017/02/Providing-Affirmative-Care-for-People-with-Non-Binary-Gender-Identities.pdf

19. Abreu RL, Gonzalez KA, Mosley DV, Pulice-Farrow L, Adam A, Duberli F. "They feel empowered to discriminate against las chicas": Latina transgender women's experiences navigating the healthcare system. *Int J Transgend Health*. 2022;23(1-2):178-193. https://doi.org/10.1080/26895269.2020.1767752

20. Bickley LS. *Bates' Guide to Physical Examination and History Taking*. 8th ed. Lippincott Williams & Wilkins; 2003.

Received: November 13, 2023
Accepted: June 21, 2024
Published: October 23, 2024

Copyright © 2024 Lopez Vera et al. This is an open-access publication distributed under the terms of the Creative Commons Attribution license.
